# Tumor‐Activatable Nanoparticles Target Low‐Density Lipoprotein Receptor to Enhance Drug Delivery and Antitumor Efficacy

**DOI:** 10.1002/advs.202201614

**Published:** 2022-06-24

**Authors:** Xiaomin Jiang, Wenbo Han, Jianqiao Liu, Jianming Mao, Morten J. Lee, Megan Rodriguez, Youyou Li, Taokun Luo, Ziwan Xu, Kaiting Yang, Marc Bissonnette, Ralph R. Weichselbaum, Wenbin Lin

**Affiliations:** ^1^ Department of Chemistry The University of Chicago Chicago IL 60637 USA; ^2^ Department of Radiation and Cellular Oncology and Ludwig Center for Metastasis Research The University of Chicago 5758 S Maryland Ave Chicago IL 60637 USA; ^3^ Department of Medicine Division of Biological Sciences The University of Chicago Chicago IL 60637 USA

**Keywords:** chemotherapy, colorectal cancer, drug delivery, low‐density lipoprotein receptor, nanomedicine

## Abstract

The binding of plasma proteins to nanomedicines is widely considered detrimental to their delivery to tumors. Here, the design of OxPt/SN38 nanoparticle containing a hydrophilic oxaliplatin (OxPt) prodrug in a coordination polymer core and a hydrophobic cholesterol‐conjugated SN38 prodrug on the lipid shell for active tumor targeting is reported. OxPt/SN38 hitchhikes on low‐density lipoprotein (LDL) particles, concentrates in tumors via LDL receptor‐mediated endocytosis, and selectively releases SN38 and OxPt in acidic, esterase‐rich, and reducing tumor microenvironments, leading to 6.0‐ and 4.9‐times higher accumulations in tumors over free drugs. By simultaneously crosslinking DNA and inhibiting topoisomerase I, OxPt/SN38 achieved 92–98% tumor growth inhibition in five colorectal cancer tumor models and prolonged mouse survival by 58–80 days compared to free drug controls in three human colorectal cancer tumor models without causing serious side effects. The study has uncovered a novel nanomedicine strategy to co‐deliver combination chemotherapies to tumors via active targeting of the LDL receptor.

## Graphical abstract

The core‐shell nanoparticle that containing a hydrophilic oxaliplatin prodrug in a coordination polymer core and a hydrophobic cholesterol‐conjugated SN38 prodrug on the lipid shell for active tumor targeting is developed. This tumor‐activable nanoparticle hitchhikes on low‐density lipoproteins, concentrates in tumors via LDL receptor‐mediated endocytosis, and selectively releases oxaliplatin and SN38 in lysosome, resulting superior antitumor effects.

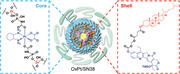

## 1 Introduction

Nanomedicines have been actively pursued to enhance tumor control and reduce treatment side effects by improving pharmacokinetics and tumor deposition of drug payloads.^[^ ^1^ ^]^ The conventional approach modifies the surfaces of 20–200 nm nanoparticles with hydrophilic molecules such as polyethylene glycol (Peg) to reduce plasma protein binding, thus minimizing mononuclear phagocytic system (MPS) uptake after systemic injection.^[^ ^2^ ^]^ Such long‐circulating nanoparticles are believed to accumulate in tumors via the enhanced permeability and retention (EPR) effect that arises from a leaky vasculature and reduced lymphatic drainage in tumors.^[^ ^3^ ^]^ However, the EPR hypothesis has been recently challenged, in part due to a low success rate of nanomedicines in the clinical setting.^[^ ^4^ ^]^ Chan and coworkers first demonstrated plasma protein coronas on gold nanoparticles and liposomes,^[^ ^5^ ^]^ and established the transport of gold nanoparticles to the tumor via an active uptake process through endothelial cells in tumors but not via the EPR effect.^[^ ^6^ ^]^ These findings have raised questions on whether and how nanoparticles alter pharmacokinetics to enhance tumor deposition of drug payloads and call for new strategies to overcome the limitations of current nanomedicine platforms.^[^ ^7^ ^]^

As key transporters of hydrophobic molecules in plasma, lipoproteins are known to interact with certain non‐pegylated nanoparticles.^[^ ^8^ ^]^ Lipoproteins are categorized into chylomicrons, very low‐density lipoproteins (VLDL), low‐density lipoproteins (LDL), and high‐density lipoproteins (HDL) based on their flotation densities and electrophoretic mobilities.^[^ ^9^ ^]^ Among these lipoproteins, LDL plays a dominant role in the transfer of cholesterol and cholesteryl esters from liver tissues to peripheral cells by LDL receptor (LDLR)‐mediated endocytosis.^[^ ^10^ ^]^ In many tumor cells, LDLR is highly expressed and has enhanced activity.^[^ ^11^ ^]^ Thus, LDL‐mimicking nanoparticles have been developed to deliver various chemotherapies to tumors, but this approach has only been moderately effective.^[^ ^12^ ^]^

Here we report the design of tumor‐responsive nanoscale coordination polymer (NCP) core‐shell nanoparticles^[^ ^13^ ^]^ for the enhanced delivery of oxaliplatin (OxPt) and SN38, an active metabolite of irinotecan, to tumors by targeting LDLR. Chemotherapy regimens containing multiple drugs,^[^ ^14^ ^]^ including FOLFOX (folinic acid, fluorouracil, and oxaliplatin), FOLFIRI (folinic acid, fluorouracil, and irinotecan), and IROX (irinotecan and oxaliplatin), are the backbone in the treatment of metastatic colorectal cancer (mCRC) which has a dismal 5‐year survival rate of 12%.^[^ ^15^ ^]^ However, these treatments have narrow therapeutic windows with severe side effects. For example, 30% of patients treated with the IROX regimen experienced severe neutropenia and 18% of patients had severe neuropathy,^[^ ^16^ ^]^ likely due to their non‐selective distribution to bone marrow and peripheral nerves, respectively. In this work, we designed an OxPt/SN38 nanoparticle with the NCP core built from a hydrophilic prodrug of OxPt and a lipid bilayer shell containing a hydrophobic prodrug of SN38. The cholesterol‐SN38 conjugate with a cleavable acetal linker (Chol‐SN38) binds to LDL and allows OxPt/SN38 to hijack the cholesterol transport function of LDL to actively deliver both prodrugs to tumors via LDLR‐mediated endocytosis. In tumors, Chol‐SN38 was selectively activated to release SN38 via both acid‐ and esterase‐catalyzed hydrolysis whereas the core of OxPt/SN38 preferentially released OxPt via disintegration in acidic endo/lysosomes followed by reduction by ascorbate and other intracellular reductants. Prolonged circulation and active transport of tumor‐responsive OxPt/SN38 significantly increased tumor deposition of both OxPt and SN38, with 4.9‐ and 6.0‐times higher tumor areas under curves (AUCs) compared to free drugs. As a result, OxPt/SN38 showed strong antitumor efficacy in both murine and human subcutaneous tumor models without causing serious side effects such as neutropenia, hepatotoxicity, and nephrotoxicity.

## 2 Results

### 2.1 Synthesis and Characterization of OxPt/SN38 and Control Particles

**Figure 1:**
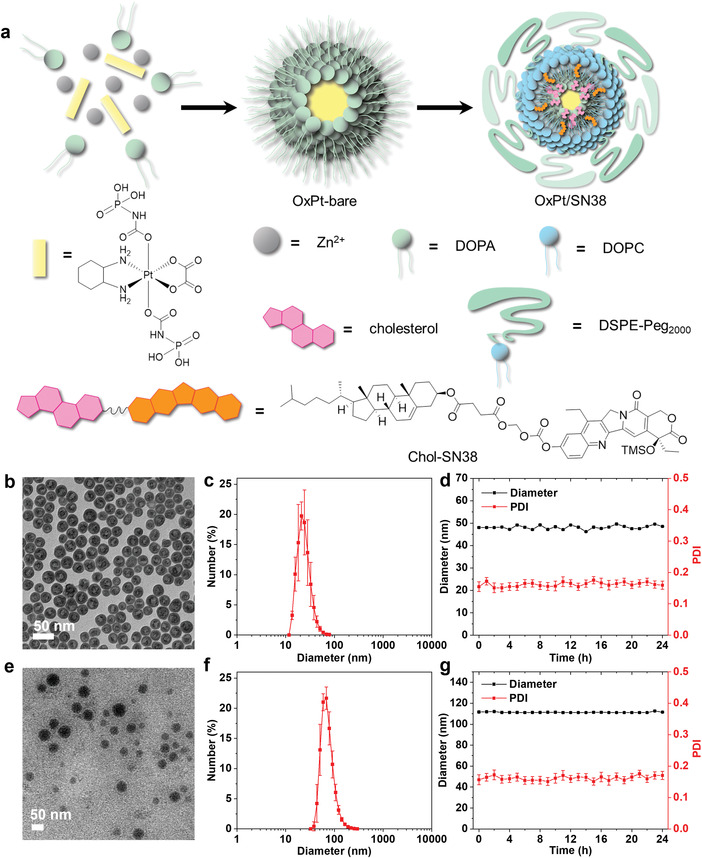
Preparation and characterization of OxPt/SN38. a) Schematic illustration of two‐step construction of core‐shell OxPt/SN38 particles via co‐polymerization of OxPt‐bp with Zn^2+^ ions and coating with Chol‐SN38, cholesterol, DOPC, and DSPE‐Peg_2000_. b) TEM image and c) number‐average diameter of OxPt‐bare. d) Stability of OxPt‐bare in THF at room temperature. e) TEM image and f) number‐average diameter of OxPt/SN38. g) Stability of OxPt/SN38 in PBS with BSA (5 mg mL^−1^) at 37 °C.

We conjugated the potent topoisomerase I inhibitor SN38 to cholesterol via an acid‐sensitive and enzymatically cleavable acetal linker to form Chol‐SN38 (Figures S1–S4, Supporting Information). We also introduced an acid‐sensitive trimethylsilyl (TMS) group to Chol‐SN38 at the O‐20 position of SN38 to disrupt the strong *π*‐*π* stacking of planar SN38 moieties^[^ ^17^ ^]^ and facilitate the formation of a stable lipid coating. OxPt/SN38 was prepared in two steps (**Figure** **1**a). First, Pt(dach)(oxalate)(bisphosphoramidic acid) (OxPt‐bp)^[^ ^13c^ ^]^ was co‐polymerized with Zn^2+^ ions in the presence of DOPA in a reverse microemulsion containing Triton X‐100/hexanol/cyclohexane to form DOPA‐capped NCP particles (OxPt‐bare). Chol‐SN38 was then incorporated into the lipid layer together with cholesterol, DOPC, and DSPE‐Peg_2000_ on the surface of OxPt‐bare to form core‐shell OxPt/SN38 particles. OxPt/SN38 particles with an OxPt:SN38 molar ratio of 1:1.8 were used for all studies in this work.

OxPt‐bare particles were characterized as spherical by transmission electron microscopy (TEM, Figure 1b) with a Z‐average diameter of 49.0 nm and a polydispersity index (PDI) of 0.17 by dynamic light scattering (DLS, Figure 1c and Table S1, Supporting Information). OxPt‐bare showed high stability in tetrahydrofuran at room temperature (Figure 1d). OxPt/SN38 particles showed spherical morphology (Figure 1e) with a Z‐average diameter of 111.6 nm and PDI of 0.15 (Figure 1f and Table S1, Supporting Information). OxPt/SN38 particles were stable in PBS containing 5 mg mL^−1^ bovine serum albumin (BSA) with no changes in size and PDI at 37 °C (Figure 1g). Monotherapy particles with OxPt‐bp in the core, OxPt NCP, and with Chol‐SN38 on the shell, ZnP/SN38, were prepared in the absence of Chol‐SN38 or with pyrophosphate replacing OxPt prodrug, respectively. Other control particles used in the present study were similarly prepared and characterized (Figures S5 and S6 and Table S1, Supporting Information).

### 2.2 OxPt/SN38 Binds and Transfers Chol‐SN38 to LDL

**Figure 2:**
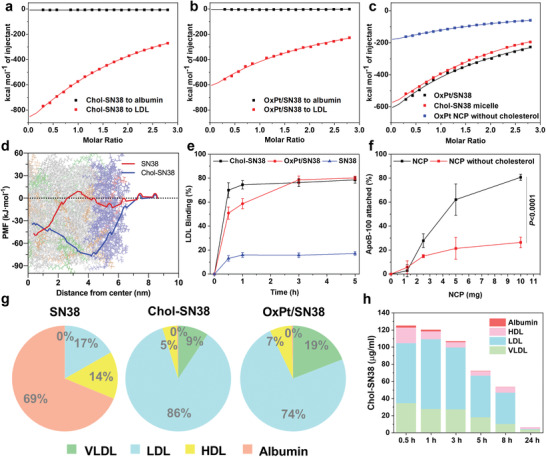
OxPt/SN38 transfers Chol‐SN38 to LDL. Integrated binding heats upon injection of a) Chol‐SN38 into an LDL or albumin solution, b) OxPt/SN38 into an LDL or albumin solution, and c) Chol‐SN38 micelle or OxPt NCP without cholesterol into an LDL solution by ITC. d) The PMF for transferring SN38 (red line) and Chol‐SN38 (blue line) from bulk water to the lipid core of an LDL slice from MD simulations. The plots are superimposed onto a snapshot of an equilibrated LDL slice. e) Time‐dependent binding of SN38 or Chol‐SN38 to LDL and transfer of Chol‐SN38 from OxPt/SN38 to LDL in rat plasma. f) Concentration‐dependent ApoB‐100 binding to ZnP NCP with and without cholesterol, n = 3. g) Distributions of SN38, Chol‐SN38, and Chol‐SN38 from OxPt/SN38 in different lipoproteins of rat plasmas by ultracentrifugation separation. h) Pharmacokinetic profile of Chol‐SN38 from OxPt/SN38 in rat plasma and its lipoprotein distribution after intravenous injection of OxPt/SN38 at a Chol‐SN38 dose of 14.4 mg kg^−1^. Data are expressed as means ± SD by Student's two‐tailed t‐test.

LDL binds and transports cholesterol and cholesteryl esters to peripheral cells. Chol‐SN38 is strongly bound to LDL with an association constant (*K_a_*) of 4.97 × 10^5^ m ^−1^ by isothermal titration calorimetry (ITC, **Figure** **2**a). Titration of LDL with OxPt/SN38 led to exothermic binding with a *K_a_* of 3.34 × 10^4^ m ^−1^. Both Chol‐SN38 and OxPt/SN38 showed a 1000 times lower affinity to albumin (Figure 2b, Figure S7, and Table S2, Supporting Information). We further determined the binding of two control particles, OxPt NCP without cholesterol and Chol‐SN38 micelle (without the NCP core), to LDL. While Chol‐SN38 micelle showed similar binding affinity to LDL as OxPt/SN38, OxPt NCP without cholesterol showed 10 times lower binding affinity to LDL (Figure 2c, Figure S7, and Table S2, Supporting Information). These results suggest that cholesterol and Chol‐SN38 mediate the binding of NCP to LDL.

Molecular dynamics (MD) simulations were next performed to elucidate atomic‐level interactions between LDL and Chol‐SN38 (Figure S8 and Table S3, Supporting Information).^[^ ^18^ ^]^ We computed the potential of the mean force for transferring Chol‐SN38 from bulk water to the lipid core of LDL. The potential of mean force for Chol‐SN38 significantly decreased at almost every location in LDL and decreased the most by ≈80 kJ mol^−1^ at the interface of the hydrophobic core and hydrophilic shell of 1‐palmitoyl‐2‐oleoyl‐snglycero‐3‐phosphocholine (POPC) and 1‐palmitoyl‐2‐hydroxy‐sn‐glycero‐3‐phosphocholine (lyso‐PC) (Figure 2d). In contrast, the potential mean force for SN38 did not decrease until approaching the center of the hydrophobic core of LDL. These results confirm attractive hydrophobic/hydrophobic interactions between Chol‐SN38 and the LDL core.

We then determined the binding kinetics of Chol‐SN38, OxPt/SN38, and SN38 to LDL in rat plasma by LC‐MS. While Chol‐SN38 quickly bound to LDL in the plasma and reached equilibrium with 74.5% Chol‐SN38 binding to LDL within 1 h, OxPt/SN38 showed slower transfer of Chol‐SN38 to LDL but reached a similar level in 3 h (Figure 2e). In contrast, SN38 showed only 17.1% binding to LDL.

The distributions of SN38, Chol‐SN38, and OxPt/SN38 in various lipoproteins in rat plasmas were quantified by LC‐MS. The lipoproteins were separated based on their densities by NaBr gradient ultracentrifugation. SN38 was mainly distributed in albumin (68.8%) and only slightly distributed in LDL (16.8%), while Chol‐SN38 was mostly distributed to LDL (85.8%) with <1% distribution in albumin (Figure 2g and Table S4, Supporting Information). Interestingly, Chol‐SN38 in OxPt/SN38 was efficiently transferred to lipoproteins, with 74.1% in LDL, 19.4% in VLDL, and 6.1% in HDL but <1% in albumin.

In vivo pharmacokinetics of OxPt/SN38 on SD/CD rats showed long blood circulation of Chol‐SN38 with a half‐life (t_1/2_) of 9.7 h and an area under the curve (AUC_0→t_) of 1874.6 µg h mL^−1^ (Table S5, Supporting Information). We also assayed the distribution of Chol‐SN38 in different plasma proteins at each time point and found an AUC_0→t_ of 871.6 µg h mL^−1^ for LDL‐bound Chol‐SN38, which represented 46.5% of the total Chol‐SN38 AUC_0→t_ (Figure 2h). Taken together, these studies show strong binding of Chol‐SN38 to LDL and the transfer of Chol‐SN38 from OxPt/SN38 to LDL in plasma.

### 2.3 LDLR‐Mediated Endocytosis Determines OxPt/SN38 Uptake by Tumor Cells

Apo B‐100 protein in LDL is a strong ligand for LDLR and is responsible for the efficient transfer of cholesterol to peripheral cells via LDLR‐mediated endocytosis.^[^ ^19^ ^]^ We determined the adsorption of Apo B‐100 to ZnP control NCP by BCA assay. As ZnP concentration increased, the amount of adsorbed Apo B‐100 increased (Figure 2f), with 80.7% Apo B‐100 captured by 10 mg ZnP particles. The Apo B‐100 binding capacity decreased to 26.4% for ZnP particles without cholesterol. We mixed ZnP NCP with mouse plasma and then analyzed the adsorption of Apo B‐100 on ZnP particles by western blot. We found that Apo B‐100 was adsorbed on ZnP particles, and the level of Apo B‐100 adsorption correlated with ZnP concentration (Figure S9, Supporting Information).

**Figure 3:**
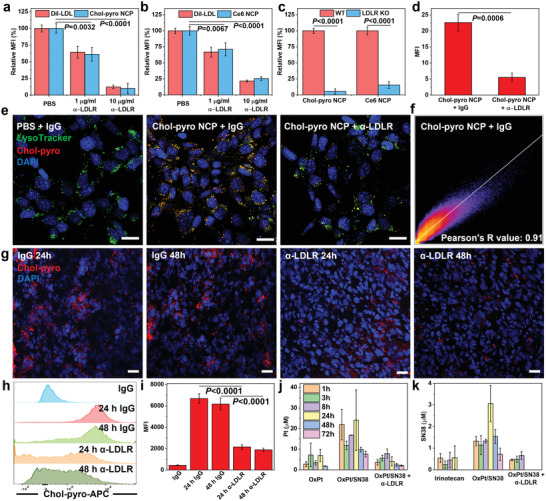
LDLR‐mediated endocytosis determines NCP particle uptake by tumor cells. Uptake of a) Chol‐pyro NCP and b) Ce6‐NCP by MC38 cells after LDLR blockade with 1 or 10 µg mL^−1^ *α*‐LDLR. The Dil‐LDL uptake served as control. c) Cellular uptake of Chol‐pyro‐NCP and Ce6‐NCP on WT and LDLR KO MC38 cells. CLSM d) statistical analysis and e) images of Chol‐SN38 uptaken by MC38 cells 24 h after treatment with 10 µg mL^−1^ IgG or *α*‐LDLR. Scale bar: 20 µm. f) Lysosome was stained with LysoTracker and the colocalization of lysosome and Chol‐SN38 was evaluated based on Pearson's R value. g) Immunofluorescence analysis showing the in vivo tumor uptake of Chol‐pyro NCP at 24 and 48 h with 1 µg of IgG or *α*‐LDLR. Scale bar: 20 µm. h) Flow cytometry results and i) mean fluorescent intensity of Chol‐pyro NCP tumor uptake at 24 and 48 h post i.v. injection with 1 µg of IgG or *α*‐LDLR. Time‐dependent j) Pt and k) SN38 accumulation after i.v. injection of OxPt (3.5 mg kg^−1^) plus irinotecan (6.2 mg SN38/kg equivalent), OxPt/SN38 (3.5 mg OxPt/kg equivalent, 6.2 mg SN38/kg equivalent) to MC38‐bearing mice with and without 1 µg of intratumorally injected *α*‐LDLR. Data are expressed as means ± SD. The data were analyzed by one‐way analysis of variance (ANOVA; a,b) with Tukey's multiple comparison test, or Student's two‐tailed t‐test (c,d,i).

The uptake of NCP particles by tumor cells via LDLR‐mediated endocytosis was confirmed using fluorescently labeled LDL (Dil‐LDL) and cholesterol‐pyropheophytin a (Chol‐pyro)‐loaded NCP as a surrogate for Chol‐SN38 in the shell and chlorin E6 (Ce6)‐loaded NCP as a surrogate for OxPt in the core (Figure S10, Supporting Information). The uptake levels of both Chol‐pyro NCP and Ce6 NCP by murine CRC MC38 cells decreased with LDLR blockade by an anti‐LDLR antibody (*α*‐LDLR) in a dose‐proportional manner (**Figure** **3**a,b). With LDLR blockade, Dil‐LDL uptake by MC38 cells decreased by a similar percentage as Chol‐pyro NCP and Ce6 NCP. Compared to wildtype (WT) MC38 cells, LDLR knockout (KO) MC38 cells showed much lower uptake of Chol‐pyro NCP (5%) and Ce6 NCP (15%) (Figure 3c and Figure S11, Supporting Information). These results suggest that LDLR‐mediated endocytosis plays a major role in the cellular uptake of NCPs.

We also visualized co‐localization of Chol‐pyro NCP and LysoTracker and determined cellular uptake levels of Chol‐pyro by confocal laser scanning microscopy (CLSM, Figure 3d,e and Figure S12, Supporting Information). After 24 h incubation, Chol‐pyro (red) colocalized with endo/lysosomes with a Pearson's R value of 0.91 (Figure 3f).

To investigate drug accumulation in tumors by LDLR‐mediated endocytosis, we determined Chol‐pyro fluorescence signals in tumor slices from MC38‐bearing C57BL/6 mice 24 or 48 h after intravenous injection of 200 µg of Chol‐pyro NCP with and without intratumoral injection of 1 µg *α*‐LDLR. The administration of *α*‐LDLR decreased Chol‐pyro signals by 52.9% and 60.2% at 24 and 48 h time points, respectively (Figure 3g and Figure S13, Supporting Information). The tumors were also digested into single cell suspensions for flow cytometric analysis of intracellular Chol‐pyro signals as a function of LDLR blockade. Flow cytometric results showed that Chol‐pyro levels decreased by 67.7% and 69.0% in tumor cells with LDLR blockade at 24 and 48 h time points, respectively (Figure 3h, i).

We next quantitatively determined drug accumulation in MC38 tumors following intravenous injection 3.5 mg kg^−1^ OxPt/SN38 (based on OxPt equivalents) with and without concurrent intratumoral injection of 1 µg *α*‐LDLR (Figure 3j,k and Table S6, Supporting Information). Without LDLR blockade, OxPt/SN38 exhibited a Pt AUC _0→t_ of 290.3 h µg mL^−1^ and an SN38 AUC _0→t_ of 50.8 h µg mL^−1^ in the tumors, which are 4.9 times that of free OxPt and 6 times that of free irinotecan at equivalent OxPt and SN38 doses, respectively. With LDLR blockade, the OxPt AUC_0→t_ decreased by 72% and the SN38 AUC_0→t_ decreased by 90%, lowering the drug accumulation levels to those of free OxPt and irinotecan, respectively. While OxPt/SN38 maintained intratumoral drug concentrations above IC_50_ values for CT26 and MC38 murine CRC cells and for HT29, HCT116, and SW480 human CRC cells for 72 h (Tables S7 and S8, Supporting Information), OxPt/SN38 with LDLR blockade, like the free drugs, failed to maintain intratumoral drug concentrations above IC_50_ values beyond 24 h. These results demonstrate that OxPt/SN38 significantly increases intratumoral OxPt and SN38 concentrations by targeting the LDLR through binding and transferring Chol‐SN38 to LDL in vivo.

### 2.4 Tumor‐Activated Release of OxPt and SN38 from OxPt/SN38

**Figure 4:**
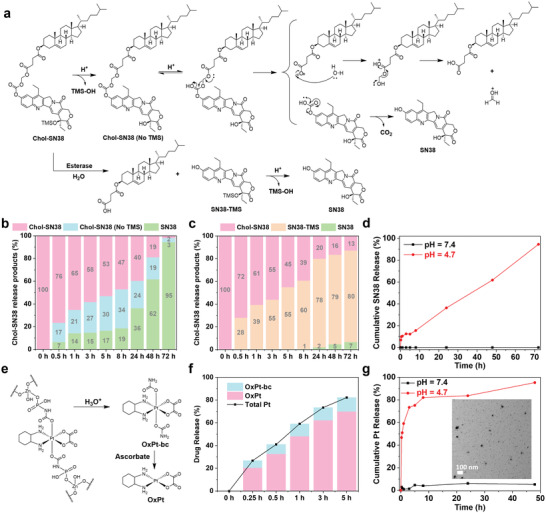
Release of SN38 and OxPt from OxPt/SN38. a) Proposed SN38 release via acid‐catalyzed hydrolysis and esterase‐mediated cleavage. Percentages of Chol‐SN38 and released products from OxPt/SN38 at b) pH = 4.7 in PBS and c) pH = 7.4 PBS with 10 unit mL^−1^ esterase throughout 72 h at 37 °C. d) Percentages of SN38 released from OxPt/SN38 particle when incubated in PBS at pH = 4.7 or pH = 7.4 throughout 72 h at 37 °C. e) Release of OxPt from OxPt/SN38 via hydrolysis and reduction by ascorbate. f) Total Pt and OxPt release profiles from OxPt/SN38 particles when incubated in PBS with pH = 4.7 and 5 mm ascorbate at 37 °C. g) Time‐dependent Pt release from OxPt/SN38 particle when incubated in PBS at pH = 4.7 or pH = 7.4 at 37 °C. The inset showed the TEM image of disintegrated OxPt/SN38 particles after incubation in PBS at pH = 4.7 and 37 °C for 5 h.

We next examined the pH‐triggered release of SN38 from Chol‐SN38 in endo/lysosomes. At pH = 4.7, Chol‐SN38 from OxPt/SN38 was hydrolyzed at both the 20‐O‐TMS and carbonate linkages to release SN38 in 95% yield in 72 h (**Figure** **4**a,b,d, and Figure S14, Supporting Information). In contrast, a negligible amount of SN38 was released from OxPt/SN38 at pH = 7.4 in 72 h. As carboxylesterase in tumor cells contributes to the release of SN38 from irinotecan,^[^ ^20^ ^]^ we tested the release of SN38 from OxPt/SN38 in PBS with 10 unit mL^−1^ esterase. Chol‐SN38 was gradually hydrolyzed to SN38‐TMS (Figure 4c), which was further converted to SN38. These results indicate a dual activation mechanism for the release of SN38 from OxPt/SN38 in tumors: the 20‐OTMS and carbonate linkages of Chol‐SN38 are successively hydrolyzed at low pH to yield SN38 and the carbonate and SN38‐TMS linkages are hydrolyzed by the esterase and via proton/TMS exchange, respectively, to generate SN38. The tumor‐activated release of SN38 could potentially minimize normal tissue exposure to SN38.

The NCP core formed by Zn^2+^ ions and OxPt‐bp is known to disintegrate in acidic environments.^[^ ^13c^ ^]^ At pH = 7.4, OxPt/SN38 released less than 6% Pt over a course of 48 h. At pH = 4.7, OxPt/SN38 particles quickly disintegrated to release Pt(dach)(oxalate)(biscarbamate) (OxPt‐bc) (Figure 4e,g). In the presence of 5 mm ascorbate, the released OxPt‐bc was efficiently reduced to form OxPt (Figure 4e,f). These results demonstrate the triggered release of both SN38 and OxPt from OxPt/SN38 in cancer cells.

### 2.5 In Vitro Mechanisms of Cell Death

In vitro cytotoxicity of OxPt/SN38 and monotherapy control particles was tested in CRC cells (Tables S7 and S8, Supporting Information). OxPt/SN38 showed strong synergy of the two drugs in CT26 and MC38 cells, lowering OxPt IC_50_ values by three‐ to four‐folds compared to OxPt NCP and halving Chol‐SN38 IC_50_ values compared to ZnP/SN38. OxPt/SN38 showed similar synergistic cytotoxicity in HT29, HCT116, and SW480 cells, and was more cytotoxic than either OxPt or irinotecan. Interestingly, although SN38‐TMS showed potent cytotoxicity with IC_50_ values of 2.52 and 3.04 µm for CT26 and MC38 cells, respectively, this cytotoxicity likely came from SN38 generated from hydrolysis of SN38‐TMS, since analogous SN38 derivates 20‐O‐tert‐butyl‐SN38 (SN38‐^t^Bu) and 20‐O‐Boc‐SN38 (SN38‐Boc) showed no cytotoxicity in CT26 and MC38 at concentrations up to 300 µm (Figures S15 and S16, Table S7, Supporting Information).

**Figure 5:**
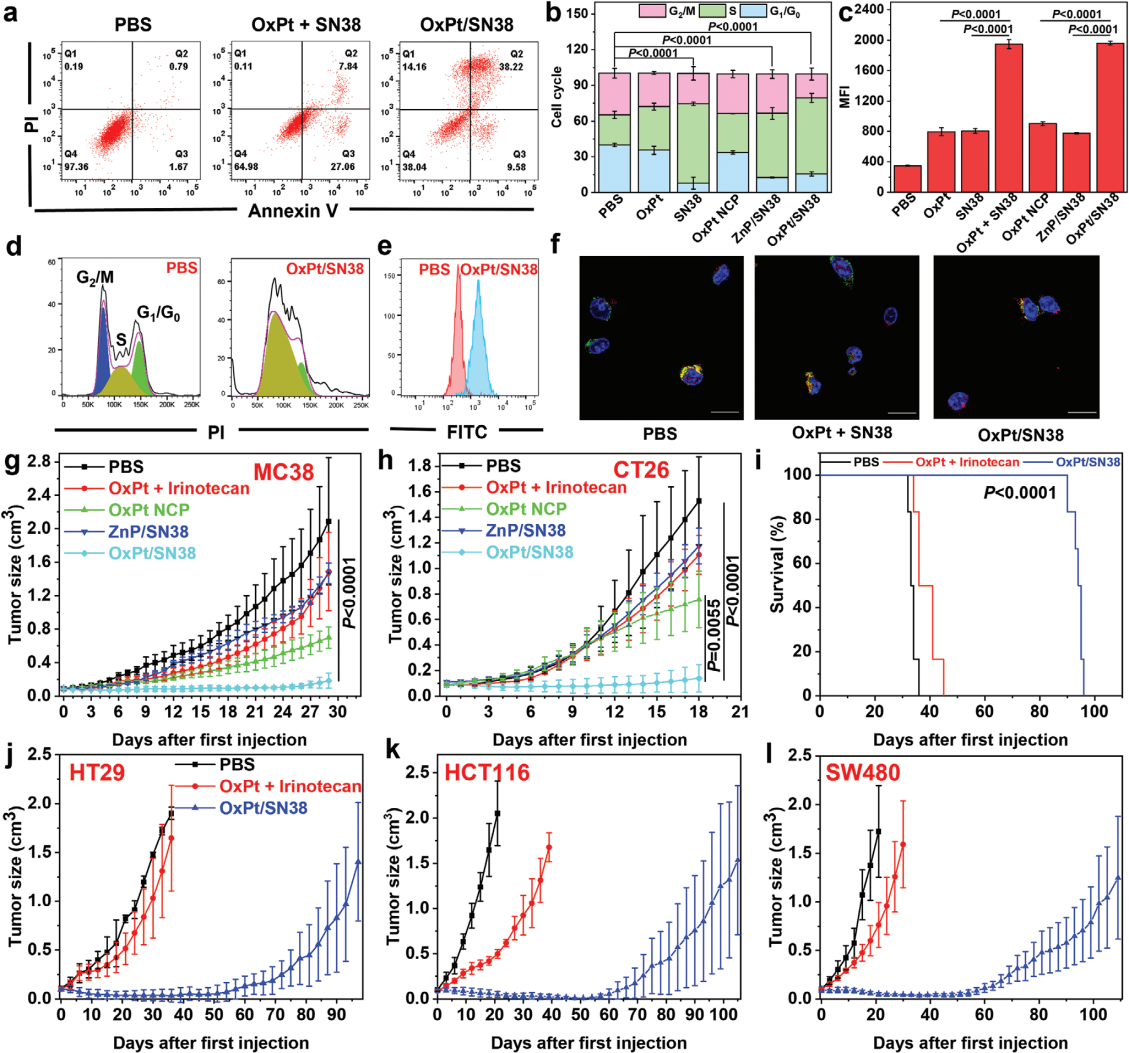
In vitro programmed cell death and in vivo anticancer efficacy. a) Apoptosis induced by OxPt plus SN38 or OxPt/SN38. After treatment, cells were stained by Alexa Fluor 488‐labelled Annexin V and PI and analyzed by flow cytometry. b,d) Cell cycle arrest caused by OxPt/SN38. Treated cells were fixed with 70% ethanol overnight, treated with RNase A, stained with PI, and analyzed by flow cytometry, n = 3. c,e) Flow cytometry analysis with JC‐1 staining of MMPs of MC38 cells treated with PBS, OxPt plus SN38 or OxPt/SN38, n = 3. f) Release of cytochrome c from mitochondria in MC38 cells treated with OxPt plus irinotecan or OxPt/SN38. Mitochondria (red fluorescence) and cytochrome c (green fluorescence) were stained by MitoTracker Red CMXRos and anti‐cytochrome c antibody, respectively. Scale bar: 20 µm. Tumor growth curves of g) MC38 tumors in C57BL/6 mice and h) CT26 tumors in BALB/c mice. PBS, OxPt plus irinotecan, OxPt NCP, ZnP/SN38, or OxPt/SN38 was i.v. injected once every 3 days (Q3D) at doses of 3.5 mg kg^−1^ OxPt or equivalent, 20.2 mg kg^−1^ irinotecan (11.7 mg kg^−1^ SN38 equivalent), and/or 15.9 mg Chol‐SN38/kg (6.2 mg kg^−1^ SN38 equivalent) to MC38 model for up to 8 doses and to CT26 model for up to 6 doses, n = 6. i) Survival curves of HT29 model and j) tumor growth curves of HT29, k) HCT116, and l) SW480 models in nude mice after Q3D treatment with PBS, OxPt/SN38, or OxPt plus for up to 16 doses. The doses are the same as MC38 and CT26 models. Data are expressed as means ± SD. The data were analyzed by one‐way ANOVA with Tukey's multiple comparison test.

Alexa Fluor 488‐Annexin V and PI staining showed both OxPt and SN38 induced programmed cell death by apoptosis/necrosis. The combination of OxPt and SN38 increased the percentages of both early apoptotic Annexin V^+^/PI^−^ cells and late apoptotic/necrotic Annexin V^+^/PI^+^ cells (**Figure** **5**a and Figure S17, Supporting Information).

MC38 cells treated with OxPt NCP and ZnP/SN38 showed 32.6% and 53.9% S‐phase arrest, respectively (Figure 5b,d and Figure S18, Supporting Information). In comparison, 25.3% cells were in S‐phase for PBS control. OxPt/SN38 showed a stronger S‐phase arrest of 63.8%. OxPt/SN38 particles thus effectively caused DNA damage and inhibited DNA replication for anti‐proliferative effects.

We next evaluated mitochondrial disruption caused by OxPt/SN38 through flow cytometry analysis of JC‐1 staining and CLSM imaging of cytochrome c release. Treatment of MC38 cells with OxPt/SN38 for 24 h resulted in a fivefold increase in the depolarization of the mitochondrial membrane potential (MMP) compared to PBS control (Figure 5c,e and Figure S19, Supporting Information) and the release of cytochrome c from mitochondria with a Pearson's R value of 0.27 between MitoTracker (red fluorescence) and FITC‐conjugated anti‐cytochrome c antibody (green fluorescence) (Figure 5f and Figures S20 and S21, Supporting Information).

### 2.6 Anticancer Efficacy in Colorectal Adenocarcinoma

The in vivo anticancer efficacy of OxPt/SN38 was evaluated in five subcutaneous tumor models. When the tumors reached 80–120 mm^3^ in volume, mice were intravenously injected with the indicated treatments every three days (Q3D). OxPt/SN38, OxPt plus irinotecan, OxPt NCP, and ZnP/SN38 were dosed at 3.5 mg kg^−1^ OxPt equivalent and 15.9 mg kg^−1^ Chol‐SN38 (equivalent to 6.2 mg kg^−1^ SN38), 3.5 mg kg^−1^ OxPt and 20.2 mg kg^−1^ irinotecan (11.7 mg kg^−1^ SN38 equivalent), 3.5 mg kg^−1^ OxPt equivalent, and 15.9 mg kg^−1^ Chol‐SN38 (equivalent to 6.2 mg kg^−1^ SN38), respectively (Figure 5g,h,j–l and Figures S22, S23, and S25, Supporting Information). In all tested models, OxPt/SN38 led to significantly greater inhibition/regression of tumor growth with minimal toxicity as judged by comparable body weight gain, normal histology of major organs including heart, liver, spleen, lung and kidney, and normal liver and renal function tests (Figure S27, Supporting Information).

MC38 tumor‐bearing C57BL/6 mice following 8 intravenous injections of OxPt/SN38 showed 92.2% tumor growth inhibition (TGI). Despite a 1.9‐fold higher SN38 equivalent dose, OxPt plus irinotecan provided a modest TGI of 22.3%. OxPt NCP and ZnP/SN38 only slightly inhibited tumor growth with TGI values of 66.9% and 16.4%, respectively. The mice tolerated the treatments well with stable body weights for all groups (Figure S24a, Supporting Information).

Neutropenia is the most serious side effect of the IROX regimen with 30% mCRC patients experiencing severe neutropenia after repeated doses of OxPt plus irinotecan. Blood samples were collected from MC38 tumor‐bearing C57BL/6 mice following 8 intravenous injections of PBS or OxPt/SN38, or OxPt plus irinotecan. The absolute neutrophil count (ANC) significantly decreased in mice treated with OxPt plus irinotecan but slightly increased for mice treated with OxPt/SN38 compared to PBS control (Figure S28a, Supporting Information). Thus, OxPt/SN38 treatment prevented the dose‐limiting toxicity of severe neutropenia in the IROX regimen.

We also determined liver and kidney functions by measuring the levels of alanine aminotransferase (ALT), aspartate aminotransferase (AST), and serum creatinine levels in the sera of C57BL/6 mice after 8 and 15 doses of PBS or OxPt/SN38 (Figure S28b–d, Supporting Information). The AST and ALT levels for mice treated with OxPt/SN38 slightly increased over PBS control but remained within the normal ranges. The creatinine level remained unchanged in mice treated with OxPt/SN38 (0.24 ± 0.07mg dL^−1^) compared to those treated with PBS (0.22 ± 0.01 mg dL^−1^). These results indicate that repeated doses of OxPt/SN38 did not cause hepatotoxicity and nephrotoxicity in mice.

In the CT26 model, OxPt/SN38, OxPt plus irinotecan, OxPt NCP, and ZnP/SN38 showed TGI of 90.9%, 27.6%, 51.6%, and 29.1%, respectively. Thus OxPt/SN38 showed a strong synergy of the two drugs with enhanced anticancer efficacy compared to the other treatment groups. The treatments were well tolerated with no differences in body weights among the groups.

OxPt/SN38 showed excellent anticancer efficacy in mouse tumor xenograft models of human colorectal adenocarcinomas. Tumor‐bearing mice were intravenously injected with PBS, OxPt/SN38, or OxPt plus irinotecan for 16 doses. For HT29 model, OxPt/SN38 regressed the tumors showing a TGI of 99.4% at the PBS endpoint. OxPt plus irinotecan slightly inhibited the tumors to provide a TGI of 32.2%. After the cessation of OxPt/SN38 treatment on Day 46, growth inhibition of HT29 tumors persisted for another 12 days but tumors eventually resumed growth after Day 57. OxPt/SN38 and OxPt plus irinotecan treatments extended the median survival from 33 days for PBS‐treated control group to 94 and 36 days, respectively (Figure 5i and Figure S26, Supporting Information). ZnP/SN38 at a dose of 36 mg kg^−1^ Chol‐SN38 also effectively inhibited HT29 tumor growth with a TGI of 87.0% (Figure S29, Supporting Information). The mice in all groups tolerated the treatments well.

OxPt/SN38 also showed potent antitumor efficacy against HCT116 and SW480 tumor xenografts. For HCT116 model, OxPt/SN38 regressed tumors to afford a TGI of 97.6% at the PBS endpoint on Day 21, while OxPt plus irinotecan showed a TGI of 75.8%. OxPt/SN38 and OxPt plus irinotecan extended mouse survival from 20 days for PBS group to 106 and 41 days, respectively. For SW480 model, OxPt/SN38 regressed tumors to yield a TGI of 96.9% at the PBS endpoint on Day 17 while OxPt plus irinotecan gave a moderate TGI of 57.9%. OxPt/SN38 and OxPt plus irinotecan extended mouse survival from 20 days for PBS group to 112 and 32 days, respectively. With unique pharmacokinetics and pharmacodynamics, excellent antitumor efficacy in multiple CRC tumor models, and good safety profiles, OxPt/SN38 is primed for clinical testing on CRC patients.

### 2.7 LDLR‐Mediated Endocytosis Determines the Anticancer Efficacy of OxPt/SN38

**Figure 6:**
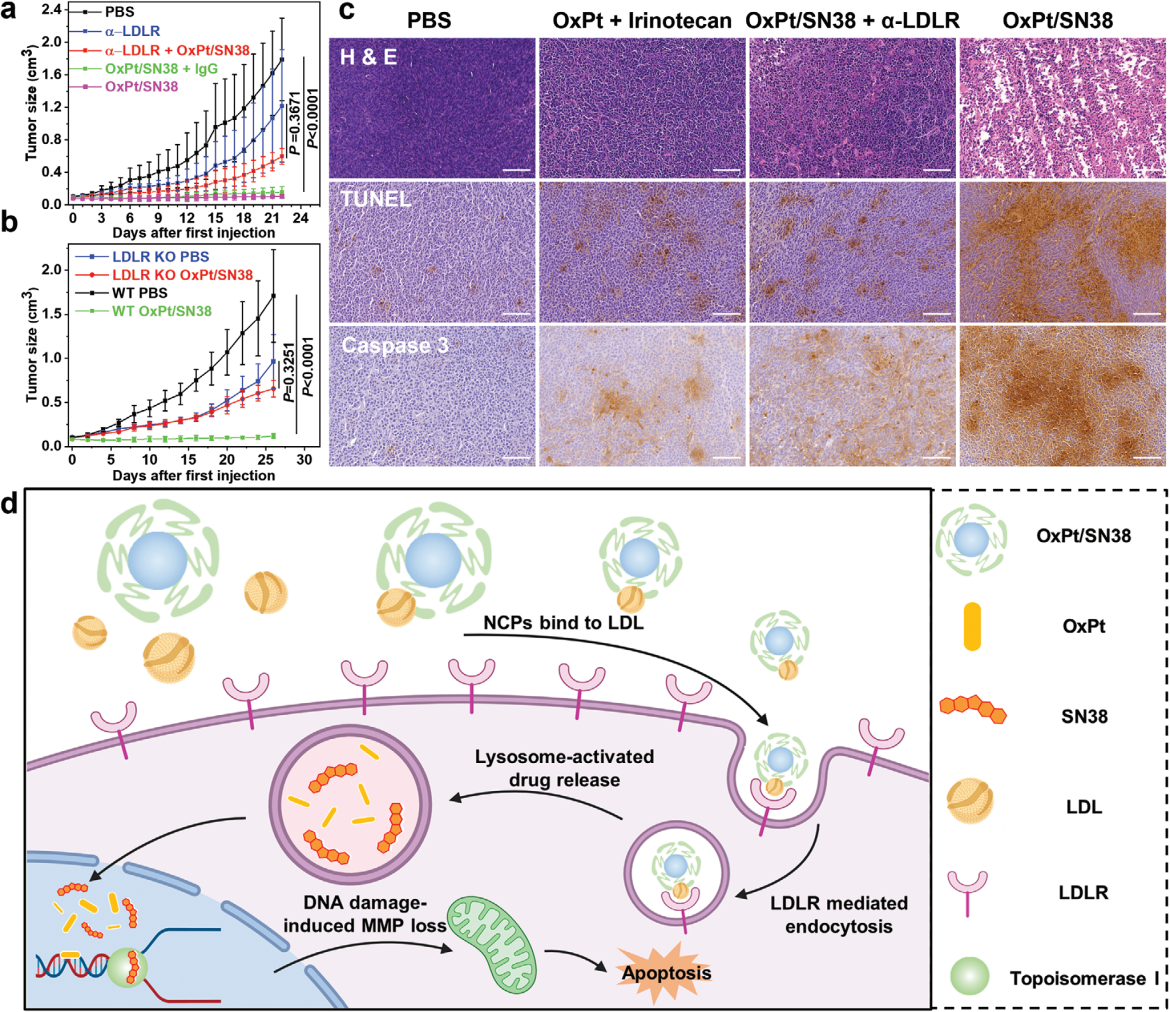
LDLR‐mediated endocytosis determines the anticancer efficacy of OxPt/SN38. a) Anticancer efficacy of OxPt/SN38 with intratumorally injected 1 µg IgG or *α*‐LDLR on MC38 tumor‐bearing C57BL/6 mice at a dose of 3.5 mg OxPt/kg equivalent. n = 6. b) Anticancer efficacy of OxPt/SN38 on WT and LDLR KO MC38 tumor‐bearing C57BL/6 mice at a dose of 3.5 mg OxPt/kg equivalent. n = 6. c) H&E staining (top), TUNEL (middle), and Caspase 3 (bottom) showing differences in apoptosis and necrosis of MC38 tumors after OxPt/SN38 treatments with intratumorally injected IgG or *α*‐LDLR. Scale bar: 100 µm. d) Scheme showing OxPt/SN38 delivery by hitchhiking LDL and anticancer mechanism. Once injected intravenously, OxPt/SN38 binds to LDL and transfers Chol‐SN38 to LDL. LDL‐bound OxPt/SN38 is taken up by cancer cells through LDLR‐mediated endocytosis. Acidic endo/lysosomal environment triggers the release of OxPt and SN38 from OxPt/SN38. The released OxPt and SN38 synergistically inhibit DNA replication by crosslinking DNA bases and binding to topoisomerase I, respectively. The DNA damage causes MMP disruption, resulting in the early apoptosis of cancer cells. Data are expressed as means ± SD. The data were analyzed by one‐way ANOVA with Tukey's multiple comparison test. (d) was created with BioRender.com.

To evaluate the role of LDLR‐mediated endocytosis on anticancer efficacy, we determined TGI of intravenously injected OxPt/SN38 with concurrent LDLR blockade by intratumorally injecting 1 µg *α*‐LDLR on a Q3D schedule (**Figure** **6**a, and Figure S30a, Supporting Information). While *α*‐LDLR slowed tumor growth with a TGI of 40.2% over the immunoglobin G (IgG) control, concurrent LDLR blockade significantly weakened the anti‐tumor efficacy of OxPt/SN38 treatment with a TGI of 51.6% compared to a TGI of 91.2% for OxPt/SN38 treatment with control IgG injection. The results were corroborated by the tumor weights at the endpoint: OxPt/SN38 treatment with concurrent IgG injection showed a TGI of 92% while OxPt/SN38 treatment with concurrent LDLR blockade showed a TGI of 57% (Figure S30b, c, Supporting Information). We confirmed the role of LDLR in OxPt/SN38 anti‐tumor efficacy using LDLR KO MC38 tumor cells. The anticancer efficacy of OxPt/SN38 was nearly abrogated in LDLR KO MC38 tumors with no significant difference between OxPt/SN38 and PBS groups (Figure 6b and Figures S31 and S32, Supporting Information).

We further examined in vivo cytotoxicity in tumor cells through histopathological analysis (Figure 6c). H&E staining showed severe necrosis in MC38 tumors treated with OxPt/SN38 but much less necrosis in MC38 tumors treated with OxPt/SN38 and *α*‐LDLR. TUNEL and Caspase 3 IHC staining showed strong apoptosis induced by OxPt/SN38, but greatly reduced apoptosis when LDLR was blocked. These results show that LDLR‐mediated endocytosis is key to the tumor uptake of OxPt/SN38 in vivo and plays a crucial role in antitumor efficacy.

## 3 Discussion

For decades, cytotoxic anticancer drugs such as SN38 (LogP = 3.37) have been modified with hydrophilic groups to render them more soluble in an aqueous solution.^[^ ^21^ ^]^ Conversion of water‐insoluble SN38 into water‐soluble irinotecan hydrochloride (LogP = −0.45)^[^ ^22^ ^]^ represents one of the most successful examples of anticancer drug design. Binding of hydrophobic chemotherapeutics to plasma proteins presents an alternative approach and can actively target highly expressed receptors. For example, nab‐paclitaxel (albumin‐paclitaxel nanoparticles) showed better efficacy than paclitaxel in some tumors, presumably via targeting the Gp60 transcytosis pathway in endothelial cells and binding to secreted protein, acidic and rich in cysteine (SPARC) in the tumor extracellular matrix.^[^ ^23^ ^]^ In this work, we uncovered a novel strategy to co‐deliver combination chemotherapies via active targeting of LDLR in tumors. We conjugated SN38 to highly hydrophobic cholesterol (LogP = 7.02) through a labile acetal linkage to hijack LDL for tumor targeting.

Systemically injected nanotherapeutics have long been shown to prolong blood circulation over their parent drugs, which are believed to operate through the EPR effect.^[^ ^24^ ^]^ Core‐shell NCP particles allowed the loading of both hydrophilic OxPt‐bp and hydrophobic Chol‐SN38. We discovered strong binding of Chol‐SN38 to LDL and transfer of Chol‐SN38 from OxPt/SN38 to LDL for active transport to tumors via LDLR‐mediated endocytosis. SN38 was selectively released inside tumor cells via acid‐ and esterase‐catalyzed hydrolysis. On the other hand, the NCP core of OxPt/SN38 adsorbed LDL for tumor targeting via the LDLR pathway and released OxPt in tumors via disintegration in acidic endo/lysosomes and reduction by ascorbate and other intracellular reductants. As a result, OxPt/SN38 significantly increased tumor deposition of OxPt by a factor of 4.9 over OxPt and SN38 by a factor of 6.0 over irinotecan at equivalent doses.

IROX is one of the standard chemotherapy regimens for mCRC due to the synergistic action of OxPt and SN38 on CRC cells.^[^ ^25^ ^]^ By increasing intratumoral OxPt and SN38 concentrations, OxPt/SN38 maximized the synergy between OxPt and SN38 on murine and human CRC cells. OxPt/SN38 simultaneously crosslinked DNA with OxPt and inhibited topoisomerase I with SN38, resulting in severe DNA damage, inhibition of DNA replication, and disruption of mitochondrial membranes. OxPt/SN38 achieved >92% TGI of MC38 and CT26 tumor models and >97% TGI of HT29, HCT116, and SW480 tumor models. OxPt/SN38 also prolonged mouse survival by 61–92 days compared to PBS control and by 58–80 days compared to OxPt plus irinotecan in these tumor models.

In summary, we developed a novel strategy to actively target tumors via LDLR‐mediated endocytosis with core‐shell NCPs for enhanced drug accumulation in tumors. With tumor‐responsive release of SN38 and OxPt, OxPt/SN38 exhibited 6.0‐ and 4.9‐times higher tumor AUCs over free drugs and achieved 92–98% TGI in five CRC tumor models and significantly prolonged mouse survival over OxPt plus irinotecan without causing neutropenia, hepatotoxicity or nephrotoxicity. Our study uncovers a novel nanomedicine strategy to co‐deliver combination chemotherapies by actively targeting LDLR in tumors and presents a promising clinical strategy for the treatment of mCRC.

## 4 Experimental section

### Materials, Cell Lines, and Animals

All starting materials were purchased from Sigma‐Aldrich and Fisher (USA), unless otherwise noted, and used without further purification. 1,2‐dioleoyl‐snglycero‐3‐phosphate (DOPA), 1,2‐dioleyl‐sn‐glycero‐3‐phosphocholine (DOPC), cholesterol, and 1,2‐distearoyl‐sn‐glycero‐3‐phosphoethanolamine‐N‐[amino(polyethylene glycol)2000] (DSPE‐PEG_2k_) were purchased from Avanti Polar Lipids (USA).

Murine mammary carcinoma cells MC38 and CT26 were purchased from American Type Culture Collection (Rockville, MD, USA) and cultured in Dulbecco's Modified Eagle's Medium (DMEM) and RPMI‐1640 medium (Gibco, Grand Island, NY, USA), respectively, supplemented with 10% fetal bovine serum, 100 U mL^−1^ penicillin G sodium, and 100 g mL^−1^ streptomycin sulfate in a humidified atmosphere containing 5% CO_2_ at 37 °C. Human colon cancer cells HT29, HCT116, and SW480 were purchased from American Type Culture Collection (Rockville, MD, USA) and cultured in McCoy's 5A (for HT29) or DMEM (for HCT116 and SW480), supplemented with 10% fetal bovine serum, 100 U mL^−1^ penicillin G sodium, and 100 g mL^−1^ streptomycin sulfate in a humidified atmosphere containing 5% CO_2_ at 37 °C.

BALB/c female mice (6 weeks, 18–22 g), C57BL/6 female mice (6 weeks, 18–22g), athymic female nude mice (6 weeks, 18−22 g), and SD/CD female rats (6 weeks, 160–200 g) were provided by Harlan Laboratories, Inc (USA). The study protocols (#72 334 and #72 408) were reviewed and approved by the Institutional Animal Care and Use Committee (IACUC) at the University of Chicago (PHS Assurance #D16‐00322 (A3523‐01)).

### Preparation and Characterization of OxPt/SN38

OxPt‐bare was synthesized according to the previously reported method with minor modifications.^[^ ^13c^ ^]^ Briefly, an aqueous solution of OxPt‐bp (30 mg, 150 mg mL^−1^) was added to a 5 mL of 0.3m Triton X‐100/1.5m 1‐hexanol in cyclohexane and stirred vigorously for 15 min in the presence of DOPA (30 mg, 200 mg mL^−1^ in CHCl_3_). An aqueous solution of Zn(NO_3_)_2_ (60 mg, 600 mg mL^−1^) was added to a 5 mL of 0.3m Triton X‐100/1.5m 1‐hexanol in cyclohexane and stirred vigorously for 5 min. The Zn(NO_3_)_2_‐containing microemulsion was added dropwise to the Pt‐containing microemulsion and stirred vigorously for 30 min at room temperature. After the addition of 10 mL ethanol, OxPt‐bare was obtained by centrifugation at 11 628 × g. The resulting pellet was washed twice with THF/ethanol and finally re‐dispersed in THF. OxPt loadings in the particles were determined by ICP‐MS (Agilent 7700 ×, Agilent Technologies, USA) after digestion with nitric acid.

OxPt/SN38 was prepared by adding a THF solution (80 µL) of DOPC, cholesterol, DSPE‐PEG_2k_, Chol‐SN38(3:3:1.5:1), and OxPt‐bare to 500 µL of 30% (v/v) ethanol/water at room temperature. The mixture was stirred at 1700 rpm for 1 min. THF and ethanol were completely evaporated and the solution was allowed to cool down to room temperature. The particle size and zeta potential were determined by DLS using a Zetasizer (Nano ZS, Malvern, UK). TEM (Tecnai Spirit, FEI, USA) was used to determine the morphology. OxPt/SN38 was mixed with saturated NaCl solution and 1% Triton X‐100 solution, followed by ethyl acetate extraction. The mixture was vortexed and centrifuged at 10 000 × g for 5 min, the supernatant was analyzed by LC‐MS (Agilent 6540, Agilent Technologies, USA) to determine Chol‐SN38 loading.

### Preparation and Characterization of Control NCP Particles

ZnP Bare or OxPt‐bare particles were synthesized according to the previously reported method with minor modifications.^[^ ^13c^ ^]^ Briefly, an aqueous solution of OxPt‐bp or sodium pyrophosphate (30 mg, 150 mg mL^−1^) was added to a 5 mL of 0.3m Triton X‐100/1.5m 1‐hexanol in cyclohexane and stirred vigorously for 15 min in the presence of DOPA (30 mg, 200 mg mL^−1^ in CHCl_3_). An aqueous solution of Zn(NO_3_)_2_ (60 mg, 600 mg mL^−1^) was added to a 5 mL of 0.3m Triton X‐100/1.5m 1‐hexanol in cyclohexane and stirred vigorously for 5 min. The Zn(NO_3_)_2_‐containing microemulsion was added dropwise to the Pt‐containing microemulsion and stirred vigorously for 30 min at room temperature. After the addition of 10 mL ethanol, bare particles were obtained by centrifugation at 11 628 × g. The resulting pellet was washed twice with THF/ethanol and finally re‐dispersed in THF.

NCPs were prepared by adding a THF solution (80 µL) of DOPC, cholesterol, DSPE‐PEG_2k_, Chol‐drugs (Chol‐SN38 or Chol‐pyropheophytin a) (3:3:1.5:1), and bare particles to 500 µL of 30% (v/v) ethanol/water at room temperature. The mixture was stirred at 1700 rpm for 1 min. THF and ethanol were completely evaporated and the solution was allowed to cool down to room temperature. The particle size and zeta potential were determined by DLS using a Zetasizer (Nano ZS, Malvern, UK). TEM (Tecnai Spirit, FEI, USA) was used to observe the morphology.

### Stability of Chol‐SN38 on OxPt/SN38 Particles

2 mL of OxPt/SN38 particles with a Chol‐SN38 concentration of 20 ppm was incubated in PBS at 37 °C. At 0, 1, 3, 5, 8, 24, 48, and 72h, 20 µL aliquot from the solution was harvested, mixed with 100 µL of 0.5%Triton X‐100 saturated NaCl solution and 100 µL of ethyl acetate, vortexed for 1 min, and then centrifuged for 5 min at 14 000 rpm. The organic fraction was collected and analyzed by LC‐MS.

### Isothermal Titration Calorimetry Analysis

ITC experiments were performed with an ITC200 calorimeter (Microcal Malvern) at 37 °C. The lipoprotein or albumin concentration (in PBS) in the microcalorimeter cell and the concentrations of Chol‐SN38 (PBS with 3% DMSO) or each of the nanoparticles (based on Chol‐SN38 or corresponding components in PBS) in the syringe were adjusted to 15 and 200 µm, respectively. A first injection of 0.4 µL was followed by 20 injections of 2 µL at intervals of 150 s. The data were analyzed according to the one binding‐site model using the MicroCal Origin software provided by the manufacturer (OriginLab Corporation, Northampton, MA 01060).

### In Silico MD Simulations

MD simulations were carried out according to previously published procedures.^[^ ^18^ ^]^ A slice of spherical LDL particle with 10% of its full volume was used to reduce the system size and make it computationally tractable on the atomic level. The lipid composition in Table S2, Supporting Information, corresponds to typical human LDL particles and the first coarse‐grained LDL model.^[^ ^26^ ^]^ The system was simulated with periodic boundary conditions as in the simulation set up of lipid bilayer. ApoB protein was not included in the simulation because the hydrophobic core provides the main interactions between LDL and Chol‐SN38. The lipid force field was used as it was considered one of the best all‐atom force fields for lipid systems.

For MD simulations, the LDL‐like slice was designed as follows. Topologies for lyso PC, cholesterol oleate, and glycerol oleate were acquired from Automated Topology Builder (ATB) and Repository^[^ ^27^ ^]^ and modified manually by combining pieces of POPC and cholesterol. Initial topology of SN38 and Chol‐SN38 were generated by Acpype topology generator.^[^ ^28^ ^]^ The structure of SN38 was optimized by gaussian09 at the B3LYP/6‐31++G(d) level of theory and ESP partial charges were added to the initial topology. The cholesterol tail of Chol‐SN38 was adjusted to match the lipid force field. First, the hydrophobic core of a random mixture of cholesterol, cholesterol oleate, and glycerol trioleate was generated by packmol^[^ ^29^ ^]^ and lyso PC and POPC were added to each side of the core. The system was then solvated on both sides and equilibrated for 200 ns with a time step of 2 fs.

Gromacs 5.1.4 package^[^ ^30^ ^]^ was used to implement all MD simulations. TIP3P water model was used. All simulations were performed in NPT conditions with a constant pressure of 1 bar and temperature of 320 K maintained by v‐rescale thermostat and Berendsen barostat, respectively. Potentials of mean force (PMFs) of transporting SN38 and Chol‐SN38 to the center of LDL slice were computed by umbrella sampling simulations. The center masses of SN38 and Chol‐SN38 were restrained by harmonic potential characterized by a 2000 kJ mol^−1^ nm^−2^ force constant at different distances from the center of the slice. Nine sampling windows were selected with a step interval of 1 nm along Z axis. Each window was sampled for 200 ns and the last 100 ns were used for statistical analysis. PMFs were obtained by the weighted histogram technique^[^ ^31^ ^]^ in the Gromacs package.

### Lipoprotein Binding Studies

20 ppm of SN38, Chol‐SN38, and OxPt/SN38 (based on Chol‐SN38) were prepared in fresh rat plasma. After incubation at 37 °C for 0.5, 1, 3, or 5 h, 20 uL of plasma was pipetted and added to 20 uL of 2 × LDL Precipitation Buffer (BioVision. Incorporated). 10 min later, the mixture was centrifuged at 5000 rpm for another 10 min. The supernatant was analyzed by LC‐MS to determine the drug concentrations remaining in the solution (unbound to LDL).

### ApoB‐100 Binding Studies

100 µL of 0.5 mg mL^−1^ ApoB‐100 was added to 50 µL of 0, 25, 50, 100, or 200 mg mL^−1^ of ZnP NCP or ZnP NCP without cholesterol and incubated at 37 °C for 3h. Unbound Apo B‐100 was precipitated by 0.01 m acetic acid and immediately centrifuged at 4000 rpm for 5 min. During this process, NCP particles remained in the solution. The amount of ApoB‐100 in the supernatant was quantified by BCA assay. ZnP NCP was used here to avoid interference with the BCA absorption signals at 562 nm.

5 µL of NCP was mixed with 95 µL mouse plasma for 3 h. 50 µL of the plasma solutions were added with 0.01 m acetic acid for precipitating the major unbound proteins. The samples were centrifuged at 4000 rpm for 5 min, and then the supernatant was analyzed by western blot.

### Drug Distributions in Rat Plasma

The distribution of drugs in different plasma fractions was studied by ultracentrifugation of lipoproteins on the basis of their hydrated densities as previously described by Cassidy et al.^[^ ^32^ ^]^ In brief, 300 µL rat plasma was slowly and gently added to the top of 150 µL of 1.006 g mL^−1^ NaBr solution, and then centrifuged in an Optima Max‐XP Tabletop Ultracentrifuge (Beckman Instruments, Inc.) with a TLA120.1 fixed angle rotor (Beckman Instruments, Inc.) at 420 000 g at 4 °C for 1.5 h. After the top layer of 150 µL VLDL fraction was removed, 150 µL of 1.182 g mL^−1^ NaBr solution was mixed with the remaining solution to separate the LDL fraction (1.006 g mL^−1^ < *ρ* < 1.063 g mL^−1^) at 420 000 g at 4 °C for 2.5 h. After the top layer of 150 µL LDL fraction was removed, 150 µL of 1.478 g mL^−1^ NaBr solution was mixed with the remaining solution to separate the HDL fraction (1.063 g mL^−1^ < *ρ* < 1.21 g mL^−1^), and the remaining albumin fraction was centrifuged at 420 000 g at 4 °C for 4 h. Lipid‐staining Sudan black (0.01% w/v) was added to the 1.25 g mL^−1^ solution (one tube) to visualize all lipoprotein fractions.

### In Vivo Drug Distribution in Plasma

SD/CD rats were i.v. injected with OxPt/SN38 at a dose of 9.13 mg kg^−1^ Chol‐SN38 and at 0.5, 1, 3, 5, 8, or 24 h post‐injection, rat plasma was harvested for lipoprotein separation by ultracentrifugation. 20 µL of each fraction was analyzed by LC‐MS.

### In Vitro Cellular Uptake

MC38 cells seeded in six‐well plates (5 × 10^4^ cells/well) were starved by removing serum‐containing medium and incubating in FBS‐free DMEM for 24h. 100 ug of Chol‐pyro NCP or Ce6 NCP (core labeled) was i.v. injected into the tail veins of C57BL/6 mice. Three hours later, the plasma was obtained and added to the serum‐deprived cells, which were incubated with PBS in the presence of 0, 1, or 10 µg mL^−1^ LDLR antibody for 2 h. 24 h later, the cells were harvested and analyzed by flow cytometry or confocal imaging. Cell uptake of Dil‐LDL was similarly studied.

LDLR KO MC38 cells were obtained following the protocol of LDLR CRISPR Plasmids (m) (Santa Cruz Biotechnology). WT or LDLR KO MC38 cells seeded in six‐well plates (5 × 10^4^ cells/well) were serum starved by removing the culture medium and incubating in FBS‐free DMEM for 24h. One hundred ug of Chol‐pyro NCP or Ce6 NCP was i.v. injected into C57BL/6 mice. Three hours later, the plasma was obtained and added into serum‐deprived cells. 24 h later, cells were harvested and analyzed by flow cytometry.

MC38 cells seeded in six‐well plates (5 × 10^4^ cells/well) were starved by removing the culture medium and incubating in FBS‐free DMEM for 24h. 100 ug of Chol‐pyro NCP was injected into C57Bl/6 mice. Three hours later, the plasma was obtained and added into serum‐deprived cells, which were incubated with 10 µg mL^−1^ IgG or an anti‐LDLR antibody (*α*‐LDLR) for 2 h. The cells were then stained with LysoTracker Red DND‐99 (50 nm) for 1 h. The cell medium was replaced with fresh medium and cells were incubated for another 30 min, and then fixed with 4% paraformaldehyde for 10 min, stained with DAPI for 5 min, and imaged with CLSM (Leica SP8).

### LDLR Expression on Tumor Cells

MC38, MC38 LDLR KO, CT26, HT29, HCT116, or SW480 cells were seeded in six‐well plates (5 × 10^4^ cells/well). 24 h later, the cells were harvested and stained with primary anti‐LDLR antibody (1:100) for 1 h on ice. The cells were washed by PBS twice and stained with AF488 secondary anti‐rabbit antibody (1:1000) on ice for 30 min. The cells were washed with PBS and analyzed by flow cytometry.

### In Vivo Tumor Uptake

C57BL/6 mice were subcutaneously injected with 2 × 10^6^ MC38 cells in the right flanks. When the tumors reached ≈100 mm^3^, mice were i.v. administrated PBS or 100 µg Chol‐pyro NCP. 24 and 48 h later, tumors were harvested. Frozen 5 µm tissue sections were prepared using a cryostat. The sections were fixed in acetone for 10 min at −20 °C and stained with DAPI for another 10 min. The sections were then washed twice with PBS and imaged with CLSM.

The freshly harvested tissues were treated with 1 mg mL^−1^ collagenase I (Gibco, USA) for 1 h at 37 °C, and ground with the rubber end of a syringe. Cells were filtered through nylon mesh filters and washed with PBS. The single‐cell suspensions were analyzed by flow cytometry.

### In Vivo Biodistribution Analysis

C57Bl/6 mice were subcutaneously injected in the right flank with 1 × 10^6^ MC38 cells. When the tumors reached ≈100 mm^3^, mice were intravenously (i.v.) administrated free OxPt or OxPt/SN38 at a dose of 3.5 mg kg^−1^ OxPt and 6.2 mg kg^−1^ SN38 equivalent (OxPt:SN38 = 1: 1.8). The livers, lungs, spleens, kidneys, and tumors were collected at 1, 3, 8, 24, 48, and 72 h post‐injection. The content of Pt was quantified by ICP‐MS.

### Release of OxPt and SN38 from OxPt/SN38

100 ppm of OxPt/SN38 solution was prepared in pH = 7.4 or pH = 4.7 aqueous conditions. At different time points, 200 µL aliquots were centrifuged with Amicon Ultra‐0.5 Centrifugal Filter Unit (NMWL: 30 Kda) at 14 000 × g for 10 min. The filtrates were analyzed by ICP‐MS to determine the Pt contents. For the extraction of SN38, 100 µL aliquots were added to 100 µL of saturated NaCl solution, 5 µL of 20% Triton X‐100 aqueous solution, and 100 µL ethyl acetate. The mixture was vigorously vortexed for 30 s and then centrifuged at 14 000 × g for 5 min. The upper organic layer was analyzed by LC‐MS to determine SN38 concentrations.

### Apoptosis/Necrosis Analysis

MC38 cells seeded in six‐well plates (5 × 10^4^ cells/well) were treated with OxPt, SN38, OxPt plus SN38, OxPt NCP, ZnP/SN38, or OxPt/SN38 at 5 µm OxPt or/and 5 µm SN38 for 24 h. The cells were harvested, washed twice with ice‐cold PBS, stained with Alexa Fluor 488‐Annexin V and propidium iodide (PI) for 15 min at room temperature in the dark, and then analyzed by flow cytometry (LSR II, BD, USA).

### In Vitro Cytotoxicity

Colon cancer cells were seeded in 96‐well plates at a density of 2 × 10^3^ cells per well and allowed to adhere for 24 h. Cells were then treated with different concentrations of OxPt, SN38, irinotecan, Chol‐SN38, SN38‐TMS, OxPt NCP, ZnP/SN38, or OxPt/SN38 for 48 h. Cell viability was determined by 3‐(4,5‐dimethylthiazol‐2‐yl)‐5‐(3‐carboxymethoxyphenyl)‐2‐(4‐sulfophenyl)‐2H‐tetrazolium assay (Promega, Madison, WI) according to the manufacturer's instructions.

### Cell Cycle Assay

MC38 cells seeded in six‐well plates (5 × 10^4^ cells/well) were treated with OxPt, SN38, OxPt NCP, ZnP/SN38, or OxPt/SN38 at 5 µm OxPt and/or 5 µm SN38 for 24 h. The cells were harvested, washed twice with ice‐cold PBS, fixed with 70% ethanol at 4 °C overnight, treated with Rnase A for 45 min, and stained by PI for 30 min. Cell cycle distributions (Go, G1, S, and G2M) were analyzed by flow cytometry.

### Depolarization of Mitochondrial Membrane Potential

MC38 cells seeded in six‐well plates (5 × 10^4^ cells/well) were treated with OxPt, SN38, OxPt plus SN38, OxPt NCP, ZnP/SN38, or OxPt/SN38 at 5 µm OxPt and/or 5 µm SN38 for 24 h. The cells were stained with JC‐1 (Abcam, UK) at a concentration of 10 µm at 37 °C for 30 min in the dark, harvested, washed twice with ice‐cold PBS, and analyzed by flow cytometry.

### Cytochrome c Release

After treatment with OxPt, SN38, OxPt plus SN38, OxPt NCP, ZnP/SN38, or OxPt/SN38 at 5 µm OxPt and/or 5 µm SN38 for 24 h, MC38 cells were sequentially stained with MitoTracker Red CMXRos (100 µm) for 15 min, fixed with 4% paraformaldehyde for 10 min, permeabilized with 0.2% Triton X‐100 for 10 min, incubated with anti‐cytochrome c (eBioscience, diluted 1:100) for 2 h, stained with DAPI for 10 min, and imaged with CLSM (Leica SP8, Germany).

### In Vivo Anticancer Efficacy

2 × 10^6^ cells CT26 or MC38 cells were subcutaneously injected into the right flank regions of 6‐week‐old BALB/c or C57BL/6 mice, respectively. Seven days after cell injection, PBS, OxPt plus irinotecan, OxPt NCP, ZnP/SN38, and OxPt/SN38 were i.v. injected once every 3 days (Q3D) at doses of 3.5 mg kg^−1^ OxPt or equivalent, 20.2 mg kg^−1^ irinotecan (11.7 mg kg^−1^ SN38 equivalent), and/or 15.9 mg Chol‐SN38/kg (6.2 mg kg^−1^ SN38 equivalent) to MC38 model for up to 8 doses and to CT26 model for up to 6 doses, n = 6.

5 × 10^6^ cells HT29, HCT116, or SW480 were subcutaneously injected into the right flank regions of 6‐week‐old athymic nude mice, respectively. Seven days after cell injection, PBS, OxPt plus irinotecan, OxPt NCP, ZnP/SN38, and OxPt/SN38 were i.v. injected once every 3 days (Q3D) at doses of 3.5 mg kg^−1^ OxPt or equivalent, 20.2 mg kg^−1^ irinotecan (11.7 mg kg^−1^ SN38 equivalent), and/or 15.9 mg Chol‐SN38/kg (6.2 mg kg^−1^ SN38 equivalent) to each model for up to 16 doses, n = 6. Tumor growth was monitored by measurement with a digital caliper, with tumor volumes calculated as (width^2^ × length)/2. TGI is defined as 1‐(RTVt/RTVc) where RTV = endpoint tumor volume).

### In Vivo Pharmacokinetics and Biodistribution Analysis

SD/CD rats were intravenously (i.v.) injected with OxPt/SN38 at an equivalent OxPt dose of 2 mg kg^−1^. The blood was collected at 5 min, 30 min, 1 h, 3 h, 5 h, 8 h, 24 h, or 48 h post‐injection and immediately centrifuged at 604 × g for 10 min to harvest plasma samples. 20 µL plasma was digested with concentrated nitric acid for 24 h and analyzed for Pt concentration by ICP‐MS. Another 20 µL plasma was added to 5 µL 20% Triton X‐100 to disrupt the lipid bilayer of the nanoparticles. Chol‐SN38 and its metabolites were extracted from plasma by adding 100 µL ethyl acetate, followed by centrifugation at 6708 × g for 10 min. Chol‐SN38 and its metabolites were quantified by LC‐MS.

C57BL/6 mice were subcutaneously injected with 1 × 10^6^ MC38 cells into the right flanks. When the tumors reached ≈100 mm^3^, mice were i.v. administrated OxPt plus irinotecan or OxPt/SN38 at doses equivalent to 3.5 mg kg^−1^ OxPt and 6.2 mg kg^−1^ SN38. The livers, lungs, spleens, kidneys, and tumors were collected at 1, 3, 8, 24, 48, or 72 h post injection. Pt concentrations were quantified by ICP‐MS while Chol‐SN38 and SN38 concentrations were quantified by LC‐MS.

### Liver and Kidney Toxicity Evaluation

MC38 tumor‐bearing C57BL/6c mice (female, n = 6) were randomly assigned and i.v. injected with OxPt/SN38 once every 3 days for up to 15 doses. On day 22 (after 8 doses) and day 43 (after 15 doses), blood was harvested to measure AST, ALT, and serum creatinine (sCr) with AST Activity Assay Kit, ALT Activity Assay Kit, and Creatinine Assay Kit (Sigma‐Aldrich, USA), respectively, using a microplate reader.

### Absolute Neutrophil Counts

Blood samples were collected from MC38 tumor‐bearing C57BL/6 mice following 8 intravenous injections of PBS or OxPt/SN38 or 3 intravenous injections of OxPt plus irinotecan. Whole blood samples were added to BD Trucount tubes and stained with CD45, CD11b, and Gr‐1 antibodies for flow cytometry. Neutrophils were gated as SSChi by FSC/SSC on the CD45+ population and were confirmed to be CD11b+ and Gr‐1+.

### Hematoxylin and Eosin (H&E) Staining

Healthy C57BL/6 mice (Female, n = 6) were randomly assigned and treated with 8 total doses of OxPt/SN38 or OxPt and irinotecan given every three days. Mice were euthanized on day 22 after the first dose, and gross necropsies were performed. The tissues of interest were collected, fixed with 4% paraformaldehyde, embedded in paraffin, and cut into sections for hematoxylin and eosin (H&E) staining before undergoing histopathological examination with a Panoramic MIDI II Digital Slide Scanner.

### Statistical Analysis

The data of experiments including LDL binding studies, in vitro studies, and in vivo tumor accumulation were expressed as mean ± standard deviation (SD). The above experimental data were repeated three times in parallel (n = 3) and analyzed by one‐way ANOVA with Tukey's multiple comparison test. Sample sizes (n ≥ 5) were selected to ensure adequate statistical power with ANOVA for efficacy studies. Student's t‐tests were used to determine if the variance between groups was similar or between only two groups. Statistical analysis was performed using Prism software (version 7.0, GraphPad Software). *P < 0.05, **P < 0.01, ***P < 0.001. Animal experiments were represented as mean ± SD.

## Conflict of Interest

W.L. is the founder and chairman of Coordination Pharmaceuticals, which licensed the NCP technology from the University of Chicago. R.R.W. is an advisor to Coordination Pharmaceuticals.

## Author Contributions

X.J. and W.L. conceived the project. X.J., W.H., J.L., J.M., M.J.L., and K.Y. performed the experiments and analyzed the results. M.R. and M.J.L. proofread the writing. Y.L. helped with Chol‐SN38 synthesis. T.L. and Z.X. helped with NCP characterization. X.J., M.B., R.R.W., and W.L. wrote the manuscript.

## Supporting information

Supporting Information

## Data Availability

The data that support the findings of this study are available from the corresponding author upon reasonable request.
