# Correction to “Tumor‐Activatable Nanoparticles Target Low‐Density Lipoprotein Receptor to Enhance Drug Delivery and Antitumor Efficacy”

**DOI:** 10.1002/advs.202412651

**Published:** 2024-11-04

X. Jiang, W. Han, J. Liu, J. Mao, M. J. Lee, M. Rodriguez, Y. Li, T. Luo, Z. Xu, K. Yang, M. Bissonnette, R. R. Weichselbaum, W. Lin, *Adv. Sci*. **2022**, *9*, e2201614.

https://doi.org/10.1002/advs.202201614

Transmission electron micrograph (TEM) images at different magnifications were mistakenly included in Figure S6a on Page S12 of the Supporting Information (i.e., TEM images for ZnP/SN38 and OxPt NCP w/o chol are identical images at different magnifications and TEM images for ZnP/SN38 and ZnP NCP are identical images at different magnifications). An incorrect TEM image was also used in Figure 1e.

The correct images are shown below:


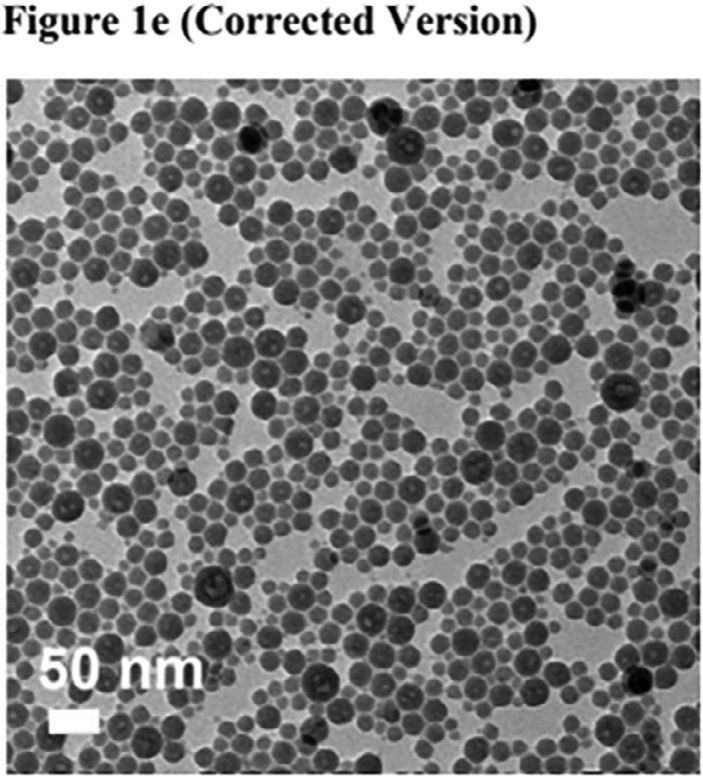



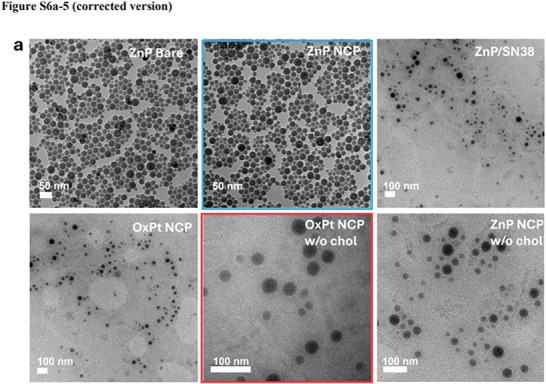


We have carefully reexamined all figures in the main document and Supporting Information, and we are confident that these corrections do not impact the conclusions of our paper. We apologize for this error.

