# Supplementary material for: Tumor‐Activatable Nanoparticles Target Low‐Density Lipoprotein Receptor to Enhance Drug Delivery and Antitumor Efficacy
Source: Adv Sci (Weinh). 2022 Jun 24;9(24):2201614. doi: 10.1002/advs.202201614 (PMC9404402; doi:10.1002/advs.202201614)
Supplement: Supplementary file 1 — Supporting Information [file ADVS-9-2201614-s001.pdf]

ADVANCED SCIENCE
Open Access

Supporting Information

for *Adv. Sci.*, DOI 10.1002/advs.202201614

Tumor-Activatable Nanoparticles Target Low-Density Lipoprotein Receptor to Enhance Drug Delivery and Antitumor Efficacy

*Xiaomin Jiang, Wenbo Han, Jianqiao Liu, Jianming Mao, Morten J. Lee, Megan Rodriguez, Youyou Li, Taokun Luo, Ziwan Xu, Kaiting Yang, Marc Bissonnette, Ralph R. Weichselbaum and Wenbin Lin**

Supporting Information for

Tumor-Activatable Nanoparticles Target Low-Density Lipoprotein Receptor to Enhance Drug Delivery and Antitumor Efficacy

Xiaomin Jiang,[1] Wenbo Han,[1] Jianqiao Liu,[1] Jianming Mao,[1] Morten J. Lee,[1] Megan Rodriguez,[1] Youyou Li,[1] Taokun Luo,[1] Ziwan Xu,[1] Kaiting Yang,[3] Marc Bissonnette,[2] Ralph R. Weichselbaum,[3] Wenbin Lin[1,3,*]

[1]Department of Chemistry, The University of Chicago, 929 E 57th St, Chicago, IL 60637, USA.

[2]Department of Medicine, Division of Biological Sciences, Chicago, IL 60637, USA.

[3]Department of Radiation and Cellular Oncology and Ludwig Center for Metastasis Research, The University of Chicago, 5758, S Maryland Ave, Chicago, IL 60637, USA.

[*]Corresponding author. E-mail: wenbinlin@uchicago.edu

Supplementary methods

Synthesis and characterization of cholesterol-conjugated SN38 prodrug (Chol-SN38) (Figure S1)

Synthesis of chloromethyl 4-nitrophenyl carbonate

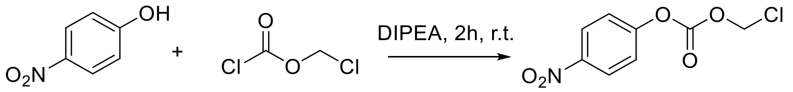

10 g (72 mmol) 4-nitrophenol was dissolved in 200 mL anhydrous DCM in a 500 mL round bottom flask with nitrogen protection. 20 mL (115 mmol, 1.6 eq) DIPEA was then added to the flask and the flask was cooled in an ice bath. 10 mL (14.5 g, 115 mmol, 1.6 eq) chloromethyl chloroformate was added dropwise to the solution. The solution was then stirred at room temperature for 2 h. The yellowish color disappeared, and the final solution was deep red-pink after 2 h reaction. The solution was washed with 200 mL water twice, 200 mL 1M HCl once, another 200 mL water once, and finally 200 mL saturated NaCl solution once. The organic phase was dried over anhydrous Na_2SO_4 for 2 h. The solution was concentrated on a rotary evaporator to afford a deep red oil as the crude product.

600 mL of hexane/isopropyl ether (10:1 V/V) was added to the crude product and heated to boil to obtain a light-yellow solution and a deep red precipitate. The solution was transferred to another flask and cooled in a -20 °C freezer overnight. Colorless to pale-yellow needle-shaped crystals formed in the freezer were collected. The hexane/isopropyl ether solution was

concentrated to obtain additional product. Yield: 14.5 g (62.3 mmol, 87%). ^{1}H-NMR (500MHz, CDCl3): δ= 5.85 (s, 2H), 7.42 (d, *J*=8Hz, 2H), 8.30 (d, *J*=9Hz, 2H).

Synthesis of cholesteryl hydrogen succinate

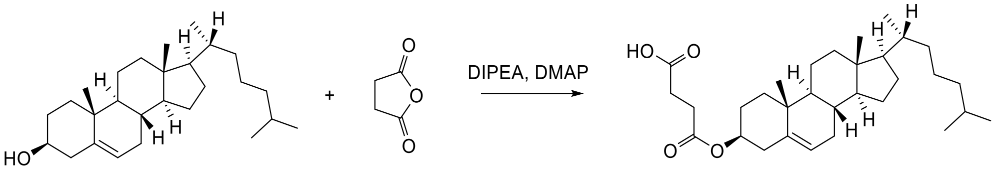

8 g (20 mmol) cholesterol, 6 g succinic anhydride (60 mmol, 3 eq), 400 mg DMAP (3.25 mmol), and 6 mL DIPEA (35 mmol, 1.75 eq.) were mixed in 250 mL anhydrous THF and refluxed under nitrogen protection for 48 h to obtain a deep yellow/red solution. The solvents were evaporated on a rotary evaporator to obtain a white-brown solid as the crude product.

The crude product was transferred to a 500 mL flask using 100 mL THF. 150 mL saturated $NaHCO_3$ solution was added to the flask and the mixture was stirred for 3 h until no more bubble was generated. Then the mixture was neutralized with 1M HCl solution until pH < 5 to obtain a light brown liquid-solid mixture. The mixture was extracted with 200 mL EtOAc three times and the combined organic phase was washed with 200 mL 1M HCl solution twice and 200 mL saturated NaCl solution once. The organic phase was dried over anhydrous Na_2SO_4 for 2 h and evaporated to obtain a light brown solid. The solid was dissolved in 100 mL boiling EtOAc and cooled in a -20 °C freezer overnight. White crystals formed and were collected as the pure product. Yield: 9.8 g (20 mmol, 100%). ^{1}H-NMR (500 MHz, $CDCl_3$): δ=0.5–2.35 (m, 43 H), 2.62 (t, 2H), 2.69 (t, 2H), 4.63 (m, 1H), 5.38 (d, 1H, *J*=4.4 Hz). HRMS: m/z=504.4040 (expected 504.4053 for $[M+NH_4]^+$).

Synthesis of iodomethyl 4-nitrophenyl carbonate

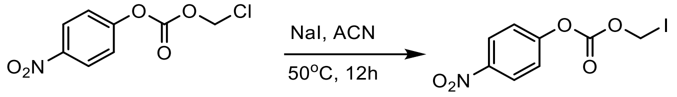

7 g (30 mmol) chloromethyl 4-nitrophenyl carbonate and 14 g (90 mmol, 3 eq) NaI was dissolved in 300 mL anhydrous acetonitrile and stirred at 50 °C under nitrogen protection for 24 h. The solution turned light yellow and white precipitates formed. The solvent was removed by rotary evaporation and the solid was further dried on vacuum for 2 h. 300 mL DCM was added to the dried solid to extract the product. The DCM solution was filtered and concentrated to obtain a light-yellow oil or solid as the crude product which was used the subsequent reaction without further purification.

Synthesis of 20-O-trimethylsilyl-SN38 (Figure S2)

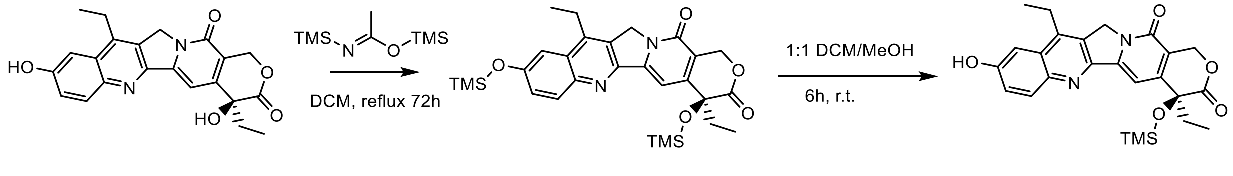

A mixture of 5 g (12.7 mmol) SN38 and 25 mL N, O-bis(trimethylsilyl) acetamide in 25 mL anhydrous DCM was refluxed under nitrogen protection for 72 h. SN38 gradually dissolved to form a deep red solution. The reaction was monitored by TLC (3:1 DCM : EtOAc, visible under UV light) to ensure complete conversion of SN38 to 10, 20-O-bis(trimethylsilyl)-SN38 (TMS_2-SN38). The solvent was removed by rotary evaporation and the residue was further dried under vacuum at 50 °C to remove acetamide. The brown solid of TMS_2-SN38 was dissolved in 100 mL DCM and 100 mL methanol and stirred at room temperature for 6 h. The reaction was monitored by TLC to ensure complete conversion of TMS_2-SN38 to 20-O-TMS-SN38. Then the solution was dried to obtain a deep yellow/brown solid as the crude product which was further dried under vacuum and then directly used in the subsequent reaction without further purification. ^{1}H NMR (500 MHz, Chloroform-*d*) δ 9.74 (s, 1H), 8.13 (d, *J* = 9.1 Hz, 1H), 7.57 (s, 1H), 7.51 (dd, *J* = 9.1, 2.6 Hz, 1H), 7.47 (d, *J* = 2.6 Hz, 1H), 5.70 (d, *J* = 16.4 Hz, 1H), 5.28 (d, *J* = 16.3 Hz, 1H), 5.23 (s, 2H), 3.05 (d, *J* = 7.6 Hz, 2H), 1.85 (dd, *J* = 7.3, 2.3 Hz, 2H), 1.32 (t, *J* = 7.6 Hz, 3H), 0.88 (t, *J* = 7.3 Hz, 3H), 0.21 (s, 12H). ^{13}C NMR (126 MHz, $CDCl_3$) δ 172.26, 157.87, 156.81, 152.37, 148.83, 146.71, 144.42, 143.87, 131.68, 129.00, 128.77, 126.95, 122.98, 117.92, 105.61, 98.53, 77.33, 77.07, 76.82, 75.90, 65.84, 49.57, 32.55, 23.20, 13.60, 7.78, 2.15, 1.91, 1.67. HRMS: m/z=465.1850 (expected 465.1767 for $[M+H]^+$).

Synthesis of Cholest-5-en-3-ol ((((4-nitrophenoxy)carbonyl)oxy)methyl) succinate (cholesterol linker) (Figure S3)

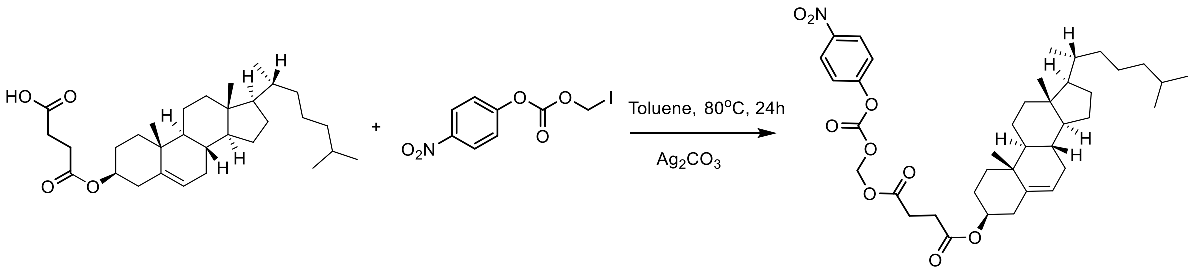

Crude iodomethyl 4-nitrophenyl carbonate from 7 g chloromethyl 4-nitrophenyl carbonate (30 mmol, 1.5 eq) was dissolved in 250 mL anhydrous toluene and then 9.8 g (20 mmol, 1eq) cholesteryl hydrogen succinate was added to the solution. The mixture was stirred with heating until all solids dissolved. 5.6 g (20 mmol, 1 eq) Ag_2CO_3 was then added to the solution and the solution was stirred in dark at 80 ^{o}C under nitrogen protection for 24 h. The resulting pale-yellow solution with black precipitates was filtered through celite and concentrated to obtain a light-yellow oil which was purified by column chromatography using silica with 30% hexanes in DCM (V/V) and then pure DCM as eluents. Yield: 7 g (10 mmol, 50%). ^{1}H NMR (500 MHz, Chloroform-*d*) δ 8.32 – 8.27 (m, 2H), 7.47 – 7.41 (m, 2H), 5.90 (s, 2H), 5.35 (dt, *J* = 5.3, 1.7 Hz, 1H), 4.66 – 4.57 (m, 1H), 2.75 (ddd, *J* = 7.3, 5.8, 1.2 Hz, 2H), 2.67 (ddd, *J* = 7.3, 6.0, 1.2 Hz, 2H), 2.32 (dd, *J* = 7.3, 1.8 Hz, 2H), 2.05 – 1.92 (m, 2H), 1.89 – 1.80 (m, 3H), 1.65 – 0.79 (m, 40H), 0.68 (s, 3H). ^{13}C NMR (126 MHz, $CDCl_3$) δ 171.27, 170.91, 155.08, 151.45, 145.64, 139.48, 125.39, 122.83, 121.78, 82.49, 77.34, 77.09, 76.83, 74.69, 56.66, 56.14, 50.01, 42.32, 39.72, 39.55, 38.03, 36.97, 36.59, 36.21, 35.82, 31.91, 31.86, 29.13, 28.99, 28.26, 28.04, 27.72, 24.31, 23.87, 22.87, 22.61, 21.05, 19.31, 18.75, 11.88. HRMS: m/z=699.4226 (expected 699.4221 for $[M+NH_4]^+$).

Synthesis of 7-ethyl-10-(((((cholest-5-en-3-oxy)-4-oxobutanoyl)oxy)methoxy)carbonyl)oxyl-20-O-trimethylsilyl camptothecin (chol-SN38) (Figure S4)

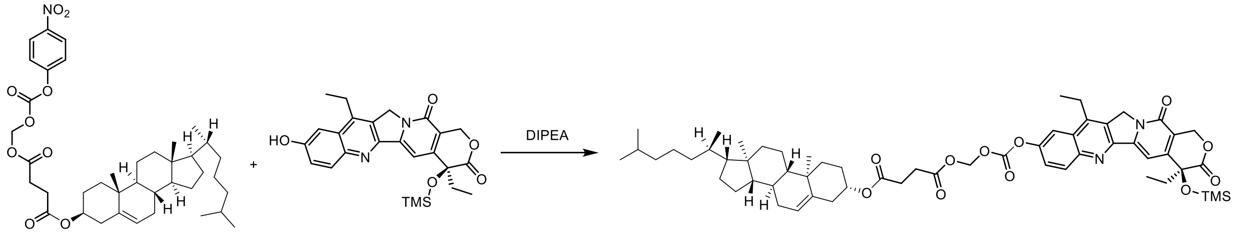

7 g (10 mmol) cholesterol linker and crude SN38-TMS from 5 g SN38 (12.7 mmol, 1.27 eq) was dissolved in 250 mL anhydrous DCM. 10 mL (60 mmol, 6 eq) DIPEA was then added and the resultant solution was stirred at room temperature under nitrogen protection for 24 h. The deep red solution was diluted with 500 mL DCM and washed with saturated $NaHCO_3$ aqueous solution three times, 1M HCl solution once, and then saturated NaCl solution once. The organic phase was dried over anhydrous Na_2SO_4 for 2 h and concentrated by rotary evaporation. The product was further purified by column chromatography on silica using 15:1 DCM: EtOAc (V/V) as eluent. Yield: 8 g (8 mmol, 80%). ^{1}H NMR (500 MHz, Chloroform-*d*) δ 8.28 (d, *J* = 9.2 Hz, 1H), 7.96 (d, *J* = 2.6 Hz, 1H), 7.68 (dd, *J* = 9.2, 2.6 Hz, 1H), 7.52 (s, 1H), 5.93 (s, 2H), 5.68 (d, *J* = 16.6 Hz, 1H), 5.34 – 5.19 (m, 5H), 4.61 (dt, *J* = 7.8, 2.2 Hz, 1H), 3.16 (d, *J* = 7.7 Hz, 2H), 2.79 – 2.73 (m, 2H), 2.70 – 2.63 (m, 2H), 2.30 (d, *J* = 7.7 Hz, 2H), 2.02 – 1.73 (m, 8H), 1.63 – 0.74 (m, 50H), 0.63 (s, 3H), 0.24 (s, 11H). ^{13}C NMR (126 MHz, $CDCl_3$) δ 171.92, 171.24, 170.93, 157.59, 152.34, 152.28, 151.76, 149.47, 147.58, 146.26, 145.47, 139.43, 132.45, 127.43, 127.35, 124.34, 122.74, 119.00, 114.08, 98.27, 82.44, 77.30, 77.05, 76.79, 75.82, 74.64, 65.93, 56.65, 56.16, 49.96, 49.32, 42.27, 39.71, 39.49, 38.00, 36.92, 36.53, 36.16, 35.79, 32.75, 31.84, 31.79, 29.70, 29.13, 29.01, 28.22, 28.01, 27.70, 24.26, 23.88, 23.21, 22.83, 22.57, 20.98, 19.28, 18.70, 14.02, 11.83, 7.89, 2.11, 1.87, 1.63. HRMS: m/z=1007.5472 (expected 1007.5375 for $[M+H]^+$).

Synthesis and characterization of fluorescently labelled cholesterol conjugate (Chol-pyropheophytin a) (Figure S10)

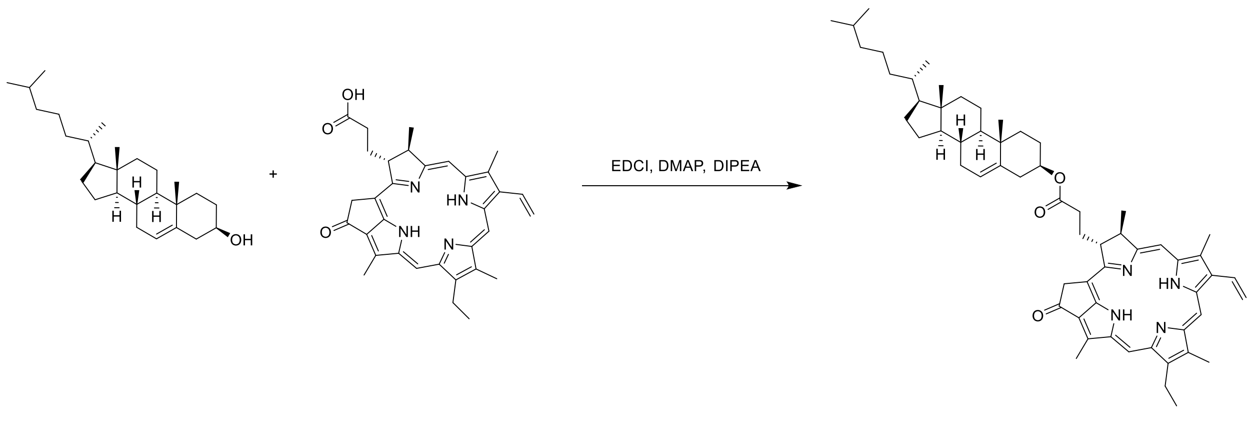

Pyropheophytin a was prepared as previously reported[10]. A mixture of pyropheophytin a (100 mg, 0.187 mmol), cholesterol (142 mg, 0.37 mmol), EDCI (72 mg, 0.37 mmol), DMAP (10 mg, 0.08 mmol) and DIPEA (100 µL) was stirred in anhydrous DCM (5 mL) at room temperature for 24 h. The mixture was loaded directly on a DCM-packed silica gel column and purified with a gradient elution from 5:1 DCM: EtOAc to 3:1 DCM: EtOAc to afford pure cholesterol- pyropheophytin a (Chol-pyro). Yield: 162 mg (60.4%). ^{1}H NMR (400 MHz, $CDCl_3$) δ 9.55 (s, 1H), 9.43 (s, 1H), 8.66 (s, 1H), 7.99 (dd, J = 17.8, 11.5 Hz, 1H), 6.35 – 6.12 (m, 2H), 5.34 (s, 2H), 5.30 – 4.99 (m, 3H), 4.54 (dd, J = 10.8, 5.7 Hz, 2H), 4.34 (d, J = 9.0 Hz, 1H), 3.68 (d, J = 6.9 Hz, 6H), 3.42 (s, 3H), 3.23 (s, 3H), 2.54 (s, 1H), 2.40 – 2.26 (m, 3H), 2.19 (tt, J = 11.4, 5.8 Hz, 3H), 1.85 (d, J = 7.2 Hz, 7H), 1.67 (q, J = 7.3 Hz, 12H), 1.59 – 0.77 (m, 59H), 0.62 (s, 3H). ESI-MS: m/z=903.6074 (expected 903.6148 for $[M+H]^+$).

Synthesis and characterization of 20-O-tert-butyl-SN38 (SN38-^{t}Bu) and Synthesis of 20-O-Boc-SN38 (SN38-Boc) (Figure S15-16)

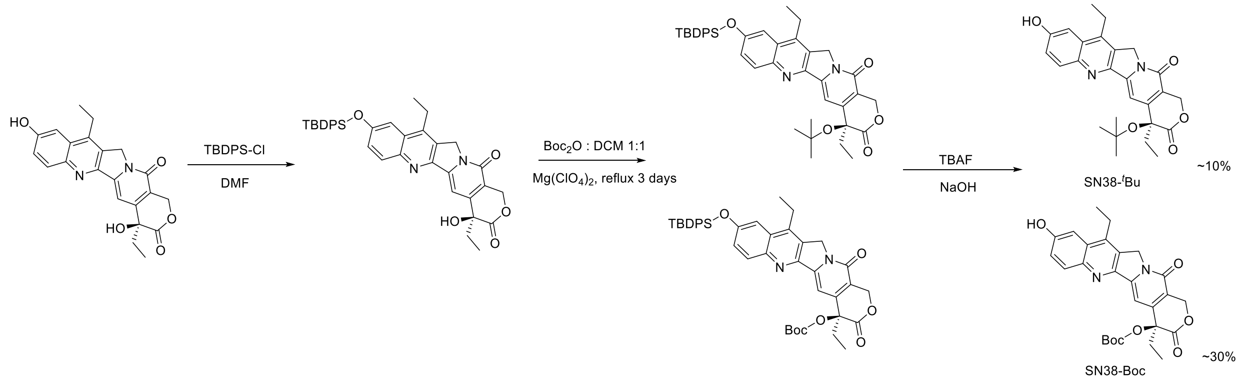

A 100 mL round bottom flask equipped with a stir bar, a rubber septum, and an argon inlet was charged with SN38 (100 mg, 0.255 mmol, 1.0 equiv.), N,N-dimethylformamide (35 mL), and imidazole (35 mg, 0.510 mmol, 2.0 equiv). The mixture was cooled at 0 °C while tert-butyl(chloro)diphenyl silane (TBDPS-Cl, 78 µL, 0.3 mmol, 1.1 equiv.) was added dropwise via a syringe. The reaction mixture was allowed to warm to room temperature (r.t.) and stirred at r.t. for 18 h. The reaction mixture was diluted with 100 mL of Et_2O and 15 mL H_2O and 15 mL 10% aq $KHSO_4$. The organic phase was separated and washed with 30 mL 5% aq $KHSO_4$ solution three times and 30 mL sat. aq. NaCl solution once. The organic phase was dried over Na_2SO_4, filtered, and concentrated to afford a yellow oil. Purification via column chromatography on silica gel (gradient elution with 0-40% EtOAc/hexanes) afforded 95 mg of TBDPS-SN38 yellow oil.

In a two-necked flask equipped with a magnetic stirring bar and a reflux condenser, $Mg(ClO_4)_2$ (0.01 mmol) and TBDPS-SN38 (0.1 mmol) were dissolved in 10 mL of CH_2Cl_2 and Boc_2O. The mixture was stirred at reflux for three days. The mixture of products was further purified by HPLC (gradient elution with 10-100% Methanol) and analyzed each eluent with UV signal in 370 nm by HRMS. To a solution of TBDPS-SN38 (0.2 mmol) in 5 mL DMF under argon was added NaOH (2 mg, 0.02 mmol, 10 mol %), and the solution was stirred at 25-70 °C until all the starting material has been consumed. The mixture was cooled to room temperature, diluted with ether, washed twice with brine, dried with Na_2SO_4, filtered, and concentrated under reduced pressure. The residue was purified by silica gel flash column chromatography.

For SN38-tBu, ^{1}H NMR (400 MHz, Chloroform-*d*) δ 7.75 (d, J = 10.2 Hz, 2H), 7.28 (s, 47H), 5.71

(d, *J* = 17.2 Hz, 3H), 5.38 (d, *J* = 17.2 Hz, 3H), 5.33 – 5.18 (m, 6H), 2.96 – 2.84 (m, 3H), 2.11 (dd, *J* = 14.3, 7.5 Hz, 4H), 1.49 – 1.32 (m, 19H), 1.00 (t, *J* = 7.5 Hz, 5H). HRMS: m/z=449.2078 (expected 449.1998 for $[M+H]^+$).

For SN38-Boc, ^1H NMR (400 MHz, Chloroform-*d*) δ 8.01 (s, 1H), 7.75 (d, *J* = 10.2 Hz, 2H), 7.54 (s, 1H), 6.58 (d, *J* = 10.2 Hz, 3H), 5.71 (d, *J* = 17.2 Hz, 3H), 5.38 (d, *J* = 17.2 Hz, 3H), 5.31 (s, 3H), 5.26 – 5.12 (m, 5H), 3.12 – 2.83 (m, 3H), 2.35 – 2.03 (m, 5H), 1.50 – 1.32 (m, 20H), 1.00 (t, *J* = 7.5 Hz, 6H). HRMS: m/z=493.1970 (expected 493.1897for $[M+H]^+$).

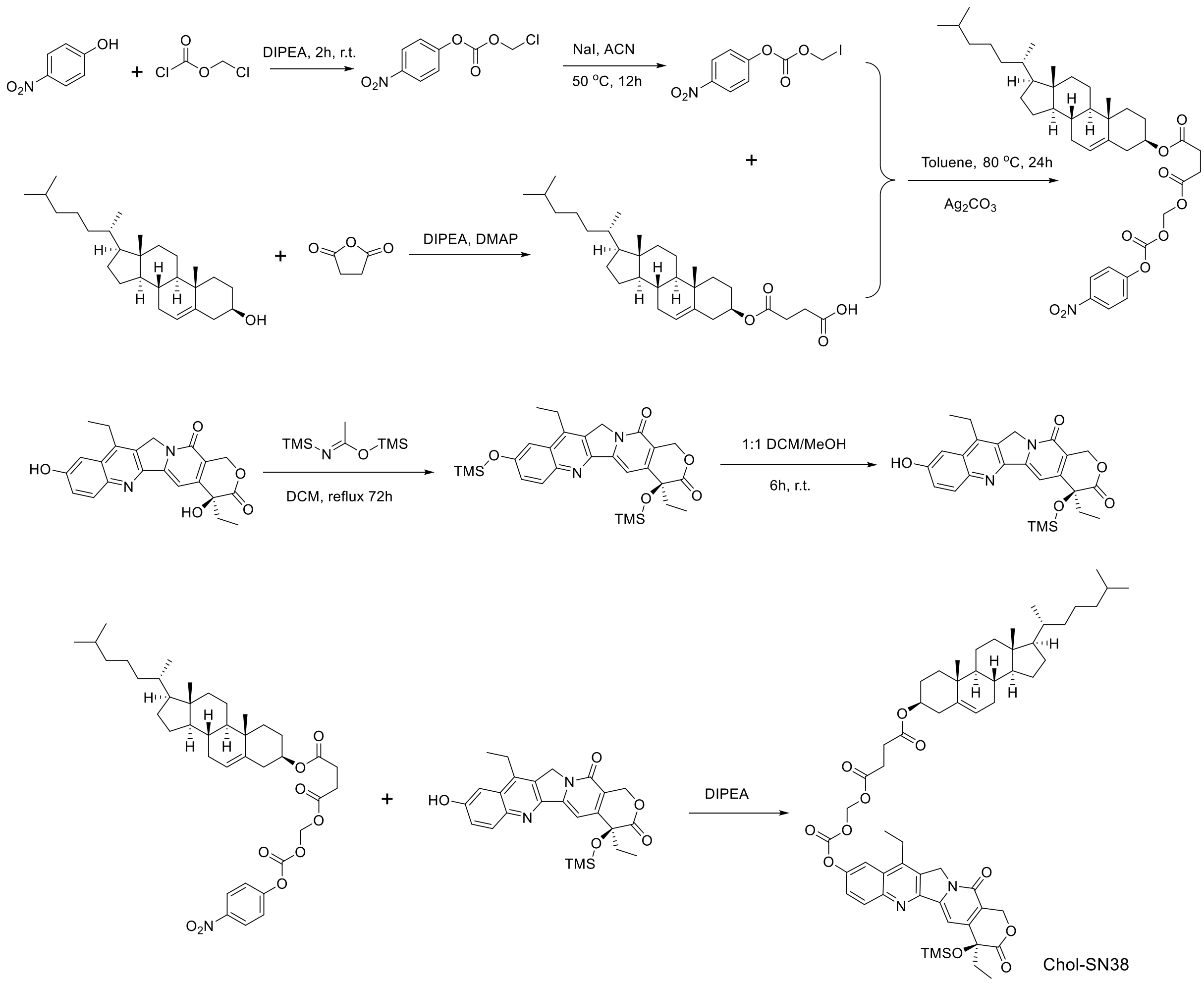

Figure S1 Synthesis of chol-SN38.

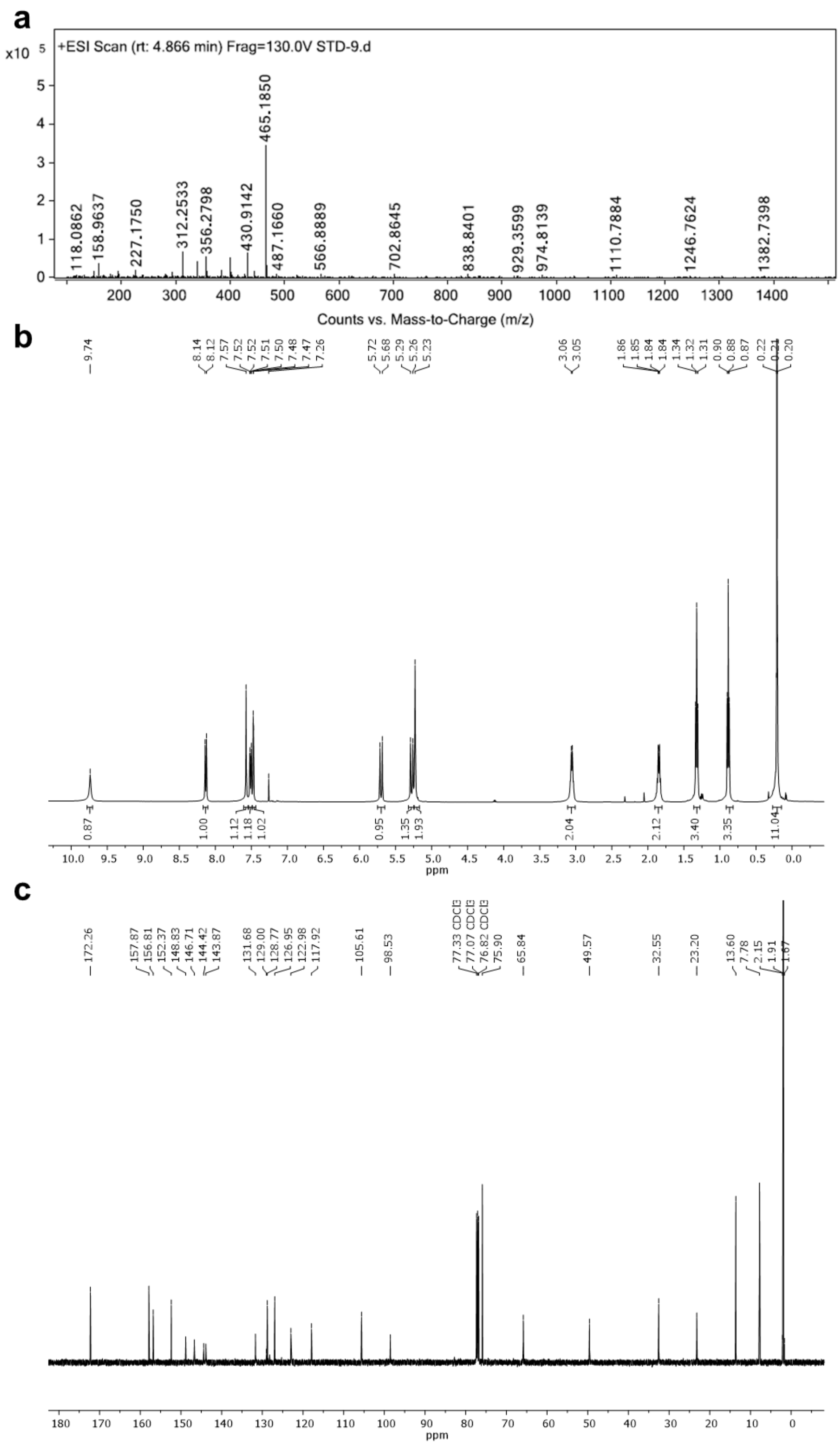

Figure S2 (a) HRMS of 20-O-trimethylsilyl-SN38 (SN38-TMS). (b) ^{1}H NMR spectrum of SN38-TMS in $CDCl_3$. (c) ^{13}C NMR spectrum of SN38-TMS in $CDCl_3$.

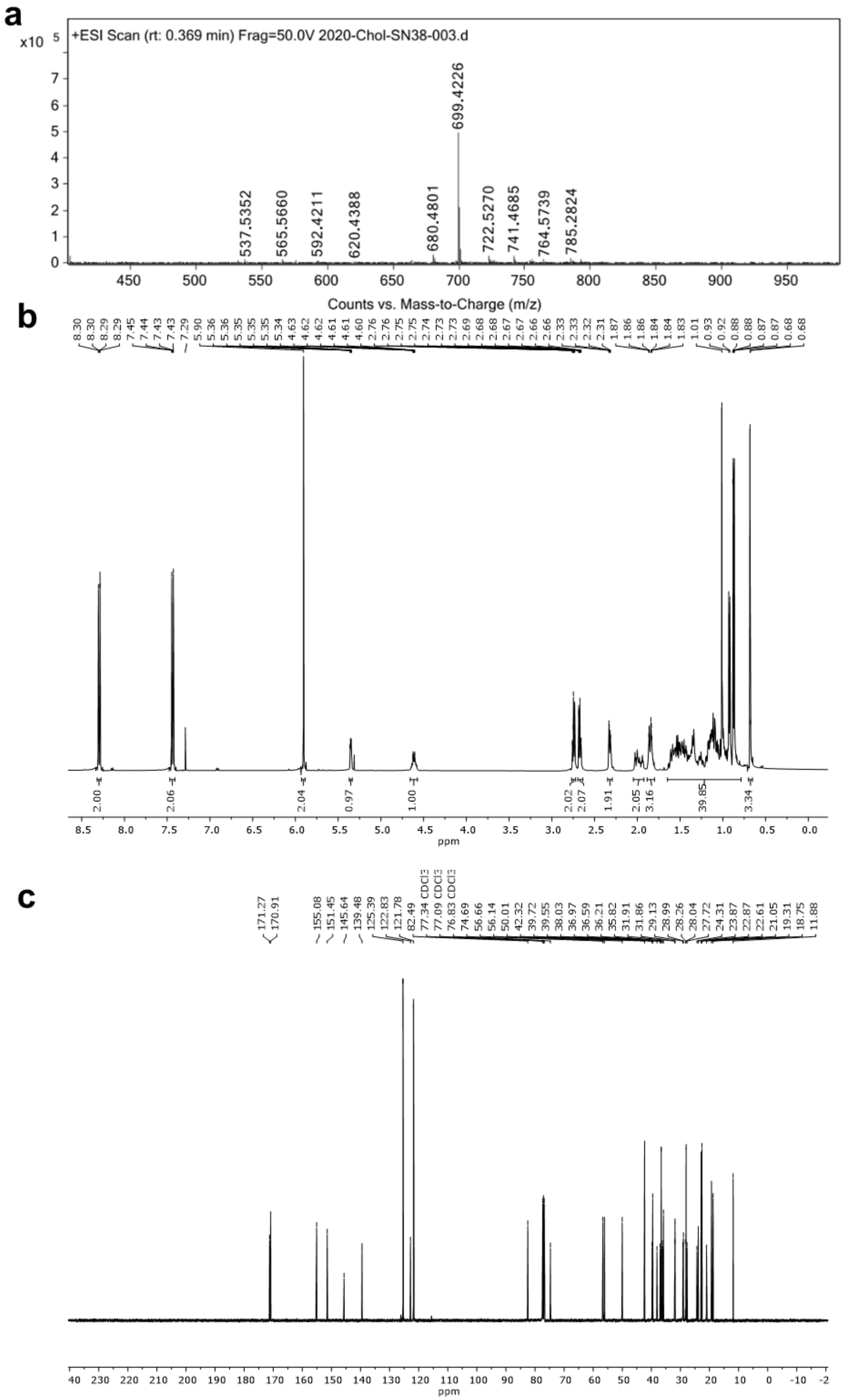

Figure S3 (a) HRMS of cholest-5-en-3-ol ((((4-nitrophenoxy)carbonyl)oxy) methyl) succinate (Chol-linker). (b) ^{1}H NMR spectrum of Chol-linker in $CDCl_3$. (c) ^{13}C NMR spectrum of Chol-linker in $CDCl_3$.

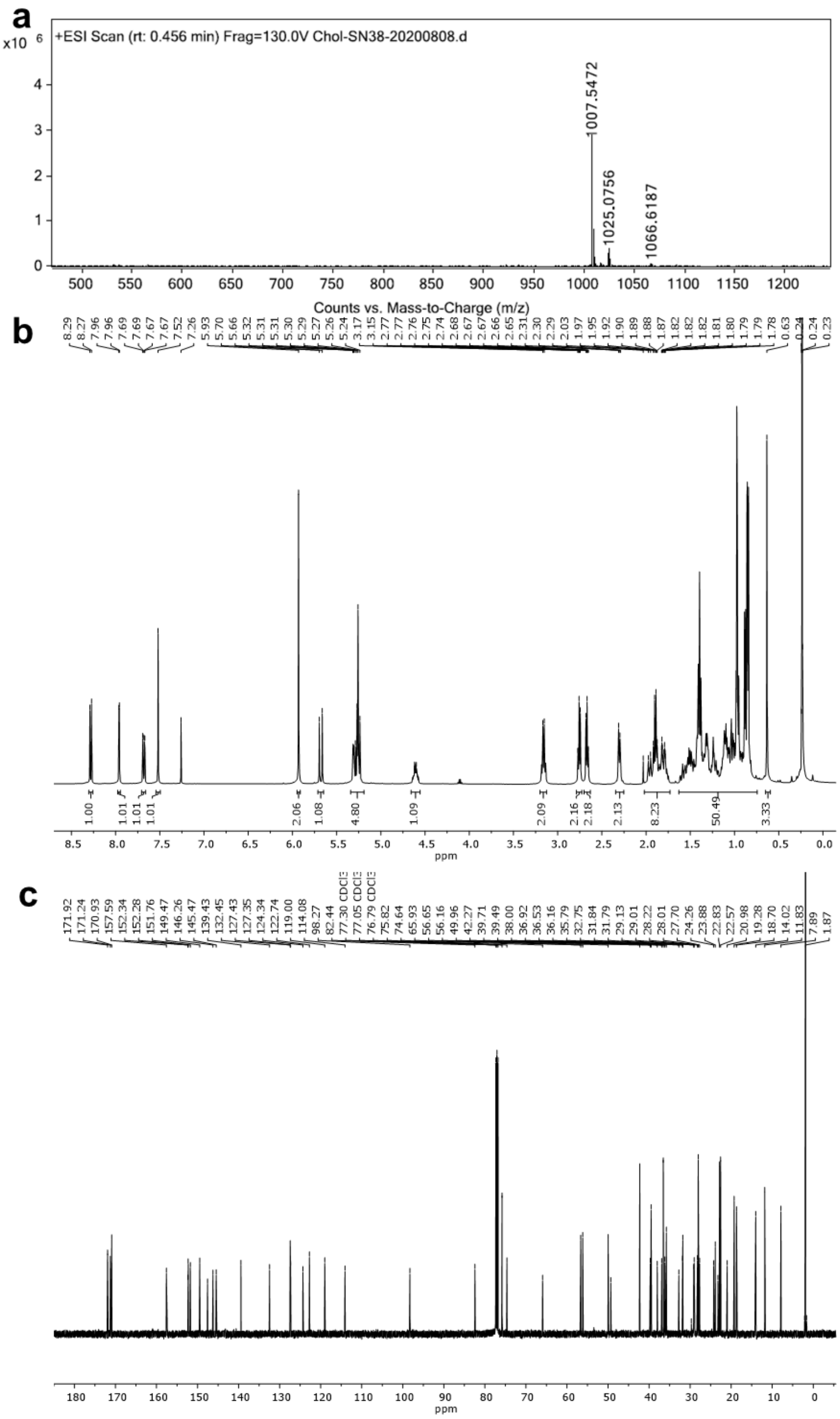

Figure S4 (a) HRMS of Chol-SN38. (b) ^{1}H NMR spectrum of Chol-SN38 in $CDCl_3$. (c) ^{13}C NMR spectrum of Chol-SN38 in $CDCl_3$.

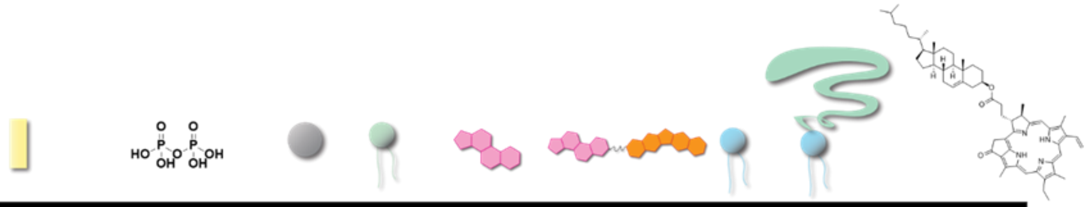

NCP	OxPt-bp	Pyrophosphate	Zn^{2+}	DOPA	Cholesterol	Chol-SN38	DOPC	DSPE-Peg	Chol-pyro
OxPt/SN38	√		√	√	√	√	√	√	
ZnP NCP		√	√	√	√		√	√	
ZnP SN38		√	√	√	√	√	√	√	
OxPt NCP	√		√	√	√		√	√	
OxPt NCP w/o chol	√		√	√			√	√	
ZnP NCP w/o chol		√	√	√			√	√	
Chol-pyro NCP		√	√	√	√		√	√	√
	Core				Shell				

Figure S5 Compositions of all the NCPs investigated in this work.

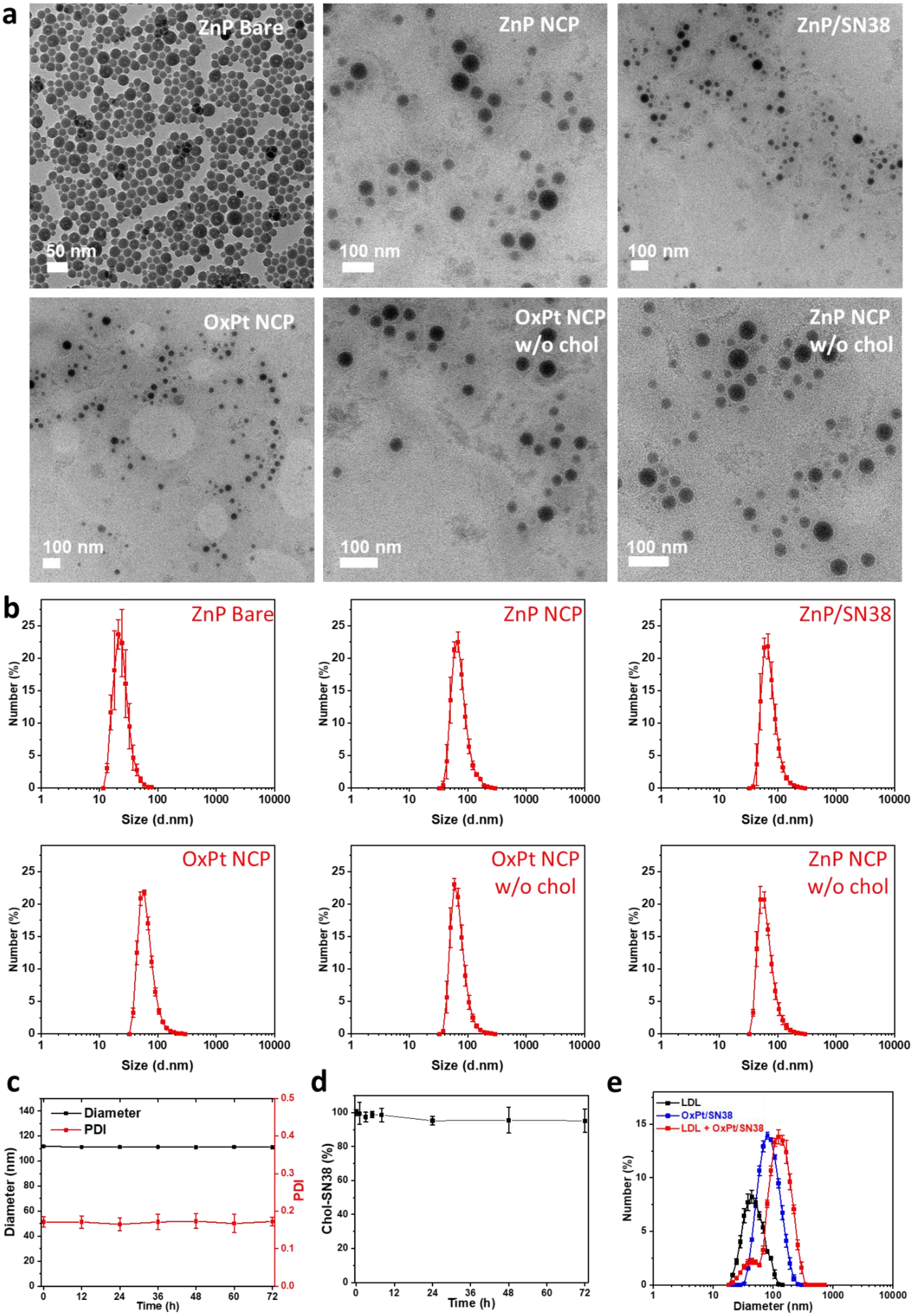
a
ZnP Bare
50 nm
ZnP NCP
100 nm
ZnP/SN38
100 nm
OxPt NCP
100 nm
OxPt NCP w/o chol
100 nm
ZnP NCP w/o chol
100 nm
b
ZnP Bare
ZnP NCP
ZnP/SN38
OxPt NCP
OxPt NCP w/o chol
ZnP NCP w/o chol
Number (%)
Size (d.nm)
c
Diameter
PDI
Diameter (nm)
PDI
Time (h)
d
Chol-SN38 (%)
Time (h)
e
LDL
OxPt/SN38
LDL + OxPt/SN38
Number (%)
Diameter (nm)

Figure S6 TEM images (a) DLS (b) of NCPs with various formulations. ZnP Bare: bare particles with zinc pyrophosphate core. ZnP NCP: NCP particles with zinc pyrophosphate core. ZnP/SN38: NCP particles with zinc pyrophosphate core and Chol-SN38 in the shell. OxPt NCP: NCP particles with OxPt core. OxPt NCP w/o chol: NCP particles with OxPt core without cholesterol in the shell. ZnP NCP w/o chol: NCP particles with zinc pyrophosphate core without cholesterol in the shell. (c) Stability of OxPt/SN38 in PBS with BSA (5 mg/mL) at 37 °C. (d) Stability of Chol-SN38 in OxPt/SN38 in PBS at 37 °C. (e)Number-average diameter of LDL at a concentration of 1 mg/mL (based on LDL cholesterol) or/and OxPt/SN38 at a concentration of 53 mg/mL (based on hol/SN38) in PBS.

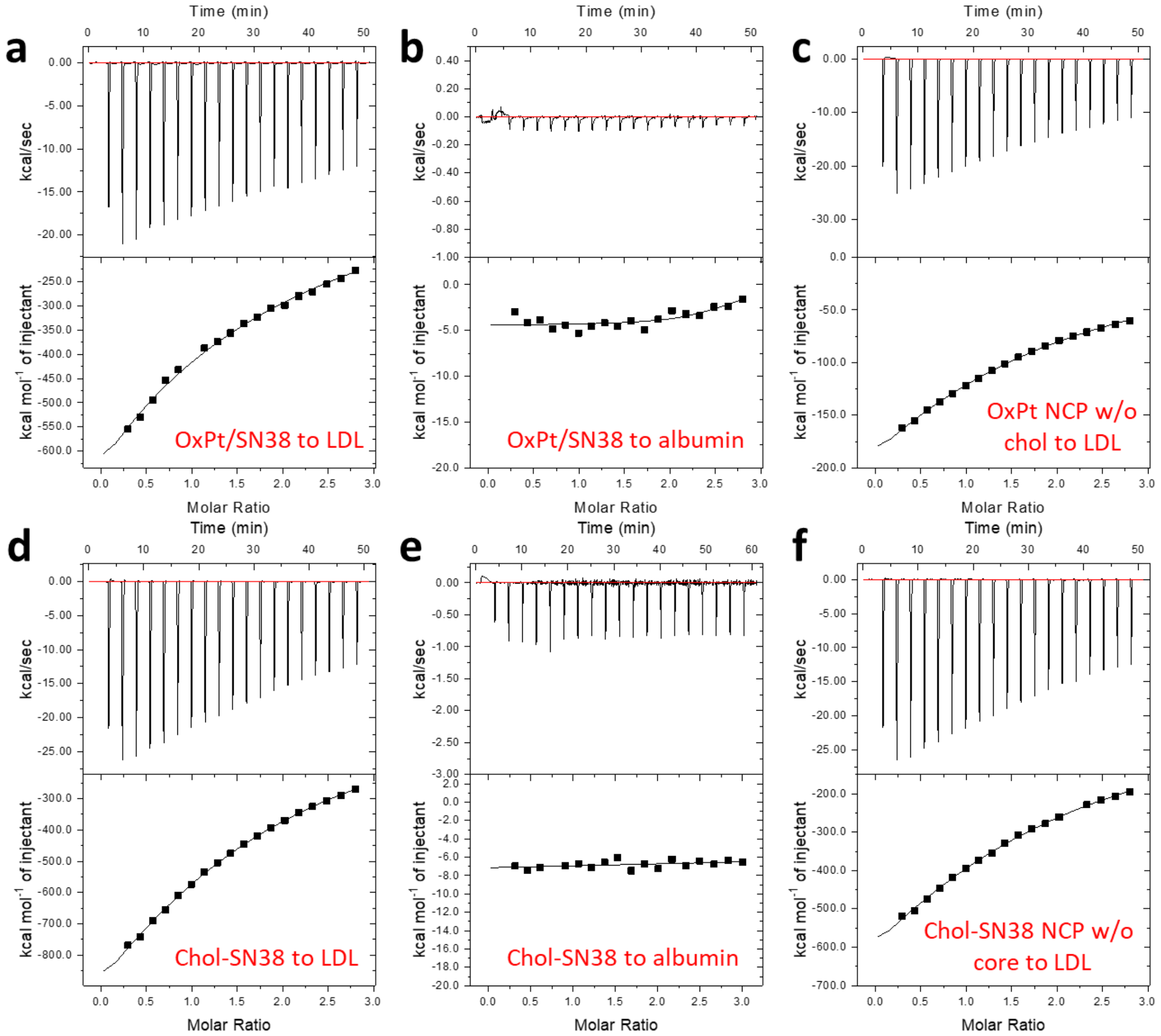

Figure S7 Isothermal titration calorimetry analysis. Isothermal titration calorimetry analysis of the interactions: between OxPt/SN38 and LDL (a) or albumin (b); between Chol-SN38 and LDL (d) or albumin (e); between OxPt NCP w/o chol (c) or Chol-SN38 particles w/o core (f) and LDL.

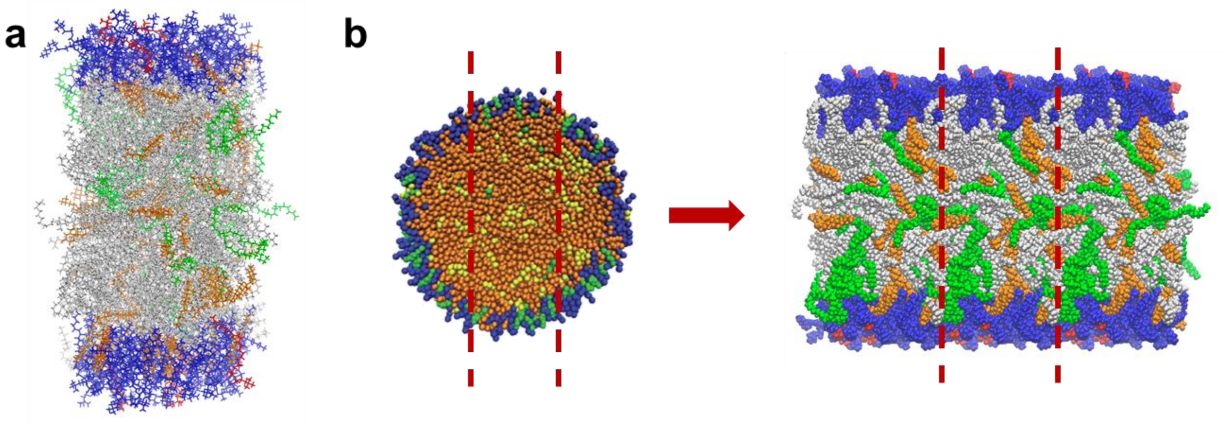

Figure S8 (a) Snapshot of equilibrated LDL slice in a single periodic cell. Glyceryl trioleate, cholesterol oleate, cholesterol, POPC, and lyso PC are shown in green, gray, orange, blue and red, respectively. Water molecules are not shown for clarity. (b) Scheme showing the simplified LDL model. (arrow left) Coarse-grained model of a whole spherical LDL[3]. (arrow right) The slice is simulated with periodic boundaries as in lipid bilayer MD simulations. Red dashed lines are boundaries of periodic cells.

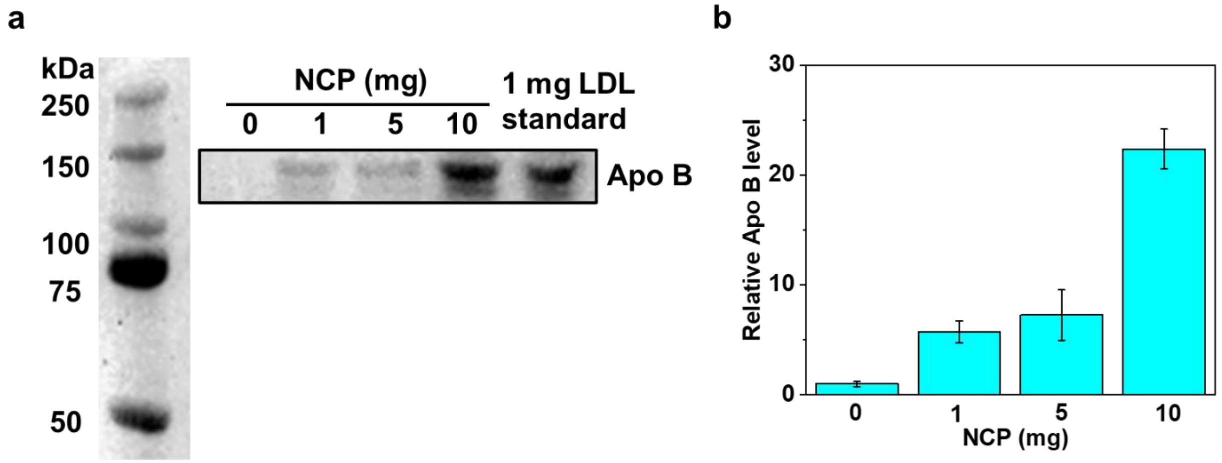

Figure S9 Western blot characterization (a) and relative levels (b) of NCP-bound Apo B proteins (in LDL) from mouse plasma.

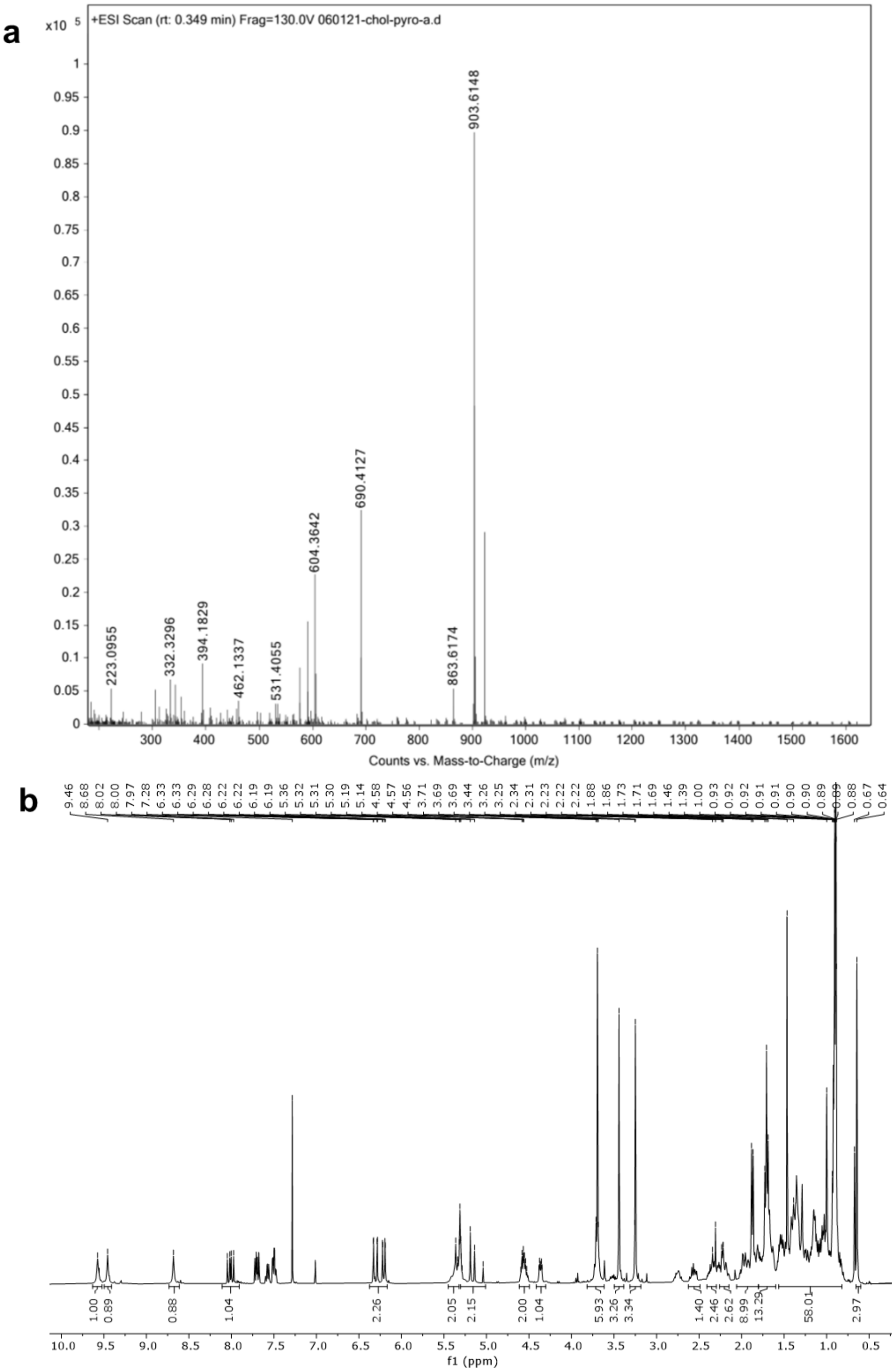

Figure S10 (a) HRMS of Chol-pyro. (b) ^{1}H NMR spectrum of Chol-pyro in $CDCl_3$.

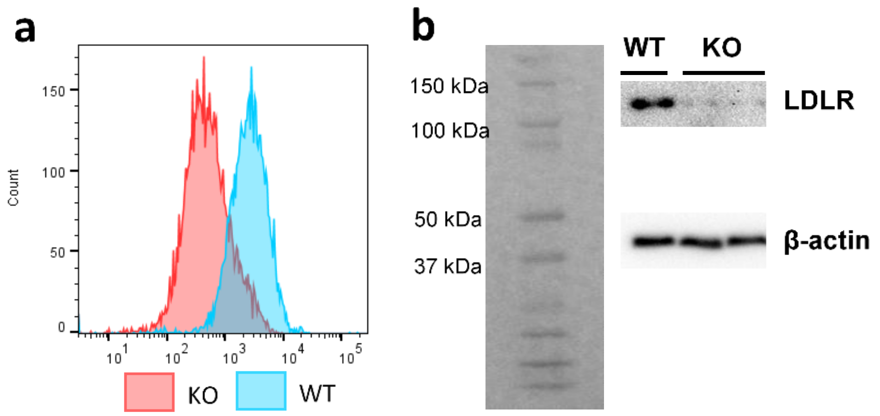

Figure S11 The relative LDLR expression by flow cytometry (a) and western blot (b) on WT and LDLR KO MC38 cell lines.

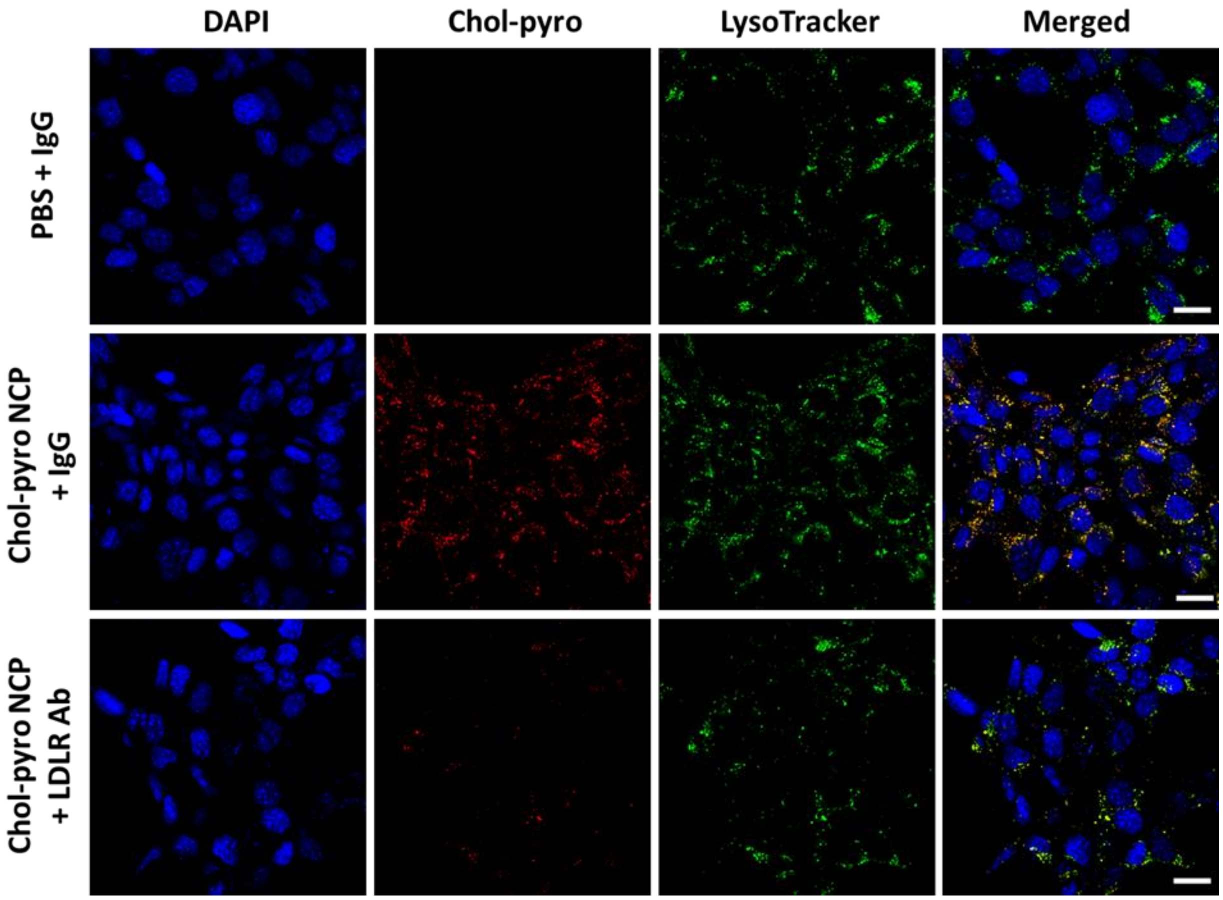

Figure S12 CLSM images of cellular uptake of Chol-pyro NCP with 10 µg/ml LDLR antibody blocking. LysoTracker was stained to visualize the localization of Chol-pyro in lysosomes. Scale bar, 20 µm.

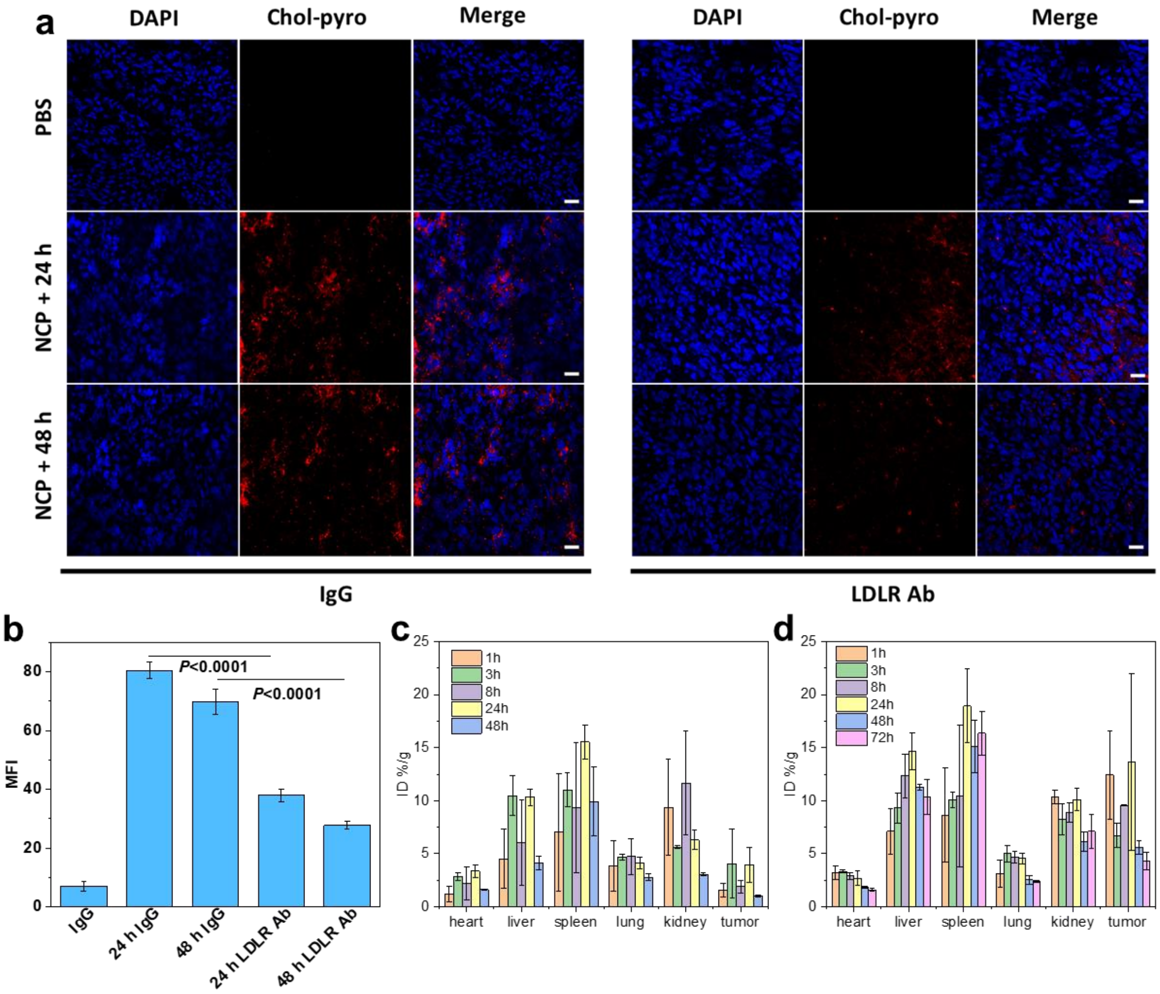

Figure S13 (a) Immunofluorescence analysis showing chol-pyro tumor uptake 24 h and 48 h post i.v. injection of Chol-pyro NCP. The mice were also intratumorally injected with 1 ug of IgG or LDLR Ab. Scale bar: 20 µm. (b) Mean fluorescent intensity analysis of Chol-pyro on CLSM images. (c,d) Time dependent Pt accumulation after i.v. injection of free OxPt (c) or OxPt/SN38 (d, 3.5 mg OxPt/kg equivalent, 6.2 mg SN38/kg equivalent) to MC38-bearing mice.

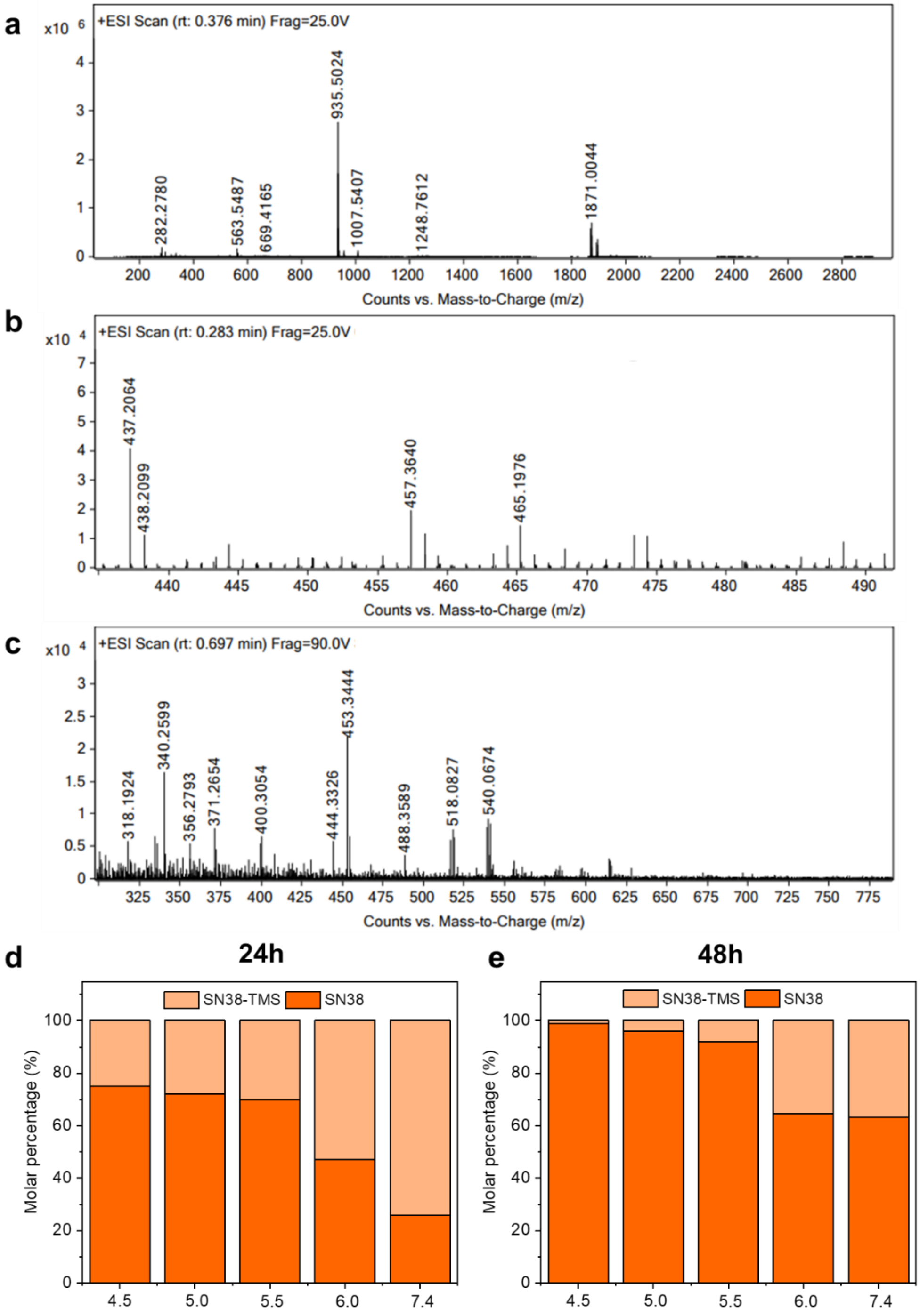

Figure S14 HR-MS showing the release mechanisms of drug release. (a) Chol-SN38 decomposition product in pH = 4.7 aqueous solution for 5 h. 1007.5407: Chol-SN38; 935.5024: Chol-SN38 (No TMS). (b) Chol-SN38 decomposition product in 10 unit/ml esterase aqueous solution for 5 h. 465.1976: SN38-TMS. (c) Hydrolysis product released from particle core pH = 4.7 aqueous solution in 1 h. 540.0674: OxPt-bc. Conversion of SN38-TMS to SN38 in different pH conditions. PBS solutions of 20 ppm SN38-TMS were prepared in different pH conditions (pH = 4.5, 5.0, 6.0, and 7.4) and the amounts of SN38-TMS and SN38 were measured by LC-MS after incubation for 24 h (d) and 48h (e).

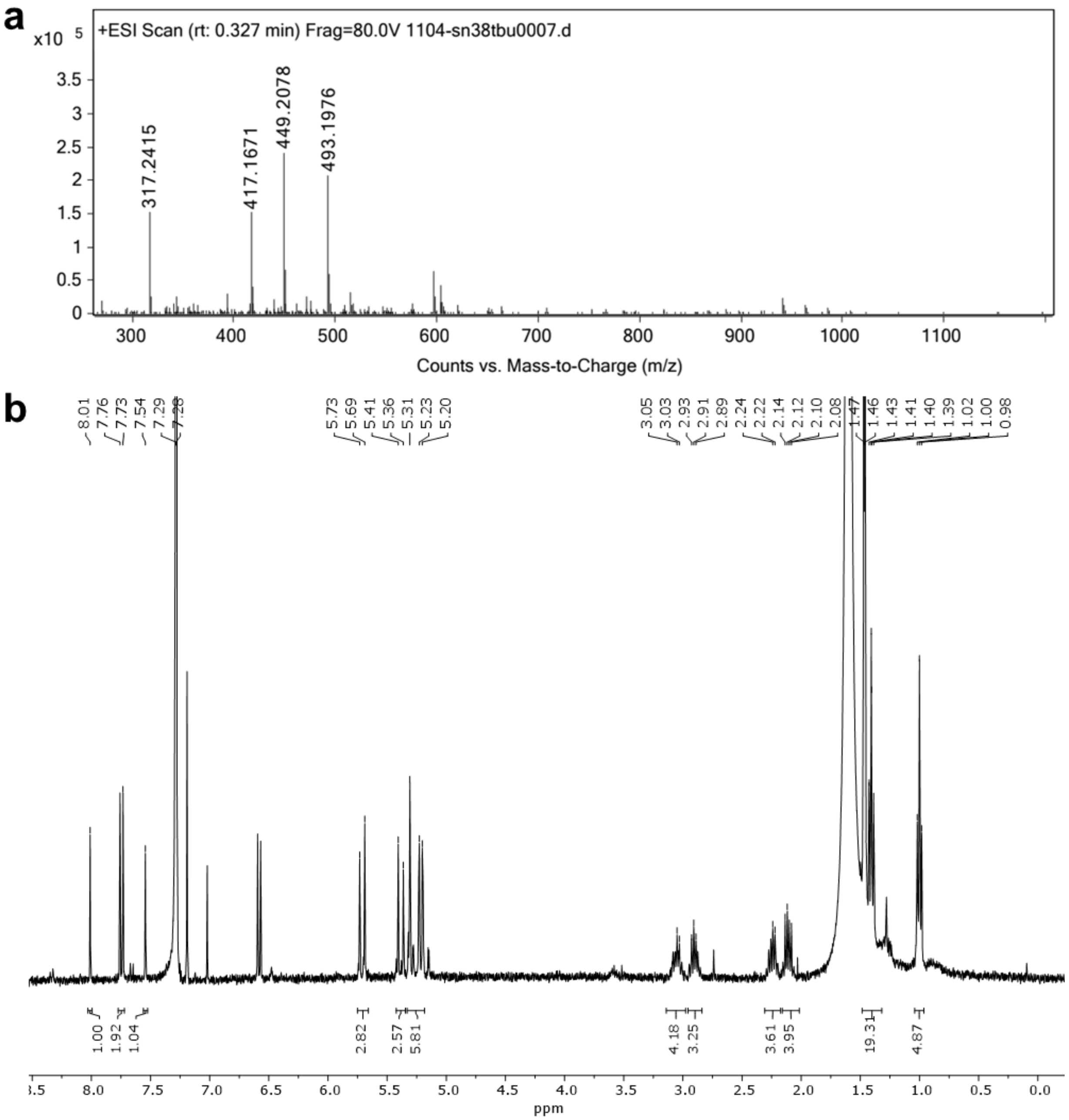

Figure S15 (a) HRMS of SN38-*t*Bu. (b) ^{1}H NMR spectrum of SN38-*t*Bu in $CDCl_3$.

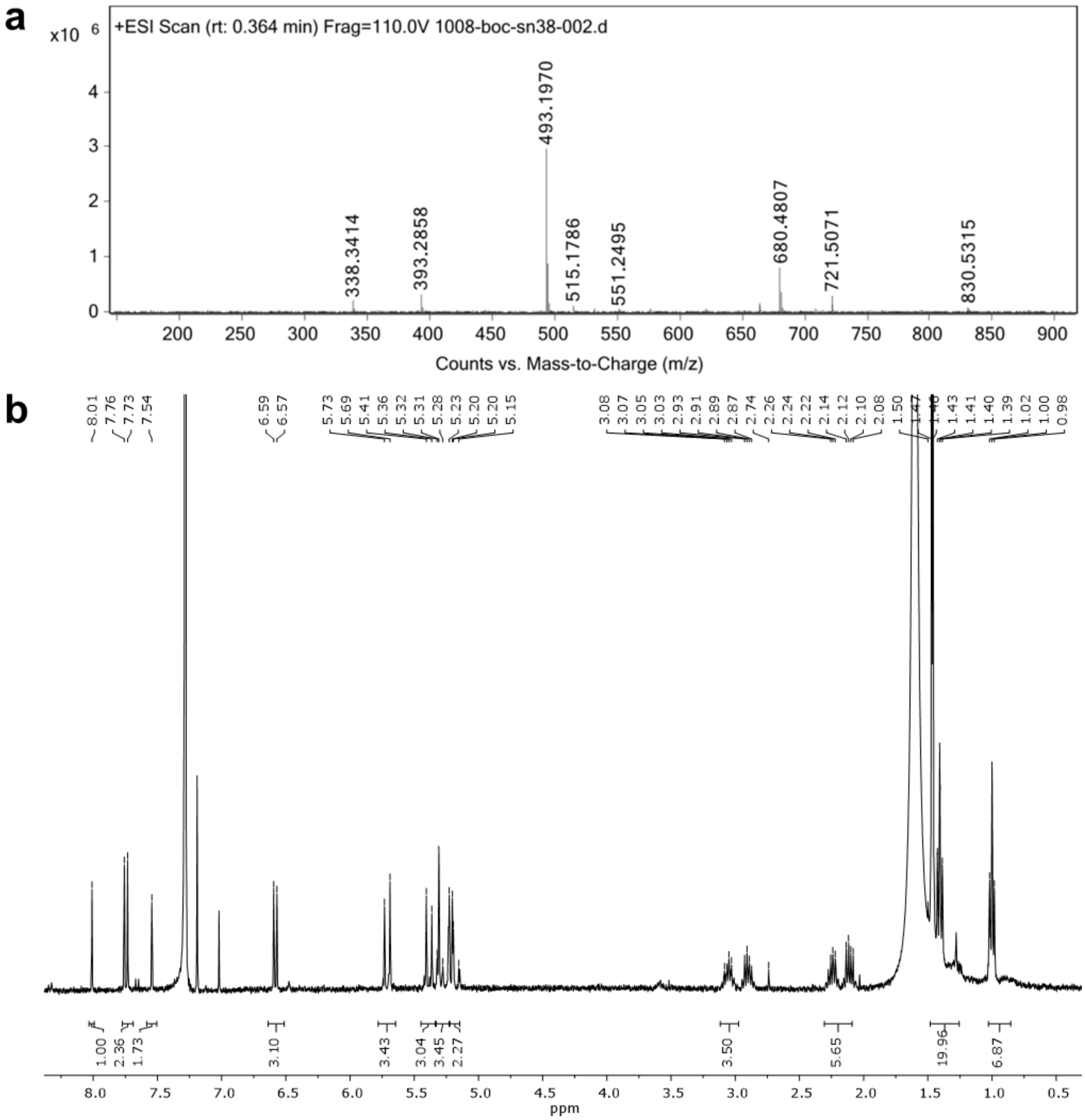

Figure S16 (a) HRMS of SN38-Boc. (b) ^{1}H NMR spectrum of SN38-Boc in $CDCl_3$.

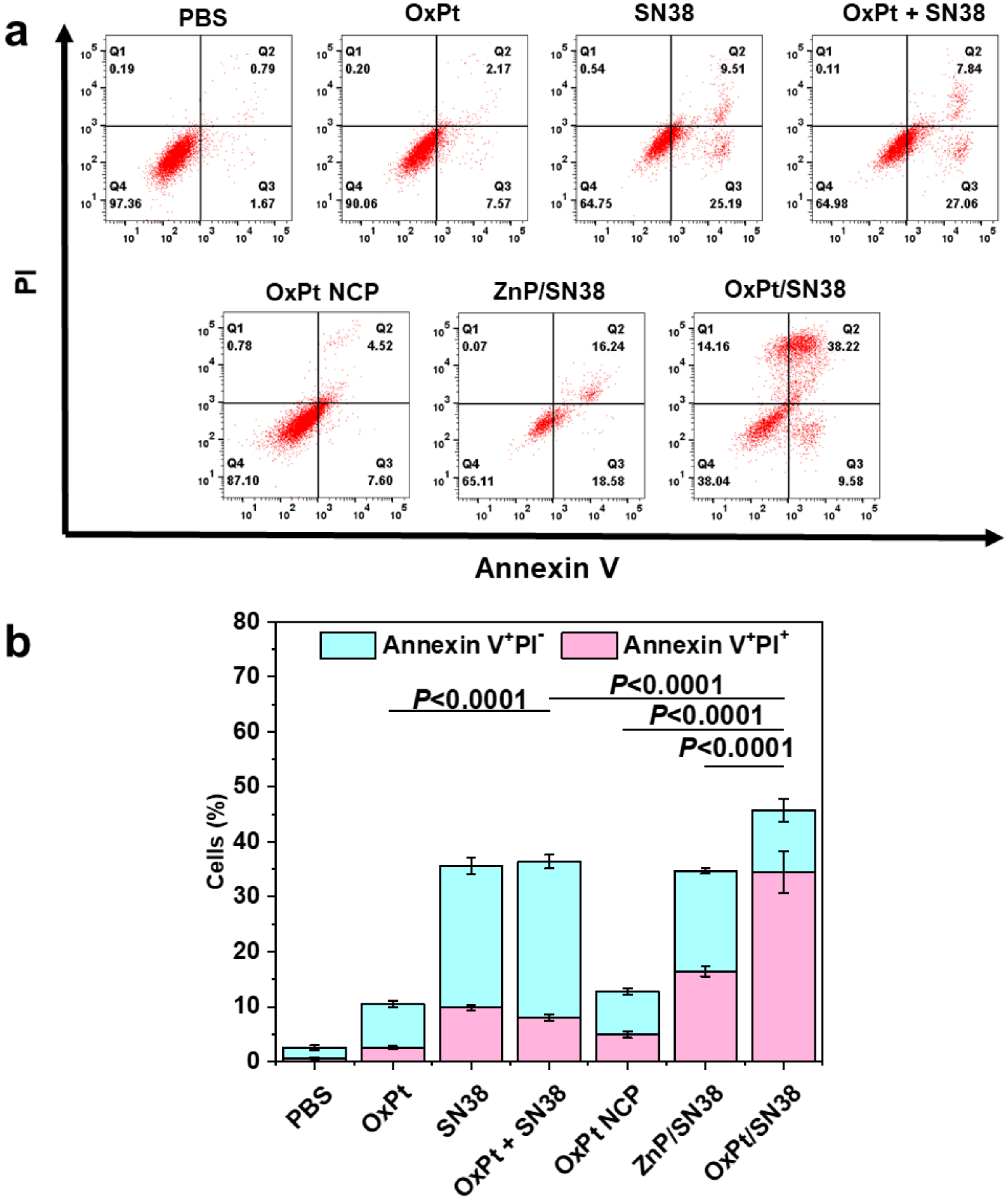

Figure S17 (a) Apoptosis induced by different treatments. 24 h after treatment, cells were stained by Alexa Fluor 488-labelled Annexin V and propidium iodide (PI) and analyzed by flow cytometry. (b) Statistical analysis results of apoptosis/necrosis as determined by flow cytometry. Data are expressed as means ± SD. The data were analyzed by one-way analysis of variance (ANOVA) with Tukey's multiple comparisons test.

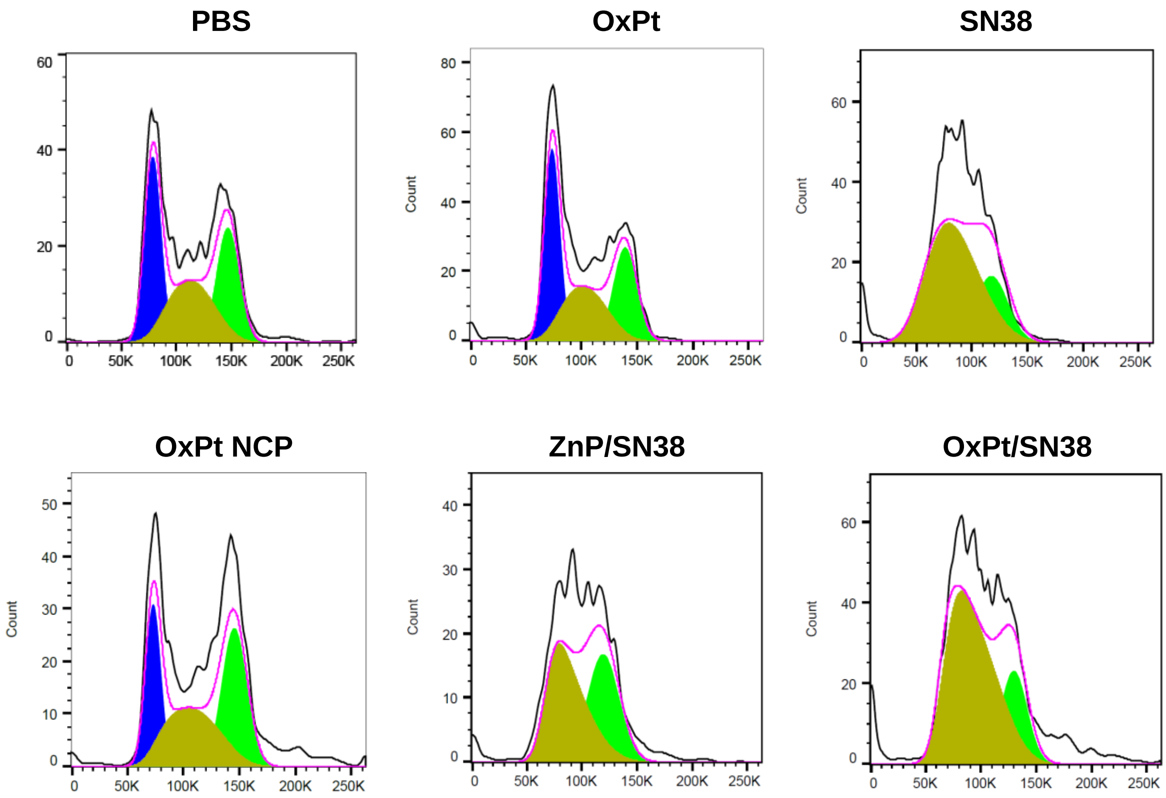

Figure S18 Cell cycle arrest caused by different treatments. Treated cells were fixed with 70% ethanol overnight, treated with RNase A, stained by PI, and analysed by flow cytometry. Blue: G1/G0 phase, yellow: S phase, green: G2/M phase.

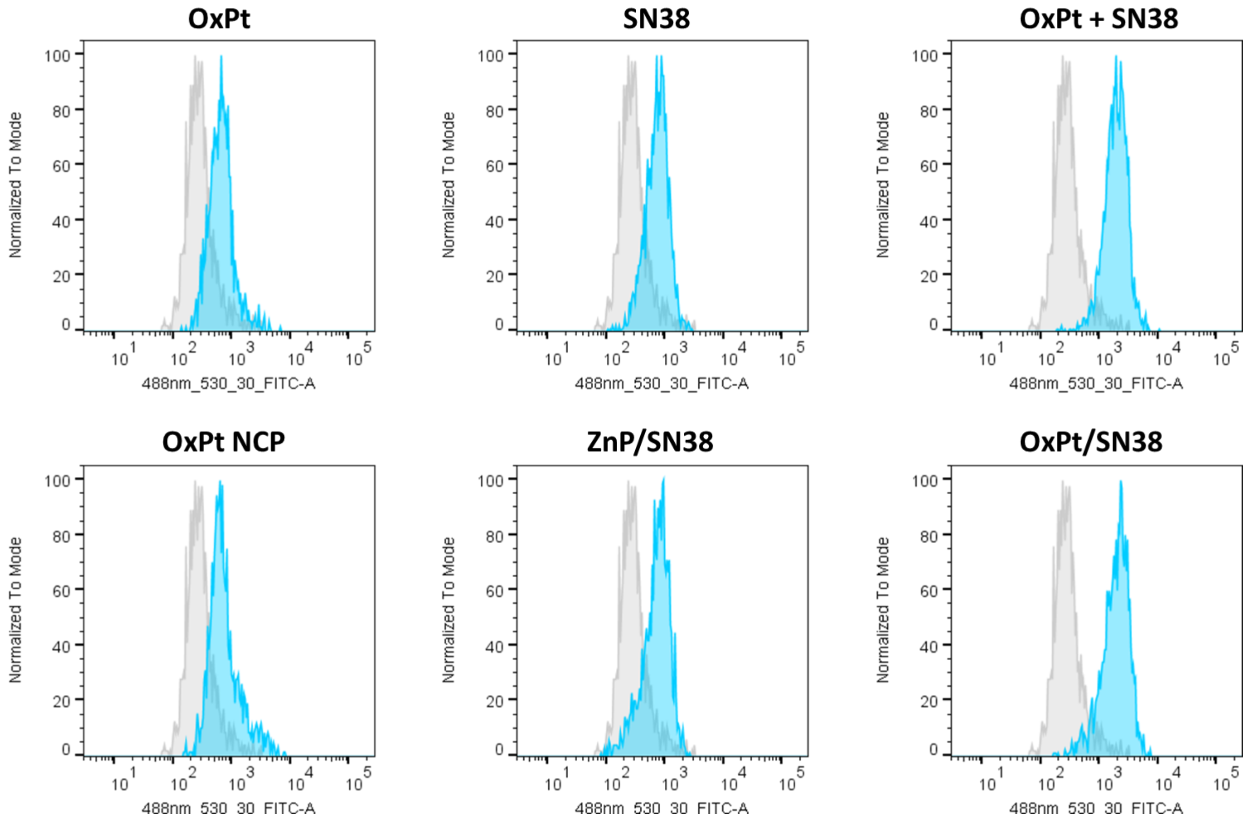

Figure S19 JC-1 analysis with flow cytometry for mitochondrial membrane potential of MC38 cells exposed to different treatments. Grey histogram: signals from PBS group.

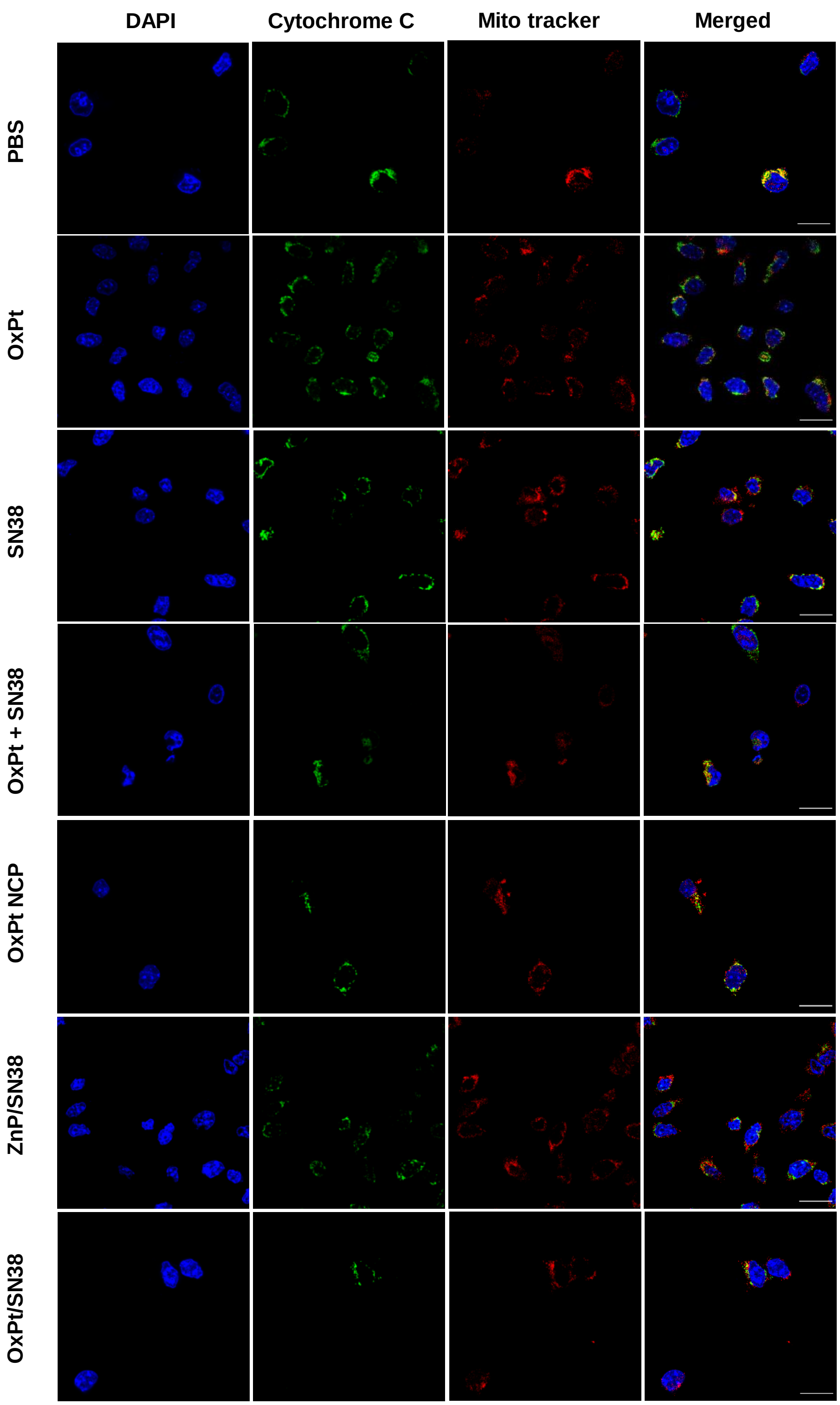

Figure S20 Release of cytochrome c from mitochondria in cells incubated with different treatments. Mitochondria (red fluorescence) and cytochrome c (green fluorescence) were stained by MitoTracker Red CMXRos and anti-cytochrome c antibody, respectively. Scale bar: 20 µm.

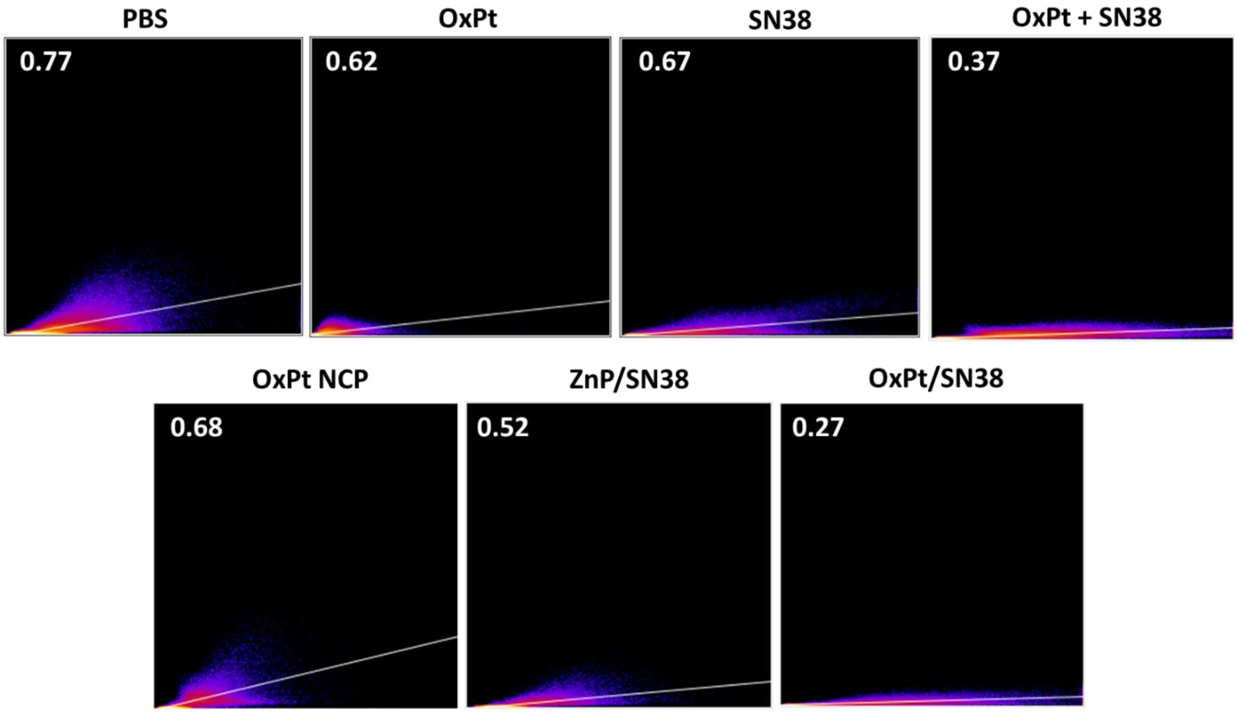

Figure S21 Pearson's R values of cytochrome c and mitochondria in cells incubated with different treatments.

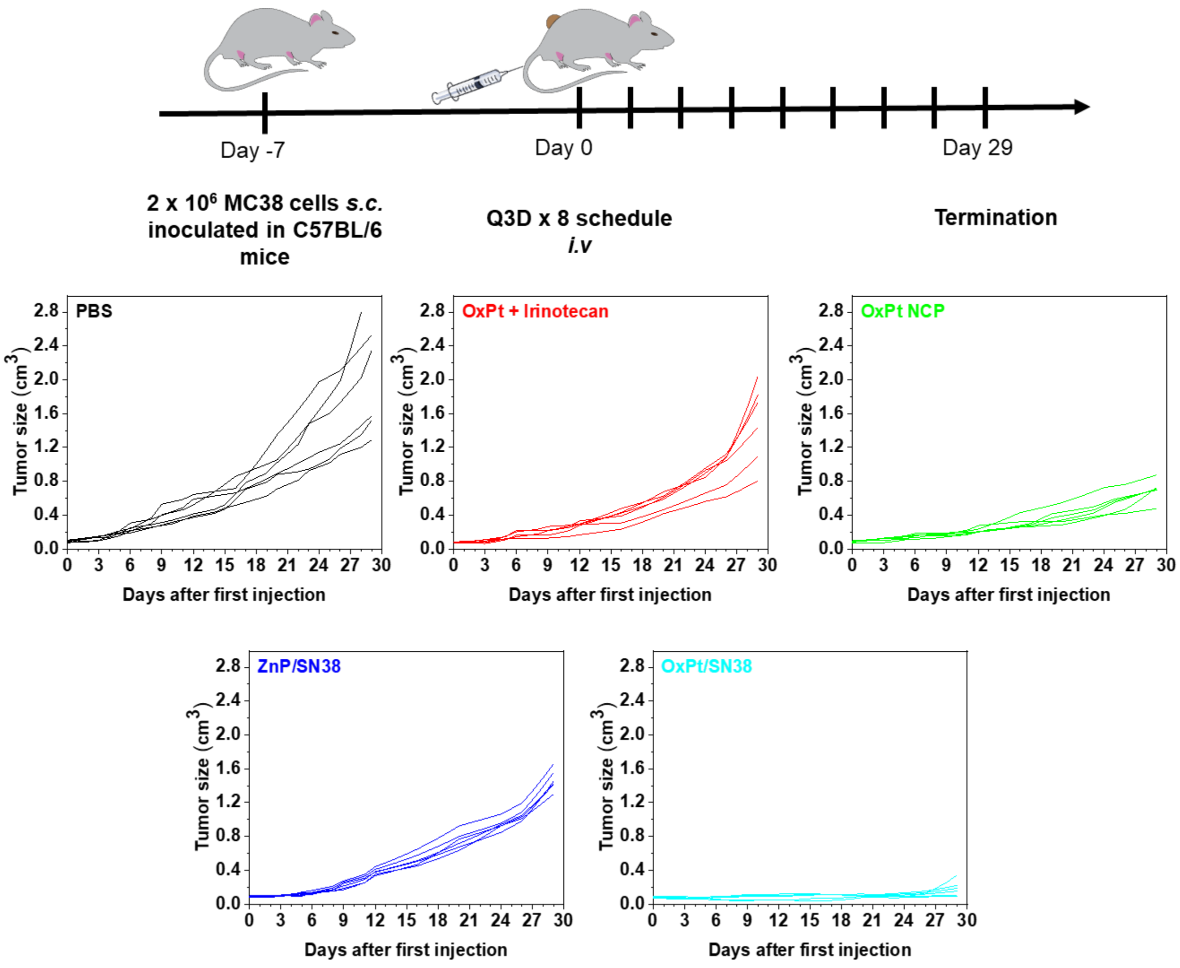

Figure S22 Treatment schema and individual tumor growth curves of MC38 tumors in C57BL/6 mice. PBS, OxPt plus irinotecan, OxPt NCP, ZnP/SN38, or OxPt/SN38 was i.v. injected once every 3 days (Q3D) at doses of 3.5 mg/kg OxPt or equivalent, 20.2 mg/kg irinotecan (11.7 mg/kg SN38 equivalent), and/or 15.9 mg Chol-SN38/kg (6.2 mg/kg SN38 equivalent) to MC38 tumor-bearing mice for up to 8 doses, n = 6.

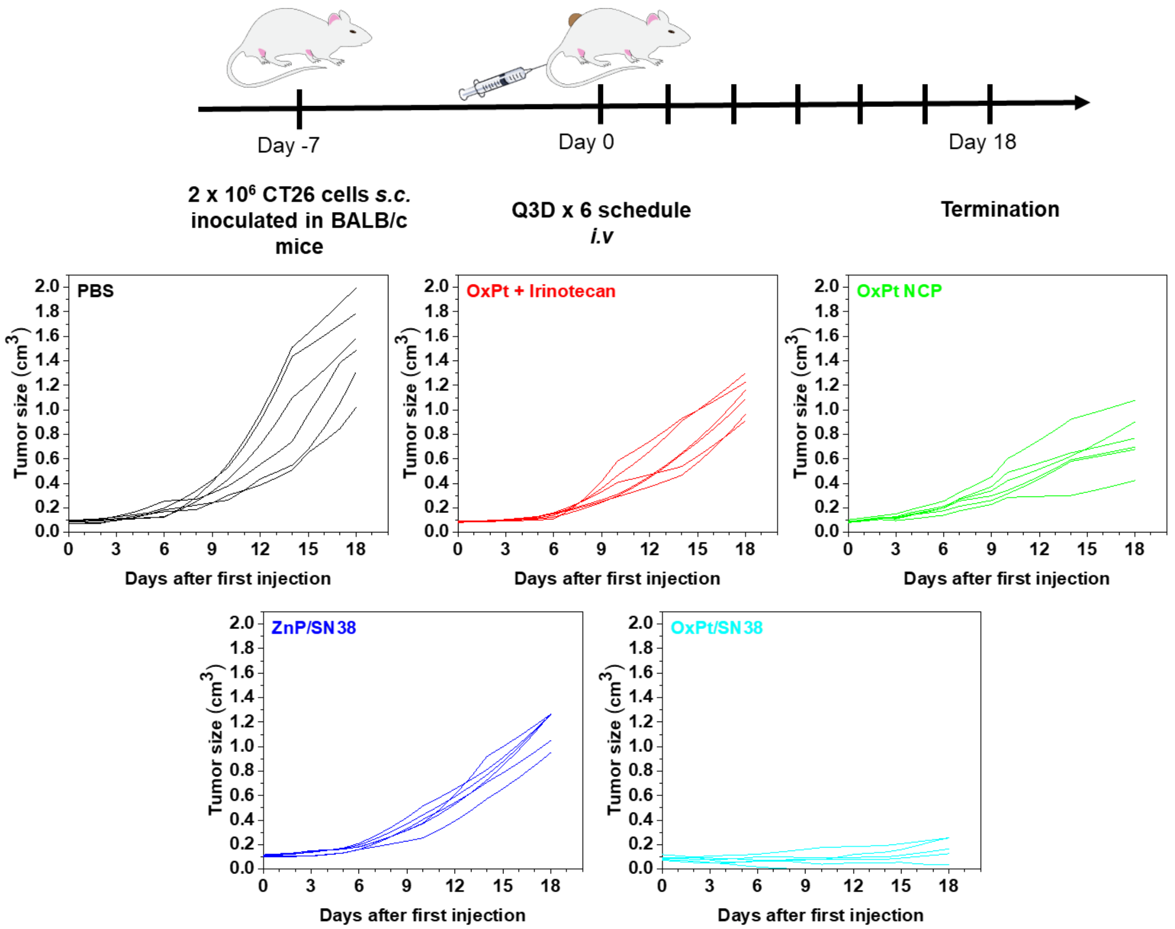

Figure S23 Treatment schema and individual tumor growth curves of CT26 tumors in BALB/c mice. PBS, OxPt plus irinotecan, OxPt NCP, ZnP/SN38, or OxPt/SN38 was i.v. injected once every 3 days (Q3D) at doses of 3.5 mg/kg OxPt or equivalent, 20.2 mg/kg irinotecan (11.7 mg/kg SN38 equivalent), and/or 15.9 mg Chol-SN38/kg (6.2 mg/kg SN38 equivalent) to CT26 tumor-bearing mice for up to 6 doses, n = 6.

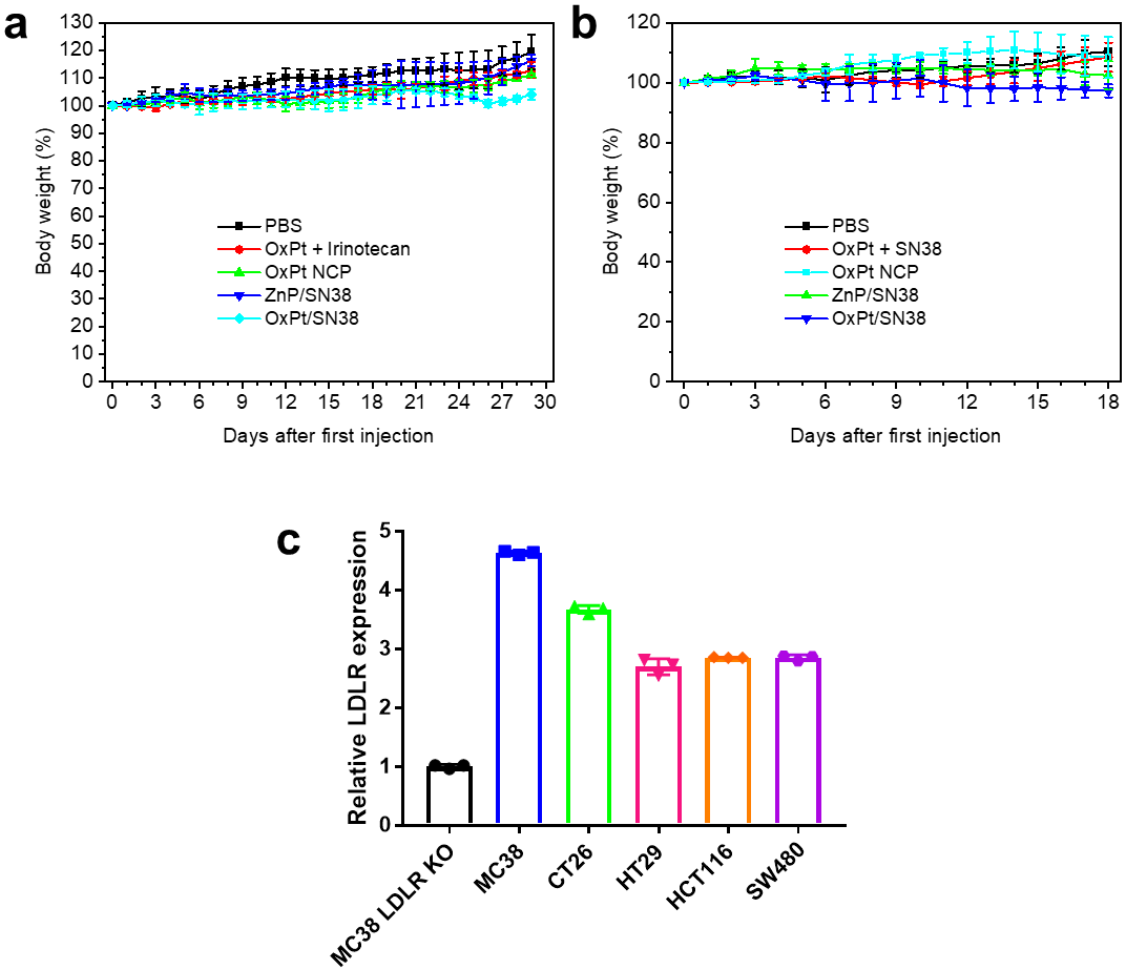

Figure S24 (a) Body weights of MC38 tumor-bearing C57BL/6 mice after treatment with PBS, OxPt + irinotecan, OxPt NCP, ZnP/SN38, or OxPt/SN38 at an equivalent dose of 3.5 mg/kg OxPt and/or 6.2 mg/kg SN38 (OxPt:SN38 = 1: 1.8) once every 3 days for up to 8 doses, n = 6. (b) Body weights of CT26 tumor-bearing BALB/c mice after treatment with PBS, OxPt + irinotecan, OxPt NCP, ZnP/SN38, or OxPt/SN38 at an equivalent dose of 3.5 mg/kg OxPt and/or 6.2 mg/kg SN38 (OxPt:SN38 = 1: 1.8) once every 3 days for up to 6 doses, n = 6. (c) Flow cytometry analysis of LDLR expression levels on various CRC cells. The signals were normalized to the MFI of MC38 LDLR KO cell line.

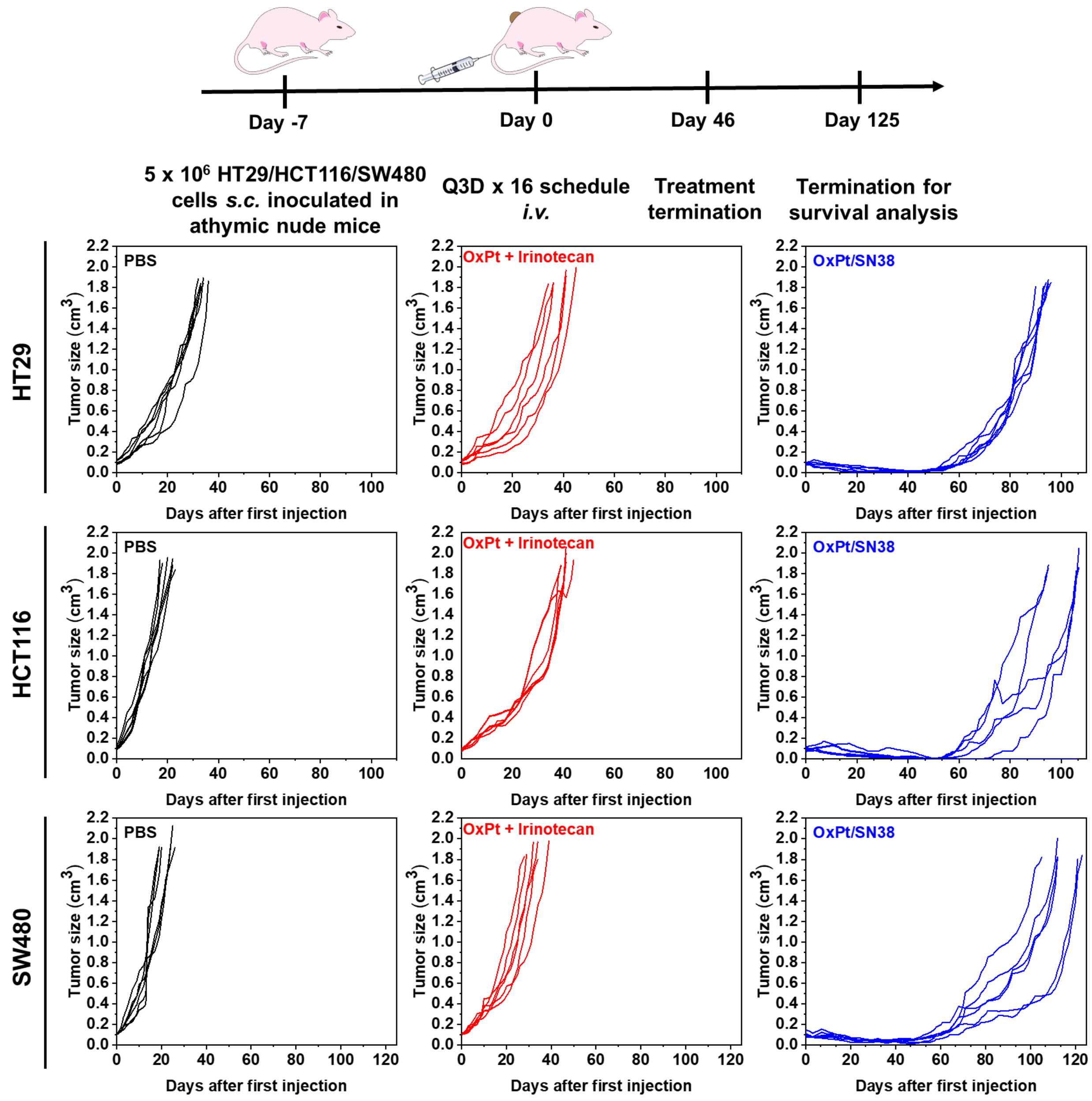

Figure S25 Treatment schema and individual tumor growth curves of HT29, HCT116 and SW480 models in nude mice after Q3D treatment with PBS, OxPt plus irinotecan, or OxPt/SN38 for up to 16 doses. PBS, OxPt plus irinotecan, or OxPt/SN38 was i.v. injected once every 3 days (Q3D) at doses of 3.5 mg/kg OxPt or equivalent, 20.2 mg/kg irinotecan (11.7 mg/kg SN38 equivalent), and/or 15.9 mg Chol-SN38/kg (6.2 mg/kg SN38 equivalent) to each mouse model for up to 16 doses, n = 6. The treatment of OxPt + irinotecan was terminated when the tumors reached the endpoint.

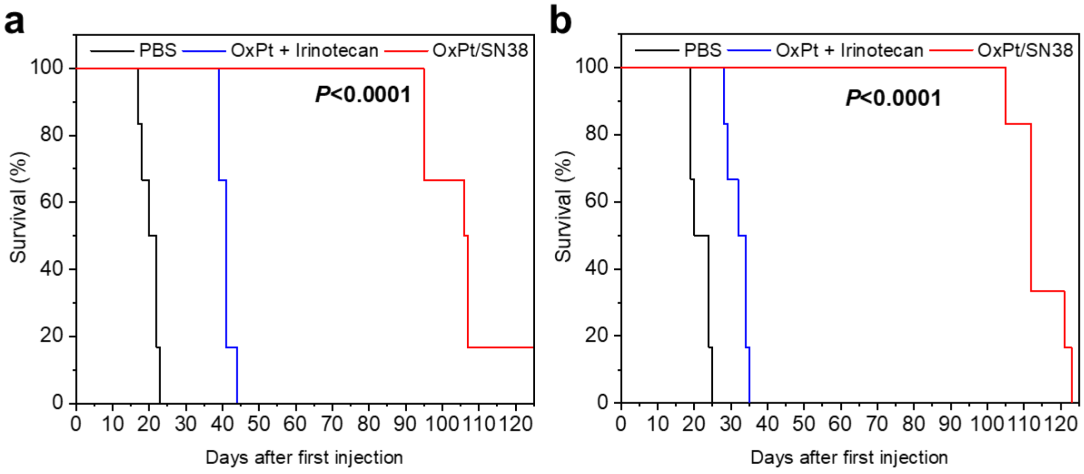

Figure S26 Survival curves of HCT116 and SW480 models in nude mice after Q3D treatment with PBS, OxPt plus irinotecan, or OxPt/SN38 for up to 16 doses. PBS, OxPt plus irinotecan, or OxPt NCP was i.v. injected once every 3 days (Q3D) at doses of 3.5 mg/kg OxPt or equivalent, 20.2 mg/kg irinotecan (11.7 mg/kg SN38 equivalent), and 15.9 mg Chol-SN38/kg (6.2 mg/kg SN38 equivalent) to CT26 tumor bearing mice for up to 16 doses, n = 6. The treatment of OxPt + irinotecan was terminated when the tumor reached the endpoint.

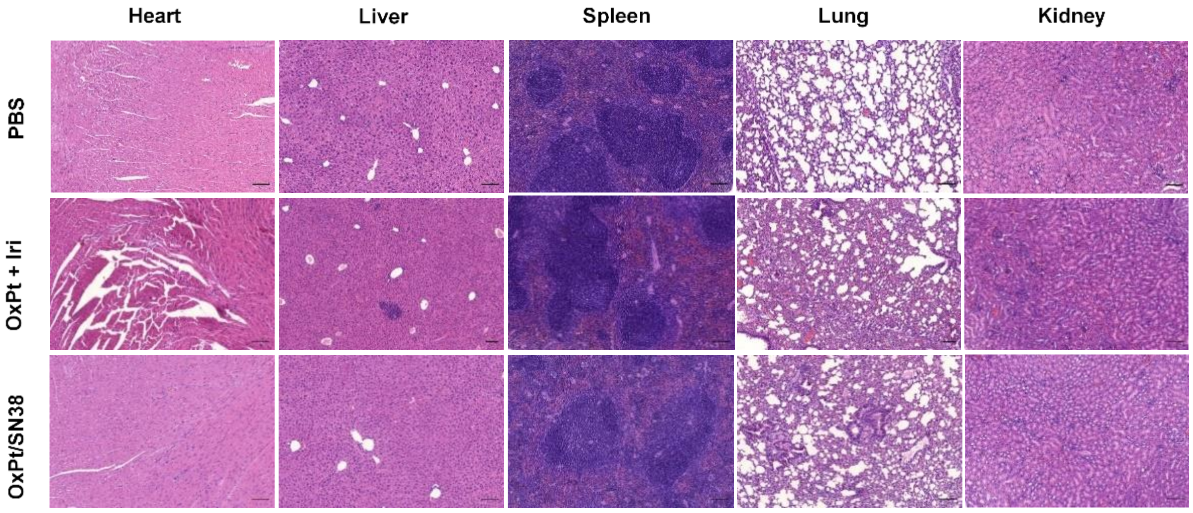

Figure S27 The histologies of major organs of MC38 bearing C57BL/6 mice by hematoxylin and eosin (H&E) staining. Scale bar = 100 µm.

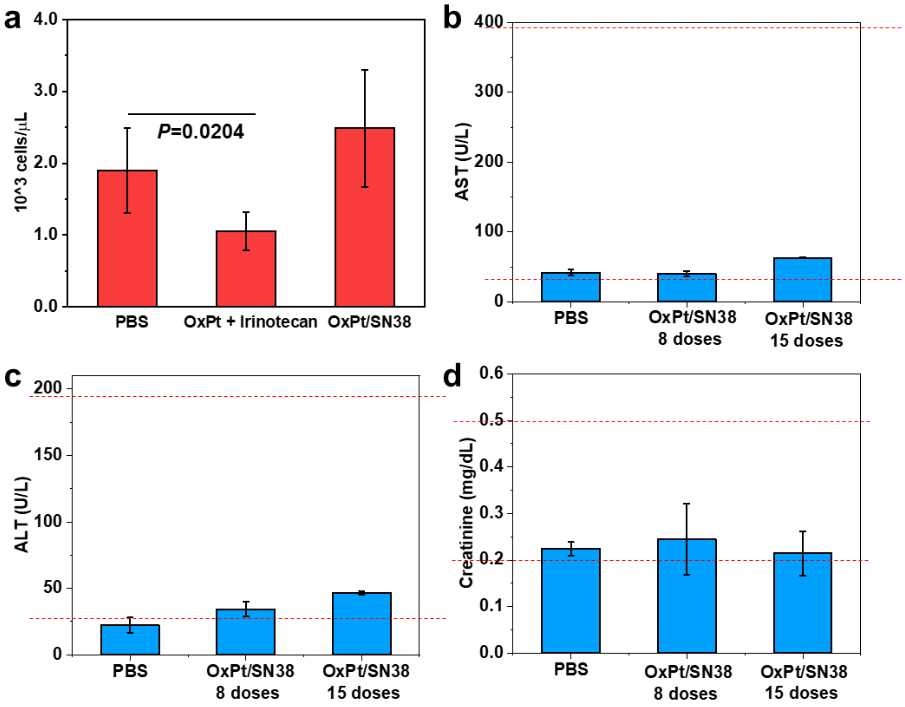

Figure S28 (a) Absolute neutrophil counts for MC38 tumor-bearing C57BL/6 mice after three Q3D doses of OxPt (3.5 mg/kg) plus irinotecan (11.7 mg/kg SN38 equivalent) or eight Q3D doses of OxPt/SN38 (3.5 mg/kg OxPt equivalent and 6.2 mg/kg SN38 equivalent). (b) AST, (c) ALT, and (d) serum creatinine levels for MC38 tumor-bearing C57BL/6 mice after 8 and 15 Q3D doses of OxPt/SN38 (3.5 mg/kg OxPt equivalent and 6.2 mg/kg SN38 equivalent). The dash lines show the normal ranges for C57BL/6 mice: 43-397 U/L for AST, 27-195 U/L for ALT, and 0.2-0.5 mg/dL for serum creatinine.

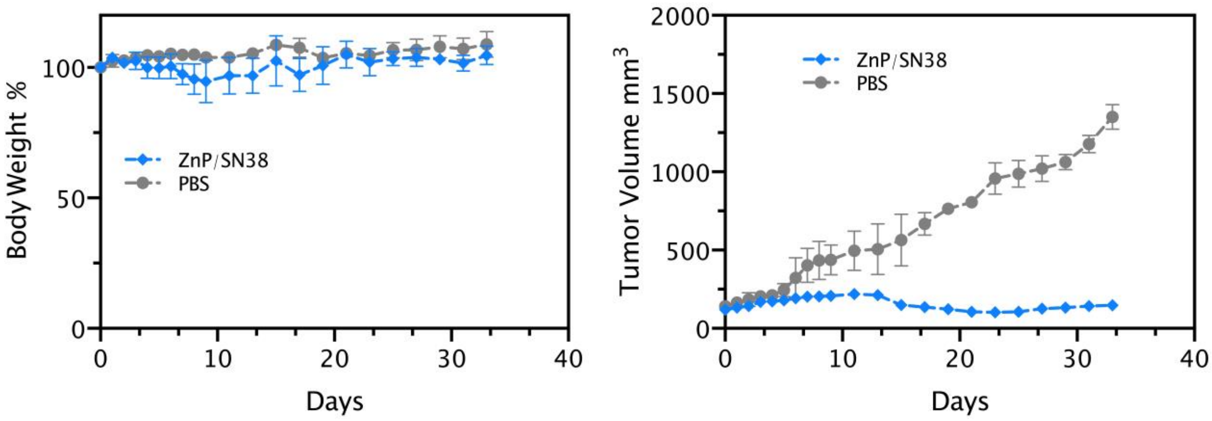

Figure S29 Body weight and tumor growth curves of HT29 tumor on nude mice after treatment with ZnP/SN38. Mice were i.v. injected with ZnP/SN38 at a dose of 34 mg Chol-SN38/kg once every 3 days for 12 doses, n = 3.

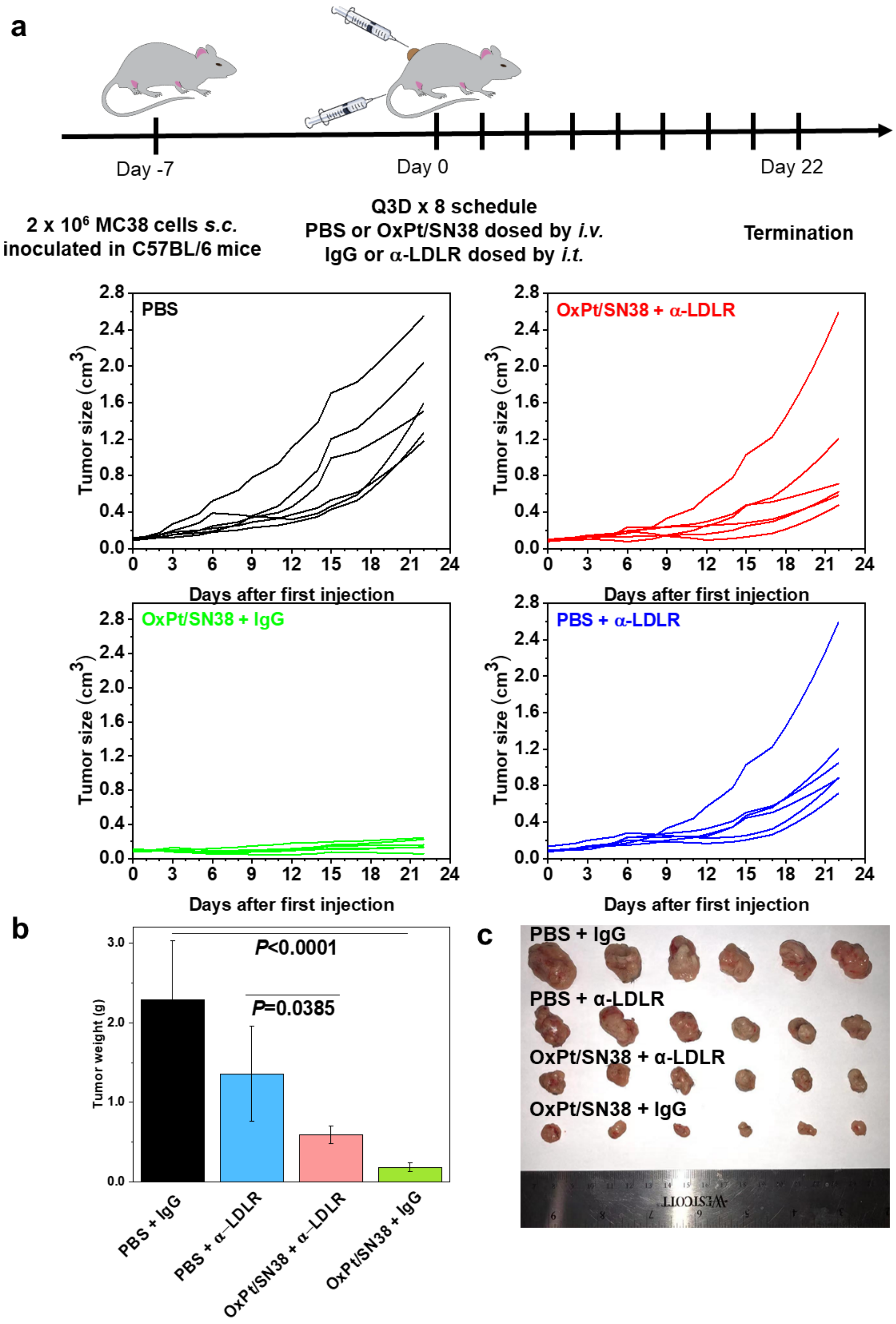
a
Day -7
Day 0
Day 22
2 x 10⁶ MC38 cells s.c. inoculated in C57BL/6 mice
Q3D x 8 schedule
PBS or OxPt/SN38 dosed by i.v.
IgG or α-LDLR dosed by i.t.
Termination
PBS
OxPt/SN38 + α-LDLR
OxPt/SN38 + IgG
PBS + α-LDLR
Tumor size (cm3)
Days after first injection
b
Tumor weight (g)
P<0.0001
P=0.0385
PBS + IgG
PBS + α-LDLR
OxPt/SN38 + α-LDLR
OxPt/SN38 + IgG
c
PBS + IgG
PBS + α-LDLR
OxPt/SN38 + α-LDLR
OxPt/SN38 + IgG

Figure S30 (a) Treatment schema and individual tumor growth curves of MC38 tumor-bearing C57BL/6 mice with PBS or OxPt/SN38 at a dose of 3.5 mg OxPt/kg equivalent with intratumorally injected 1 μg IgG or α-LDLR Ab. n = 6. Tumor weights (b) and photographs (c) of excised MC38 tumors on day 22, n = 6.

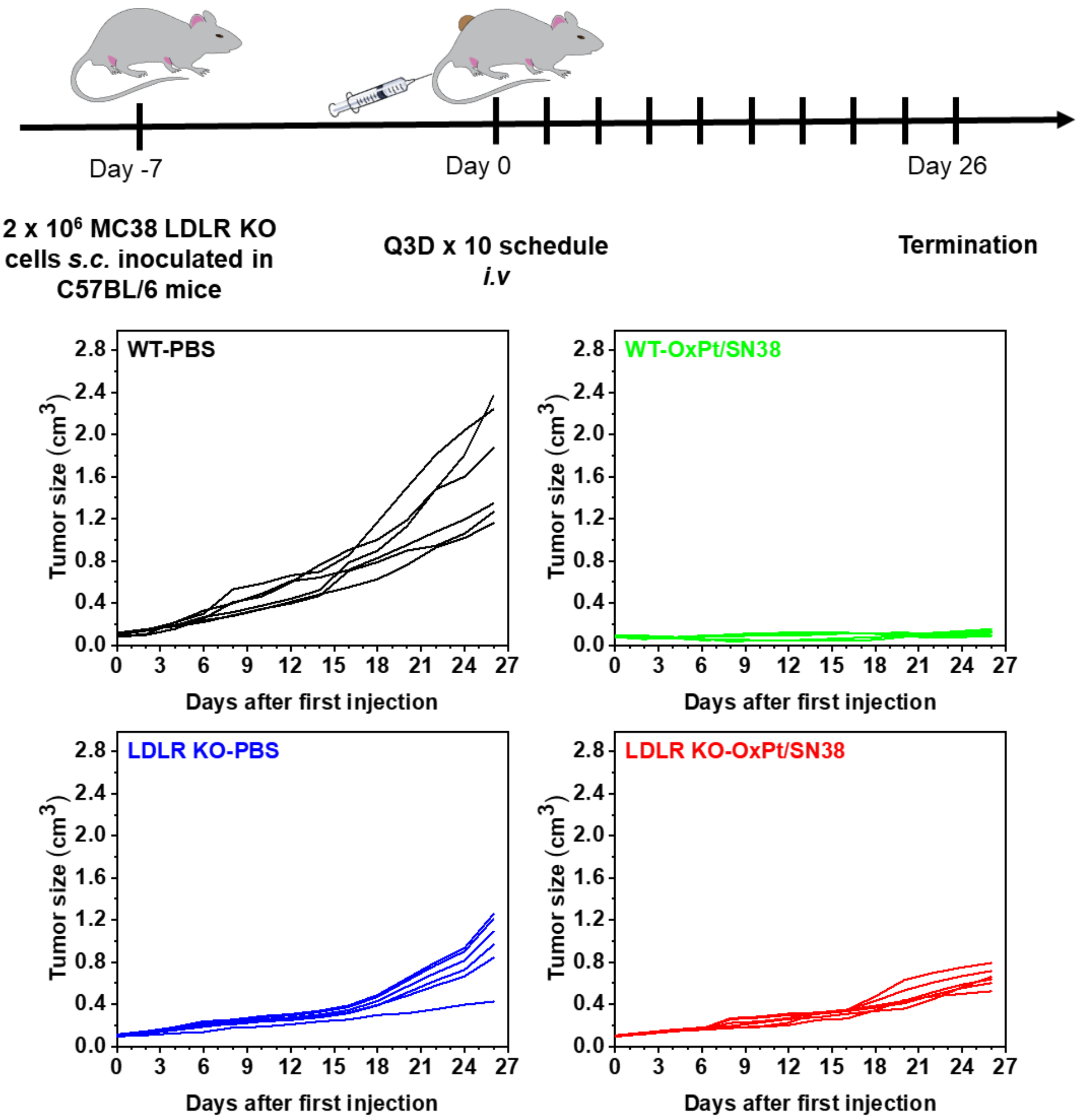

Figure S31 Treatment scheme and individual tumor growth curves of OxPt/SN38 on WT and LDLR KO MC38 tumor-bearing C57BL/6 mice at a dose of 3.5 mg OxPt/kg equivalent. n = 6.

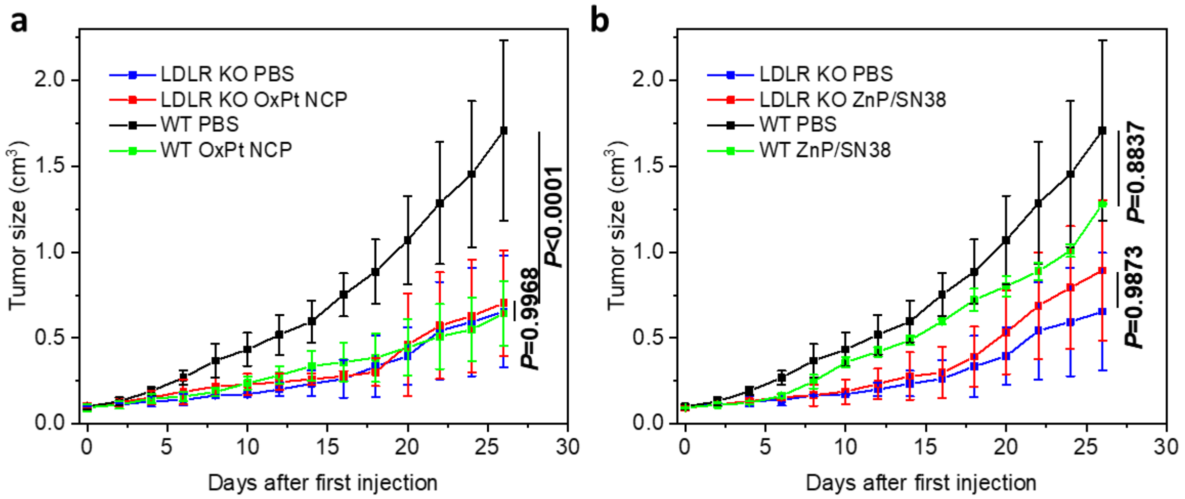

Figure S32 Anticancer efficacy of OxPt NCP (a), ZnP/SN38 (b) with MC38 WT and LDLR KO bearing C57BL/6 mice at the dose of 3.5 mg OxPt/kg (OxPt: SN38= 1:1.8). n = 3. Data are expressed as means ± SD.

Table S1. Size and PDI of NCPs with various formulations.

NPs	Size (d. nm)	PDI
OxPt Bare	48.98 ± 2.96	0.17 ± 0.03
OxPt/SN38	111.64 ± 0.40	0.15 ± 0.02
ZnP Bare	47.54 ± 1.56	0.16 ± 0.06
ZnP NCP	109.56 ± 5.59	0.20 ± 0.08
ZnP/SN38	114.65 ± 6.44	0.22 ± 0.11
OxPt NCP	108.27 ± 4.65	0.17 ± 0.07
OxPt NCP w/o chol	108.45 ± 10.45	0.25 ± 0.15
ZnP NCP w/o chol	102.59 ± 9.89	0.21 ± 0.14

Table S2. Binding constants of chol-SN38 and particles to plasma proteins

Drugs	Proteins	Binding affinity K_a (10^4 M^{-1})
OxPt/SN38	LDL	3.34 ± 1.30
OxPt/SN38	Albumin	0.00275 ± 0.00018
Chol-SN38	LDL	49.7 ± 2.43
Chol-SN38	Albumin	0.0225 ± 0.0144
OxPt NCP w/o chol	LDL	0.284 ± 0.020
Chol-SN38 micelle	LDL	2.76 ± 0.31

Table S3. Composition of the LDL slice.

Molecule	Number
POPC	64
Lyso PC	8
Cholesterol	60
Cholesterol oleate	160
Glyceryl trioleate	18

Table S4. Drug distribution percentage in different proteins in rat plasma (%)

	SN38	SN38-TMS	Chol-SN38	OxPt/SN38 (based on Chol-SN38)
VLDL	0.00	0.00	9.35 ± 0.56	19.39 ± 0.40
LDL	16.77 ± 2.94	11.69 ± 3.98	85.81 ± 0.91	74.13 ± 0.51
HDL	14.45 ± 1.82	7.09 ± 2.49	4.45 ± 0.40	6.08 ± 0.92
Albumin	68.78 ± 0.98	81.22 ± 1.48	0.39 ± 0.01	0.40 ± 0.01

Table S5. Chol-SN38 pharmacokinetics of SD/CD rats following a single intravenous injection of OxPt/SN38 at the dose of 5.56 mg/kg (based on SN38 injected) with non-compartmental analysis.

Parameter	Unit	OxPt/SN38*
Lambda_z	1/h	0.072 ± 0.007
t1/2	h	9.739 ± 0.992
Tmax	h	1.056 ± 1.684
Cmax	μg/ml	209.144 ± 98.855
C0	μg/ml	228.062 ± 122.083
Clast_obs/Cmax		0.018 ± 0.007
AUC 0-t	μg/ml*h	1874.570 ± 44.881
AUC 0-inf_obs	μg/ml*h	1924.399 ± 41.573
AUC 0-t/0-inf_obs		0.974 ± 0.010
AUMC 0-inf_obs	μg/ml*h^2	24547.320 ± 4376.051
MRT 0-inf_obs	h	12.789 ± 2.505
Vd	(mg/kg)/(μg/ml)	0.124 ± 0.013
Cl_obs	(mg/kg)/(μg/ml)/h	0.009 ± 0.000
Vss_obs	(mg/kg)/(μg/ml)	0.113 ± 0.024

*The value was analysis based on Chol-SN38.

Table S6. Tumor $AUC_{0\rightarrow t}$ biodistribution data

	Total Pt (h·μg/mL)	SN38 (h·μg/mL)
Free OxPt	59.7 ± 9.5	--
Irinotecan	--	8.5 ± 2.9
OxPt/SN38	290.3 ± 13.4	50.8 ± 4.2
OxPt/SN38 + α-LDLR	81.2 ± 18.7	5.1 ± 1.3

Table S7. OxPt and SN38 IC_{50} values (μM) in murine colon cancer cells treated with various formulations

	OxPt	SN38	Irinotecan	SN38-TMS	Chol-SN38	OxPt NCP	ZnP/SN38	OxPt/SN38	SN38-^{t}Bu	SN38-Boc
CT26	9.11 ± 0.72	0.22 ± 0.05	79.38 ± 6.94	2.52 ± 0.46	7.41 ± 1.03	8.01 ± 1.73	7.60 ± 0.20	1.96 ± 0.20	> 300	> 300
MC38	11.37 ± 1.68	0.24 ± 0.08	88.38 ± 7.48	3.04 ± 0.40	10.25 ± 1.99	9.17 ± 0.72	10.04 ± 0.85	2.74 ± 0.44	-	-

Table S8. OxPt and SN38 IC_{50} values (μM) in human colon cancer cells treated with various formulations

	OxPt	SN38	Irinotecan	SN38-TMS	Chol-SN38	OxPt/SN38
HT29	4.46 ± 1.94	0.058 ± 0.014	32.24 ± 4.00	0.448 ± 0.057	6.27 ± 0.42	1.24 ± 0.25
HCT116	2.19 ± 0.95	0.045 ± 0.007	16.57 ± 8.92	0.114 ± 0.020	3.83 ± 0.75	0.38 ± 0.09
SW480	5.64 ± 1.26	0.076 ± 0.026	45.34 ± 5.16	0.654 ± 0.162	7.37 ± 0.48	1.09 ± 0.43

Reference

[1] X. Duan, C. Chan, W. Han, N. Guo, R. R. Weichselbaum, W. Lin, *Nat. Commun.* **2019**, *10*, 1899.

[2] a) D. Sobot, S. Mura, S. O. Yesylevskyy, L. Dalbin, F. Cayre, G. Bort, J. Mougin, D. Desmaële, S. Lepetre-Mouelhi, G. Pieters, B. Andreiuk, A. S. Klymchenko, J.-L. Paul, C. Ramseyer, P. Couvreur, *Nat. Commun.* **2017**, *8*, 15678; b) S. O. Yesylevskyy, C. Ramseyer, M. Savenko, S. Mura, P. Couvreur, *Mol. Pharm.* **2018**, *15*, 585.

[3] T. Murtola, T. A. Vuorela, M. T. Hyvönen, S.-J. Marrink, M. Karttunen, I. Vattulainen, *Soft Matter* **2011**, *7*, 8135.

[4] A. K. Malde, L. Zuo, M. Breeze, M. Stroet, D. Poger, P. C. Nair, C. Oostenbrink, A. E. Mark, *J. Chem. Theory Comput.* **2011**, *7*, 4026.

[5] A. W. Sousa da Silva, W. F. Vranken, *BMC Res. Notes* **2012**, *5*, 367.

[6] L. Martínez, R. Andrade, E. G. Birgin, J. M. Martínez, *J. Comput. Chem.* **2009**, *30*, 2157.

[7] M. J. Abraham, T. Murtola, R. Schulz, S. Páll, J. C. Smith, B. Hess, E. Lindahl, *softwarex* **2015**, *1*, 19.

[8] S. Kumar, D. Bouzida, R. H. Swendsen, P. A. Kollman, J. M. Rosenberg, *J. Comput. Chem.* **1992**, *13*, 1011.

[9] M. Cassidy Shawn, W. Strobel Frank, M. Wasan Kishor, *Antimicrob. Agents Chemother.* **1998**, *42*, 1878.

[10] X. Duan, C. Chan, N. Guo, W. Han, R. R. Weichselbaum, W. Lin, *J. Am. Chem. Soc.* **2016**, *138*, 16686.
